# BRG1 (SMARCA4) Status Dictates the Response to EGFR Inhibitors in Wild-Type EGFR Non-Small Cell Lung Cancer

**DOI:** 10.3390/cancers18010062

**Published:** 2025-12-24

**Authors:** Rebaz Ahmed, Ranganayaki Muralidharan, Narsireddy Amreddy, Akhil Srivastava, Meghna Mehta, Janani Panneerselvam, Rodrigo Orlandini de Castro, William L. Berry, Susmita Ghosh, Murali Ragothaman, Pawan Acharya, Yan D. Zhao, Roberto Jose Pezza, Anupama Munshi, Rajagopal Ramesh

**Affiliations:** 1 Department of Pathology, The University of Oklahoma Health Sciences, Oklahoma City, OK 73104, USA; rebazawat@gmail.com (R.A.); ranganayaki.muralidharan@lily.com (R.M.); namreddy@cytovance.com (N.A.); askg2@health.missouri.edu (A.S.); janani@kopra.bio (J.P.); susmita-ghosh@ou.edu (S.G.); murali-ragothaman@ou.edu (M.R.); 2 Graduate Program in Biomedical Sciences, The University of Oklahoma Health Sciences, Oklahoma City, OK 73104, USA; 3 Department of Radiation Oncology, The University of Oklahoma Health Sciences, Oklahoma City, OK 73104, USA; megsmehta@gmail.com; 4 Cell Cycle and Cancer Biology Program, Oklahoma Medical Research Foundation, Oklahoma City, OK 73104, USA; orlandini.rodrigo@gmail.com (R.O.d.C.); roberto-pezza@omrf.org (R.J.P.); 5 Department of Surgery, The University of Oklahoma Health Sciences, Oklahoma City, OK 73104, USA; william-berry@ou.edu; 6 OU Health Stephenson Cancer Center, The University of Oklahoma Health Sciences, Oklahoma City, OK 73104, USA; daniel-zhao@ou.edu; 7 Biostatistics and Epidemiology, The University of Oklahoma Health Sciences, Oklahoma City, OK 73104, USA; pawanacharya@uabmc.edu

**Keywords:** lung cancer, EGFR, SMARCA4, BRG1, resistance, tyrosine kinase inhibitor, therapy

## Abstract

**Background**: Epidermal growth factor receptor (EGFR)-targeted tyrosine kinase inhibitors (TKIs) have exhibited efficacy in EGFR-mutant non-small cell lung cancer (NSCLC) patients. However, the response is modest in patients with wild-type (wt)-EGFR, and approximately 30–40% of patients develop TKI resistance. Recently, a role for BRG1 (SMARCA4) in regulating gene expression and its frequent alteration in various cancers, including NSCLC, has been reported. Yet, its specific function in response to EGFR-TKI therapy remains elusive. Herein, we investigated the role of BRG1 in EGFR-TKI response in vitro and in vivo using lung cancer models. **Methods**: In vitro, A549, H358, and HCC827 cell lines that varied in their EGFR and BRG1 status were assessed for response to EGFR-TKI upon overexpression or gene silencing of BRG1 through cell viability, cell migration, and Western blotting assays. In vivo, A549 and H358 tumor xenografts that overexpressed BRG1 or had BRG1 silenced were investigated for tumor growth response to EGFR-TKI. **Results**: EGFRwt/BRG1mt (A549) cells were resistant to TKI, and restoration of wt-BRG1 expression reverted them to TKI sensitivity both in vitro and in vivo. In contrast, silencing of BRG1wt in H358 cells showed a tendency toward TKI resistance. Additionally, wt-EGFR and pAKT^Ser473^ protein complex formation in A549 cells was disrupted with an AKT inhibitor (MK2206), resulting in enhanced cytotoxicity in vitro. **Conclusions**: Our study demonstrates that EGFR-TKI response in wt-EGFR cells is dictated by BRG1 status. These findings propose screening of wt-EGFR NSCLC patients for BRG1 status for identifying individuals likely to benefit from EGFR-TKI therapy versus patients who will benefit from AKT inhibitor treatment.

## Simple Summary

The availability of EGFR-targeted tyrosine kinase inhibitors (TKIs) has increased the survival of non-small cell lung cancer patients harboring EGFR mutations. In contrast, only a small patient population that is wild-type for EGFR responds to EGFR-TKIs. This discrepancy in response to TKIs warrants investigation. Recent studies implicate a role for BRG1 in gene expression and resistance to therapy. The study objective was to investigate EGFR-TKI response in wild-type EGFR lung cancer cells that varied in BRG1 status. We identified that BRG1 mutation influenced the response to EGFR-TKIs in EGFR wild-type lung cancer cells both in vitro and in vivo. Additionally, EGFR–AKT complex formation was shown to contribute to EGFR-TKI resistance in BRG1-mutant A549 cells. Incorporating the AKT inhibitor (MK2206) in EGFR-TKI-resistant cells showed enhanced cytotoxicity in vitro. Our study findings demonstrate that screening for BRG1 status in wild-type EGFR lung cancer patients will aid in identifying individuals who are likely to benefit from EGFR-TKI therapy.

## 1. Introduction

The advent of molecularly targeted small molecule inhibitors, exemplified by epidermal growth factor receptor (EGFR)-targeted tyrosine kinase inhibitors (TKIs), has notably improved treatment outcomes for patients diagnosed with non-small cell lung cancer (NSCLC) [1,2,3,4,5]. However, the efficacy of these EGFR-targeted therapies is confined to NSCLC patients harboring activating mutations within the intracellular kinase domain (IKD) of EGFR [6,7,8,9,10]. Despite initial responses to TKIs, a significant subset of patients develop treatment-related secondary mutations, leading to acquired resistance, relapse, and mortality [11,12,13,14]. Thus, ongoing efforts are focused on developing enhanced EGFR-TKIs that maintain effectiveness against EGFR-activating mutations while mitigating acquired resistance [15], resulting in the emergence of second- and third-generation EGFR-TKIs for NSCLC treatment [16,17,18,19]. In contrast, most NSCLC patients with wild-type (wt) EGFR exhibit inherent resistance to EGFR-TKIs [20,21,22,23,24,25]. We currently have only a limited understanding of the molecular variations defining this intrinsic resistance in wt-EGFR lung cancer cells. Evidence suggests that the interaction between the EGFR and AKT pathways is linked with intrinsic resistance [26,27,28,29], prompting the targeting of signaling networks that facilitate this interaction to enhance the efficacy of EGFR-TKIs. However, despite these efforts, objective responses remain elusive due to the activation of alternative kinases in the signaling cascade [21,30,31], highlighting that EGFR status alone is insufficient to determine sensitivity to EGFR-TKIs and suggesting the existence of novel, as-yet unexplored, mechanisms contributing to resistance.

The SWI/SNF (SWItch/Sucrose Non-Fermentable) family comprises a group of evolutionarily conserved complexes composed of multiple subunits crucial for chromatin structure remodeling in an ATPase-dependent manner [32,33]. In mammals, the three primary complexes, distinguished by their subunit composition, are the BRM-associated factor (BAF) complex, the polybromo-containing BAF (PBAF) complex, and the non-canonical BAF complex [34]. Each complex has a core subunit, either Brahma-related gene (BRG)1, also known as SMARCA4, or Brahma (BRM), also called SMARCA2, capable of ATP hydrolysis. These core subunits drive nucleosome sliding and removal from chromatin [35]. Given its role in chromatin stability and gene expression, the SWI/SNF complex is crucial for maintaining cellular homeostasis and mammalian development [36]. Thus, dysfunction of various subunits in the complex is frequently linked to human diseases, most notably cancer [37], with mutations and translocations occurring at a high frequency in multiple cancers [38,39,40]. Perturbation in these subunits lead to aberrant complexes, causing dysregulated transcriptional activation and repression of genes involved in various cellular processes, eventually contributing to tumorigenesis and resistance to therapy [41,42].

Recent evidence implicates that mutations in the SWI/SNF complex subunits can affect the response to anticancer therapies, including TKIs [43,44], with the loss of specific SWI/SNF subunits resulting in the upregulation of EGFR and other receptor tyrosine kinases (RTKs). For instance, loss of SMARCB1 leads to upregulation of EGFR and increased EGFR phosphorylation, thereby rendering rhabdoid tumor cells sensitive to EGFR-TKIs [45]. Concurring with these reports, loss of SWI/SNF subunits (e.g., SMARCE1, ARID1A, and BRG1) in NSCLC cell lines also results in elevated RTK levels, including EGFR [46]. These findings strongly suggest crosstalk between SWI/SNF complex subunits and EGFR signaling, potentially influencing responses to EGFR-TKIs. Thus, investigations into the crosstalk between SWI/SNF, in particular BRG1, due to its critical role as an ATPase core subunit [47,48,49,50,51,52,53], and EGFR pathways are warranted.

BRG1, encoded by the *SMARCA4* gene, has been reported to function both as a tumor suppressor and as an oncogene [54]. The dual functional nature of BRG1 is context-dependent and depends on the type of cancer studied. BRG1 is known to harbor inactivating mutations in various cancers, including lung cancer. For instance, loss-of-function mutations of BRG1 are found in about 30% of NSCLC cell lines, with approximately 10% of NSCLC tumors confirmed to exhibit BRG1 inactivating mutations, suggesting a tumor suppressor role [55]. In melanoma and acute myeloid leukemia, increased BRG1 expression was essential for cell proliferation and survival [42]. Recently, the dual activity of BRG1 as both a tumor suppressor and an oncogene was confirmed at distinct stages of pancreatic cancer formation and hepatocarcinogenesis [56,57]. Loss of BRG1 expression through impairment of active nucleosome positioning [40,46,58] leads to altered gene expression profiles and cancer development [46]. Consequently, investigations have delved into understanding the impact of BRG1 on cancer cell signaling pathways to elucidate its role in tumorigenesis. Studies have demonstrated that reintroducing wt-BRG1 into mutant (mt)-BRG1 tumor cell lines leads to re-expression of silenced genes and downregulation of genes that are overexpressed during NSCLC growth [59]. Additionally, the influence of BRG1 on EGFR expression has been explored. Fillmore et al. [60] suggested a negative correlation between BRG1 and EGFR mutations and a potential interaction between BRG1 and EGFR that is dependent on BRG1 status. Additionally, they showed that loss of BRG1 sensitized lung tumors to the Topoisomerase II inhibitor, etoposide, while tumors with wild-type BRG1 and EGFR status were resistant to etoposide [61]. The study results indicated that BRG1 and EGFR mutational status can affect treatment response. While the role for BRG1 alone in determining the response to chemotherapy and radiation therapy is established [61,62], how the BRG1-EGFR interaction and mutation status impact the effectiveness of targeted therapy remains unclear. These findings underscore the importance of elucidating the molecular mechanisms underlying BRG1 and EGFR interaction in the context of EGFR-TKI treatment, as understanding this interaction will improve treatment outcomes and mitigate the development of resistance to EGFR-TKIs.

In the current study, we investigated the impact of BRG1 mutation status on the response of wt-EGFR lung cancer cells to EGFR-TKIs and the associated molecular signaling mechanisms. Our results demonstrate that BRG1 acts as a negative regulator of wt-EGFR both in vitro and in vivo, and mutations in BRG1 lead to elevated expression of wt-EGFR and phosphorylated AKT (pAKT) upon EGFR-TKI treatment. Molecular analyses revealed a crosstalk between EGFR and BRG1, with the formation of a complex involving wt-EGFR and pAKT^(Ser473)^ influenced by BRG1 mutation status, thereby contributing to resistance. Notably, we found that NSCLC cells harboring wt-EGFR and mt-BRG1 exhibited heightened sensitivity to AKT inhibitors. Our findings emphasize the importance of pre-screening wt-EGFR NSCLC patients for BRG1 status, identifying two distinct subsets: those with wt-EGFR/wt-BRG1 who may benefit from EGFR-TKI therapy, and those with wt-EGFR/mt-BRG1 who might benefit from AKT inhibitors. In conclusion, our study highlights the potential of pre-screening for BRG1 status in wt-EGFR NSCLC patients to enable personalized treatment strategies to mitigate treatment-related resistance and enhance therapeutic outcomes.

## 2. Materials and Methods

### 2.1. Cell Lines

NSCLC cell lines (NCI-H358, A549, HCC827, NCI-H1975, Calu-3, NCI-H1299), normal human lung fibroblasts (MRC-9), and normal human bronchial epithelial primary cells (NHBE) were purchased from the American Type Culture Collection (ATCC, Manassas, VA, USA), and their identities confirmed by short tandem repeat (STR) analysis (Cell Line Genetics, Madison, WI, USA). The cell lines were determined to be mycoplasma-free by regular testing with the e-Myco Plus Mycoplasma PCR Detection Kit (ATCC, Rockville, MD, USA). All cells were grown in a 5% CO_2_ humidified incubator at 37 °C. NSCLC cell lines (H1299, NCI-H358, HCC827, Calu-3) were cultured in RPMI-1640 medium (Corning, NY, USA), A549 cells in Hams-F12 medium (Thermo Fisher Scientific, Waltham, MA, USA), and MRC-9 cells in Dulbecco’s modification of Eagle medium (DMEM, Corning, NY, USA), supplemented with 10% heat-inactivated fetal bovine serum (FBS) and 1% penicillin/streptomycin (Sigma Aldrich, St. Louis, MO, USA). NHBE were maintained in epithelial cell basal medium supplemented with bronchial/tracheal epithelial cell growth kit (ATCC, Manassas, VA, USA). The passage numbers for tumor cells, MRC-9, and NHBE used in the study were 8 to 35, 4 to 12, and 4 to 8, respectively.

### 2.2. Liposome Synthesis and siRNA Complex Formation

Liposomes were synthesized using DOTAP lipid and cholesterol (Avanti Polar Lipids, Alabaster, AL, USA) through the thin-film hydration method and extruded through different sizes of filters, as reported previously [63,64,65]. For siRNA-liposome complex preparation, appropriate concentrations of siRNAs targeting human BRG1, EGFR, and control siRNA were complexed with DOTAP:Chol and characterized prior to use in the studies as previously described [63,64,65].

### 2.3. siRNA Delivery and Cell Viability Assay

NSCLC (A549, NCI-H358) cells were seeded (1 × 10^5^ cells/well) in six-well plates and allowed to grow for 24 h. Before transfection, cells were starved in serum-free RPMI-1640 medium or Hams-F12 medium for one hour and then transfected using the siRNA-liposome complex to deliver BRG1-siRNA (siBRG1; 100 nM), EGFR-siRNA (siEGFR; 50 nM), and control-siRNA (C-siRNA; 100 nM) (Dharmacon, Lafayette, CO, USA). Cells were transfected with siRNA-containing liposome. After 6 h of treatment, the medium was replaced with 2% FBS-containing Hams-F12 or RPMI-1640 medium. Untransfected cells served as controls. Cells were harvested at 24 h and 48 h post-treatment, and the number of viable cells was counted by Trypan blue exclusion assay as previously described [66,67]. Cell viability results were expressed as the percentage of viable cells over untreated control cells.

### 2.4. Generation of BRG1 Overexpressing and Knock-Out (KO) Cell Lines

The retroviral BRG1 overexpressing plasmid, pBABE-BRG1 (Addgene: 1959), and an empty control vehicle plasmid, pBABE-Puro (Addgene:1764), were purchased from Addgene [68,69] and used for retrovirus vector production. NSCLC cell lines (A549 and NCI-H358) were infected with pBABE-BRG1 or pBABE-Puro carrying retrovirus constructs and selected with puromycin (4 µg/mL) for two weeks according to standard procedures [70]. The puromycin-selected cells were labeled as A549-BRG1, H358-BRG1, A549-Puro and H358-Puro cell lines, respectively. Overexpression of BRG1 in A549-BRG1 and H358-BRG1 cells was confirmed by qRT-PCR and Western blot analysis, as previously described [64,66]. H358-Puro, A549-Puro, NCI-H358, and A549 cell lines were used as controls in the studies.

NCI-H358 BRG1 knock-out cells (H358-BRG1-KO) were generated by transfecting with Prp [CRISPR]-hCas9-U6>hSMARCA4_10_66984_20nt] vector, a gift from Roberto Pezza’s laboratory, carrying the following BRG1-targeting sequence: GAAGATTACTTTGCGTATCG (VectorBuilder, Chicago, IL, USA). pCas-Scramble (OriGene, Rockville, MD, USA), which expresses human codon-optimized Cas9, and a scrambled guide RNA (gRNA) were used as controls. Cells were transfected using lipofectamine 3000 (Thermo Fisher Scientific, Waltham, MA, USA). At 72 h post-transfection, the cells were selected with puromycin (4 μg/mL) for two weeks, and single-cell clones grown and analyzed by Western blotting for BRG1 expression. Alteration in the target sequence of the BRG1 gene was verified with Sanger sequencing, as previously described [71]. The sequencing primers used are shown below.

Human BRG1
Forward 5′ GCCTGCAGGGTTCCAGGTTTA
Reverse 5′ GAAACGCCTCATGGCTCATAC

### 2.5. Western Blotting

Total protein was isolated from NSCLC cells following various treatments using the radioimmunoprecipitation assay (RIPA) lysis buffer supplemented with protease and phosphatase inhibitors (Sigma Aldrich, St. Louis, MO, USA) and subjected to Western blotting as previously described [66,67]. Briefly, 60–80 µg of total protein separated on 7.5% SDS polyacrylamide gel was transferred to a polyvinylidene fluoride (PVDF) membrane (Immobilon^®^, Millipore, MA, USA). Following transfer, membranes were blocked for one hour in 5% fat-free milk in 1X Tris-buffered saline with Tween 20^®^ (TBST, pH 7.5; Thermo Fisher Scientific, Waltham, MA, USA). The membranes were then incubated overnight with anti-human primary antibodies (Table S1) in 5% BSA in TBST according to the manufacturer’s recommendation. Following several washes in TBST buffer, the membranes were incubated with appropriate horseradish peroxidase-(HRP)-tagged secondary antibodies (Santa Cruz Biotechnology, Dallas, TX, USA). Protein bands were detected using an enhanced chemiluminescence kit (Thermo Fisher Scientific, Waltham, MA, USA). Protein expression levels were detected on a chemiluminescence imaging system (Syngene, Frederick, MD, USA), and the relative protein expression compared to α-tubulin or β-actin was quantified using Gene Tools software, version 4.02 (Syngene) [65,66,67].

### 2.6. Quantitative Real-Time Polymerase Chain Reaction (qRT-PCR)

Total RNA was isolated from NSCLC cells receiving various treatments using TRIZOL reagent (Invitrogen, Carlsbad, CA, USA) following the manufacturer’s instructions, and qRT-PCR was performed as previously described [65,66]. Briefly, cDNA was synthesized using 1 μg of total RNA and Quant script cDNA synthesis kit (Bio-Rad, Hercules, CA, USA). Subsequent qRT-PCR reactions were performed in a 20 µL volume containing 120 nM of each primer, 1 µL of diluted cDNA, and 10 µL of FastStart iQTM SYBR Master Mix (Bio-Rad). The oligonucleotide primers used are listed in Table 1. The relative change in mRNA expression relative to untreated control was represented as fold change calculated as 2^−ΔΔCt^, using a threshold cycle (Ct) for each reaction/condition. 18S rRNA transcript was used as a housekeeping gene for normalization in each reaction.

### 2.7. Flow Cytometry

NCI-H358 parental and H358-BRG1-KO cells were harvested at 75% confluency and fixed with 4% paraformaldehyde (Alfa Aesar, Ward Hill, MA, USA). Cells were washed three times with ice-cold PBS, then resuspended in staining buffer (Thermo Fisher Scientific) and incubated with Fc Receptor Binding Inhibitor Polyclonal Antibody (Thermo Fisher Scientific) (1 µL/10 µL of cell suspension) for 20 min with gentle shaking at 4 °C. The conjugated antibodies against human EGFR (Anti-EGFR Alexa Fluor^®^ 488; Abcam; Cambridge, MA, USA) and phosphorylated (p)EGFR (Anti-EGFR phospho (Tyr1068) Alexa Fluor^®^ 488; Abcam), or control rabbit IgG (Alexa Fluor^®^ 488; Abcam), were added for one hour with gentle shaking at 4 °C and protected from light. Cells were washed three times with staining buffer and subjected to flow cytometry analysis using FACS Calibur as previously described [63,72]. Samples were gated under the same parameters.

### 2.8. Immunoprecipitation

H358-BRG1-KO and A549 parental cells treated with EGFR inhibitor, gefitinib (2 µM; Selleckchem, Houston, TX, USA), or osimertinib (500 nM; Selleckchem) and AKT inhibitor, MK2206 (0.25 µM; Selleckchem) were harvested at 24 h after treatment and subjected to immunoprecipitation (IP) as previously described [73]. Antibodies specific to human EGFR and AKT (Cell Signaling Technology, Danvers, MA, USA) were used for IP and Western blotting (Table S1). The fraction of the cell lysate not subjected to IP served as input control along with flow-through samples. Heavy chain IgG, IgG (HC), served as an internal control. Cells receiving no treatment served as controls in these studies.

### 2.9. Immunofluorescence

NCI-H358 (5 × 10^4^ cells/well) cells were treated with 100 nM C-siRNA or siBRG1 complexed in DOTAP:Chol liposome for 48 h on coverslips placed in six-well plates. Cells were then fixed in 4% paraformaldehyde and permeabilized with 0.1% Triton X-100 (Thermo Fisher Scientific) for 3 min. After three 5 min rinses in PBS, the cells were incubated in blocking buffer (5% goat serum in 2% BSA) (Sigma Aldrich, St. Louis, MO, USA) for 15 min. Next, the cells were incubated with conjugated antibodies against EGFR (Anti-EGFR, Alexa Fluor^®^ 488; Abcam) and BRG1 (Anti-BRG1, Alexa Fluor^®^ 647; Abcam) at a dilution of 1:500 and 1:1000, respectively, in 5% BSA overnight at 4 °C. Rabbit IgG (Alexa Fluor^®^ 488; Abcam) at a dilution of 1:500 was used as a control. The cells were then washed three times in PBS. Nuclei were counterstained with 4′,6-diamidino-2-phenylindole dihydrochloride (DAPI, 1 μg/mL) in PBS for 5 min. Then coverslips were mounted on slides with Vectashield (Vector Laboratories, Burlingame, CA, USA). Slides were examined on the Leica SP2 MP confocal microscope (Leica Microsystems, Deerfield, IL, USA). Images were imported into ImageJ, version 1.52 (NIH) analysis software.

### 2.10. Subcellular Fractionation Assay

The membrane-bound, cytoplasmic, and nuclear fractions of EGFR, pEGFR^(1068)^, AKT, and pAKT^(Ser473)^ were extracted from H358-Scr (scramble) and H358-BRG1-KO cells. The subcellular fractions were separated using Subcellular Protein Fractionation Kit (Thermo Fisher Scientific) following the manufacturer’s protocol. Briefly, 2 × 10^6^ cells were harvested and washed with ice-cold PBS. Cells were sequentially resuspended in Cytoplasmic Extraction Buffer (CEB), Membrane Extraction Buffer (MEB), and Nuclear Extraction Buffer (NEB) at 200:200:100:100 μL, respectively. Cells were washed with ice-cold PBS between each extraction to avoid contamination. The separation of each fraction was performed with centrifugation at 500× *g*, 3000× *g*, and 5000× *g* for 5 min, respectively, at 4 °C. The cellular fractions were then loaded on 7.5% SDS polyacrylamide gel and subjected to Western blotting. Lamin-B1 and α-tubulin were used as loading controls. The antibodies used to detect the markers of interest are listed in Table S1.

### 2.11. Migration and Invasion Assay

Cell migration and invasion assays were carried out as previously described [66,73]. Briefly, NCI-H358 and H358-BRG1-KO (4 × 10^4^) cells were seeded in the upper chamber of the transwell for migration assay, and in collagen-coated chambers for invasion assay (8 μm; BD. Biosciences, Bedford, MA, USA) and were placed in individual wells of six-well plates filled with 1 mL of serum-free RPMI-1640 medium. Cells were allowed to adhere for 24 h in the upper chamber and then treated with 2 µM and 20 µM GEF gefitinib. After 24 h, the culture medium in the upper and lower chambers was replaced with 2% and 20% serum-containing medium, respectively. At 24 h and 48 h after incubation, the inserts were removed and stained with crystal violet (Sigma-Aldrich). The number of migrated and invaded cells was counted using an inverted bright-field microscope, and the results expressed as the average number of migrated or invaded cells per microscopic field.

### 2.12. Single and Combination Treatment of NSCLC Cells In Vitro

EGFR-TKIs—gefitinib and osimertinib, BRG1-bromodomain inhibitor (PFI-3), and an AKT inhibitor (MK-2206). NSCLC cells (A549, A549-Puro, A549-BRG1, NSCI-H358, H358-Scramble, H358-BRG1-KO, and HCC827) (1 × 10^5^ cells/well) were seeded in six-well plates and allowed to grow for 24 h in appropriate complete culture medium. Cells were treated with either DMSO or various concentrations of gefitinib (40 nM, 0.5 µM, 1.0 µM, 2.0 µM, 10 µM, and 20 µM GEF), osimertinib (15 nM and 500 nM OSI), a fixed concentration of PFI-3 (10 µM; Selleckchem), or MK-2206 (0.25 µM; Selleckchem), in 2% FBS-containing culture medium. At 24 and 48 h post-treatment, cells were harvested and subjected to cell viability assays, and cell lysates were prepared for molecular analysis by Western blotting.

EGFR stimulation with its ligand, EGF. NCI-H358 cells (1 × 10^5^ cells/well) were seeded in six-well plates and allowed to grow for 24 h in 10% FBS-RPMI-1640-containing medium. Cells were serum-starved for one hour, followed by treatment with recombinant human EGF (50 nM) (Thermo Fisher Scientific). Cells were harvested at 30 min, 1 h, and 3 h post-treatment and cell lysates were prepared and subjected to Western blot analysis to detect the protein markers listed in Table S1.

Combination treatment (siBRG1 and gefitinib (GEF). NSCLC cells (A549, A549-BRG1, and NCI-H358) (7 × 10^4^ cells/well) were seeded in six-well plates and allowed to grow for 24 h in 10% FBS-containing culture medium. Cells were starved for one hour in serum-free culture medium, then liposomes containing siBRG1 (100 nM) or C-siRNA (100 nM) were added to the cells. After 6 h of incubation, the medium was replaced with 2% FBS-containing culture medium. At 24 h post-siRNA treatment, the cells were treated with 1 µM gefitinib. Cells receiving DMSO or C-siRNA treatment, and cells receiving no treatment, served as controls. At 24 and 48 h post-gefitinib treatment, the cells were harvested for cell viability and Western blot analyses. The final treatment groups included in this experiment were DMSO, C-siRNA, siBRG1 (100 nM), gefitinib (1 µM GEF), gefitinib plus C-siRNA, and gefitinib plus siBRG1.

Combination treatment (PFI-3 and gefitinib): NCI-H358 cells (7 × 10^4^ cells/well) were seeded in six-well plates and allowed to grow for 24 h in RPMI-1640 medium containing 10% FBS. The medium was replenished with 2% FBS RPMI-1640 medium containing PFI-3 (10 µM). After 6 h of incubation, gefitinib (2 µM GEF) was added to the cells. Cells receiving DMSO treatment served as controls. The cells were harvested for cell viability and Western blotting analyses 24 and 48 h post-combination treatment.

Combination treatment (PMX-BRG1 and gefitinib): H358-BRG1-KO cells (5 × 10^4^ cells/well) were seeded in 6-well plates and cultured for 24 h in RPMI-1640 containing 10% FBS. Cells were serum-starved for one hour before treatment with liposomes containing PMX-BRG1 (2 µg) (Addgene: 25855) or PMX-empty (a gift from Dr. William Berry’s laboratory). After 6 h of incubation, the medium was replaced with RPMI-1640 medium containing 2% FBS. At 72 h post-transfection, cells were treated with gefitinib (2 µM). Cells receiving DMSO or PMX-empty vector served as controls. At 24 and 48 h post-gefitinib treatment, the cells were harvested and analyzed for cell viability and by Western blotting. The final treatment groups in this experiment included DMSO, PMX-Empty (2 µg), gefitinib (2 µM GEF), gefitinib (2 µM GEF) plus PMX-empty (2 µg), PMX-BRG1 (2 µg), and gefitinib (2 µM GEF) plus PMX-BRG1 (2 µg).

Combination treatment (gefitinib, osimertinib and MK-2206): H358-BRG1-KO and A549 parental cells (1 × 10^5^ cells/well) were seeded in six-well plates and allowed to grow for 24 h in 10% FBS-containing culture medium, following which the medium was replaced with MK-2206 (0.25 µM) containing 2% FBS culture medium. After 24 h of incubation, gefitinib (2 µM or 10 µM GEF) was added to the cells. Cells receiving DMSO treatment served as controls. The cells were harvested at 24 and 48 h post-gefitinib treatment and subjected to cell viability and Western blot analyses.

The combinatorial cytotoxic effects of osimertinib and MK-2206 were determined in A549 cells. The osimertinib and MK-2206 concentrations used were 500 nM and 0.25 µM, respectively. All other parameters, including the experimental design and end point analysis for cell viability, remained the same as described above for gefitinib and MK-2206 combination studies.

### 2.13. Tumor Xenograft Studies

A549-Puro and A549-BRG1 (5 × 10^6^/100 µL PBS) subcutaneous tumor xenografts were established on the lower-left flank of 4- to 6-week-old female nude mice (Nu-Nu), purchased from Charles River Laboratories (Wilmington; MA, USA). H358-Scr and H358-BRG1-KO contralateral tumors were established by injecting 1 × 10^7^ cells suspended in 100 µL of PBS into the lower-right and left flanks of 4- to 6-week-old female nude mice (Nu-Nu), purchased from Envigo (Madison, WI, USA). When the tumors reached about 80–100 mm^3^, the mice were randomized and assigned to treatment groups after sorting based on tumor volume. The number of mice was five per group for the A549 tumor model and seven per group for the H358 tumor model, with each tumor model containing two groups. The control group received 100 µL of vehicle (10% DMSO in sterile PBS), and the treatment group received 100 mg/kg of gefitinib. DMSO and gefitinib were administered intraperitoneally (i.p.) every 3 days for a total of four treatments. The tumor size was measured every 3 days for 10 weeks with calipers. The tumor volume was calculated using the formula (volume = (length × width^2^)/2) as previously described [63]. At the end of the experiment, mice were euthanized, and the A549-Puro and A549-BRG1 tumors were collected for molecular and immunohistochemical analysis. The tumors from the H358-Scr and H358-BRG1-KO were not collected because the BRG1-KO tumors grew slowly and became necrotic when smaller than 100 mm^3^ in size.

### 2.14. Immunohistochemistry

A549-Puro and A549-BRG1 tumor xenografts from vehicle and gefitinib-treated groups were harvested and fixed overnight in 4% paraformaldehyde. Immunohistochemistry (IHC) was performed on the Leica Bond RX platform using the Polymer Refine Detection System (Leica Bond-III, Leica Biosystems, Nussloch, Germany). In brief, Formalin-Fixed Paraffin-Embedded (FFPE) tissues were sectioned at the desired thickness (4–8 µm) and mounted on positively charged slides. After drying overnight at room temperature, the slides were incubated at 60 °C for 45 min. Slides were transferred to the Leica Bond RX for dewaxing and then incubated at 100 °C for 20 min in an antigen retrieval solution (pH 6.0). The sections were incubated with 5% goat serum (Sigma Aldrich) for 30 min. Endogenous peroxidase was blocked using a peroxidase-blocking reagent, followed by the selected primary antibody incubation for 60 min. For the secondary antibody, a post-primary IgG linker and/or poly-HRP IgG reagent was used. Detection was achieved using 3,3′-diaminobenzidine tetrahydrochloride (DAB) as the chromogen and counterstained with hematoxylin. The completed slides were dehydrated (Leica ST5020) and mounted (Leica MM24). Antibody-specific positive and negative controls (omission of primary antibody) were stained in parallel. The stained tissue sections were analyzed using the Aperio Scan Scope Image Analysis System. Stained tumor tissues were analyzed using the Positive Pixel Count algorithm with the Aperio Image Scope viewer [74]. Antibodies used to stain the tissues are listed in Table S1. Similar staining and quantification procedures were followed for human lung (healthy and NSCLC) Tissue Microarray (TMA; US Biomax; MD, USA).

### 2.15. Statistics

Data obtained from this study were subjected to statistical analysis using the SAS 9.4 software (SAS Institute Inc., Cary, NC, USA). Experimental variables are expressed as the mean ± standard deviation (S.D.). One-way analysis of variance (ANOVA) was performed to assess differences across groups, with Tukey’s adjustment for pairwise comparisons. Adjusted *p*-values less than 0.05 were considered statistically significant. Graphs were prepared using GraphPad Prism, version 9.3.1.

### 2.16. Ethics Approval and Consent to Participate

Animal protocol (101577-16-062-HC) was reviewed and approved (05 August 2016) by the Institutional Animal Care and Use Committee (IACUC) at the University of Oklahoma Health Sciences Center (Oklahoma City, OK, USA).

## 3. Results

### 3.1. Gefitinib Treatment Induces BRG1 in wt-EGFR NSCLC Cell Lines In Vitro

Our initial investigation aimed to assess the impact of gefitinib treatment on BRG1 in two NSCLC cell lines (NCI-H358 and A549), both of which had wt-EGFR but differed in their BRG1 status. Treatment with clinically relevant concentrations of gefitinib (0.5 µM, 1.0 µM, 2 µM GEF) resulted in the suppression of pEGFR^Tyr1068^ in both NCI-H358^(wt-EGFR/wt-BRG1)^ and A549^(wt-EGFR/mt-BRG1)^ cell lines across all tested drug concentrations (Figure S1A). However, while total EGFR levels decreased in NCI-H358 cells, there was no significant change in A549 cells. BRG1 and BRM protein expression analysis revealed an increase in BRG1 protein levels with no noticeable change in BRM expression in the two cell lines. These results suggested a potential interaction between BRG1 and EGFR, influenced by BRG1′s mutational status, and formed the basis for further investigation into the EGFR and BRG1 interaction in gefitinib-treated cells.

### 3.2. Genetic and Pharmacologic Modulation of wt-BRG1 Negatively Regulates EGFR Expression in NSCLC Cell Lines In Vitro

**Figure 1:**
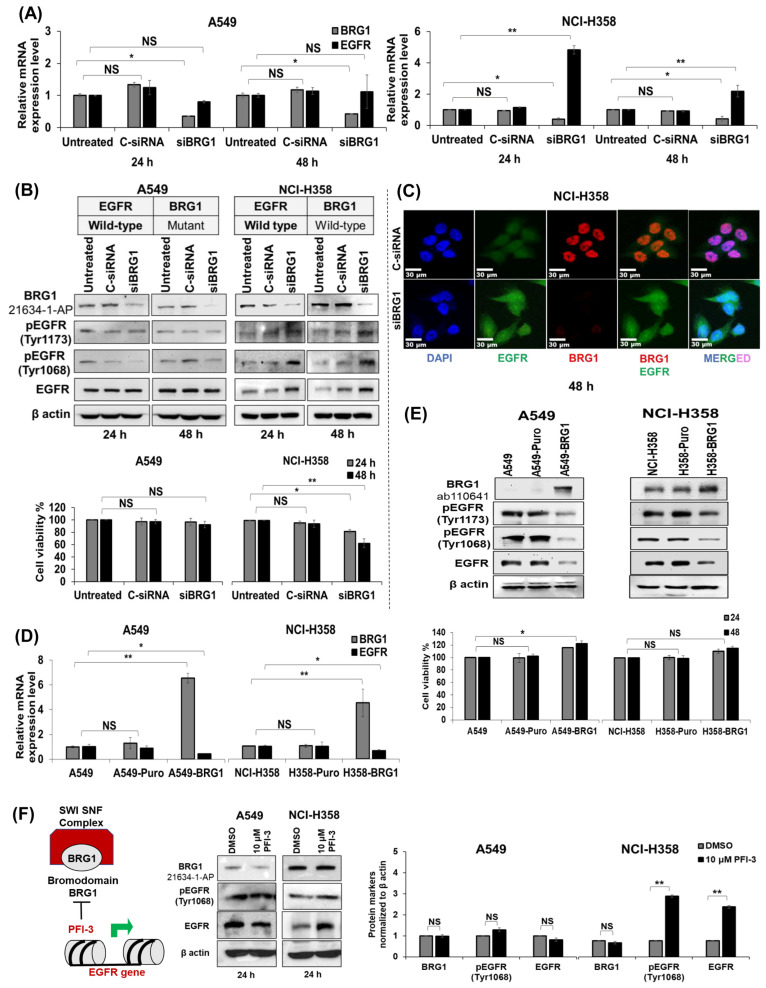
Genetic and pharmacologic modulation of BRG1 and its impact on EGFR expression in A549 and NCI-H358 cells. (**A**) mRNA expression of BRG1 and EGFR in siBRG1-treated A549 and NCI-H358 cells. mRNA expression level in siBRG1 treated cells was determined by qRT-PCR at 24 and 48 h after treatment and compared with C-siRNA and untreated control cells. siBRG1 and CsiRNA concentrations used were 100 nM. (**B**) siBRG1-treated cells were analyzed for protein expression (upper panel) and cell viability (lower panel) and compared to C-siRNA-treated and untreated control cells at 24 and 48 h. β-Actin was used as a loading control. (**C**) Immunofluorescence study showing increased EGFR expression in siBRG1-treated NCI-H358 cells. Cells were treated with C-siRNA and siBRG1 and stained for BRG1 (red), EGFR (green), and DAPI (blue) at 48 h after treatment. Representative images for each treatment are shown. Magnification, 40×; Scale bar, 30 μm. (**D**) BRG1 and EGFR mRNA expression in BRG1-overexpressing A549 and NCI-H358 cells. The qRT-PCR analysis showed BRG1 mRNA increased in BRG1-overexpressing cells compared to vector control (Puro) and parental cells. (**E**) Western blot analysis of exogenous BRG1 and its impact on reducing the expression of EGFR and phosphorylated (p) EGFR in A549-BRG1 and H358-BRG1 cells. β-Actin was used as a loading control. A549, A549-Puro, NCI-H358, and H358-Puro cells were used as controls, respectively. Corresponding cell viability is shown in bar graphs below the Western blots. (**F**) Schematic showing the mode of action of the BRG1 inhibitor PFI-3, and data showing inhibitory effects of PFI-3 (10 µM) on BRG1. A549 and NCI-H358 cells treated with PFI-3 or DMSO were analyzed for BRG1 and EGFR at 24 h after treatment by Western blot analysis. β-Actin was used as a loading control. The semi-quantification of protein levels is shown in the bar graphs to the right of the Western blots (*n* = 3). The original Western blot images of Figure 1B,E,F are provided in the Supplementary Figure S6. Error bars denote SD; NS = not significant; * *p* < 0.05; ** *p* < 0.01.

We next examined the effects of both genetic and pharmacologic inhibition of BRG1 on EGFR expression in NCI-H358^(wt-EGFR/wt-BRG1)^ and A549^(wt-EGFR/mt-BRG1)^ cells. siRNA-mediated genetic inhibition of BRG1 (100 nM siBRG1) attenuated BRG1 mRNA and protein expression in both cell lines at two tested time points (Figure 1A,B), while treatment with control (C) siRNA (100 nM) had no effect on BRG1 mRNA or protein expression. Surprisingly, siBRG1 treatment significantly increased EGFR expression at both the mRNA and protein levels, including pEGFR^Tyr1173^, pEGFR^Tyr1068^, and total EGFR in NCI-H358 cells (*p* < 0.05; Figure 1A,B and Figure S1B). In contrast, in A549 cells, siBRG1 treatment reduced BRG1 mRNA but did not significantly increase EGFR mRNA or protein expression (Figure 1A,B and Figure S1B). The uncropped original Western blots are shown in Figure S6. Furthermore, siBRG1 treatment notably reduced the viability of NCI-H358 cells at both tested time points compared to C-siRNA and untreated controls (Figure 1B; lower panel, *p* < 0.05) but did not significantly affect A549 cell viability (Figure 1B; lower panel). Immunofluorescence studies confirmed that siBRG1 treatment, but not C-siRNA, increased EGFR expression in NCI-H358 cells (Figure 1C), an observation that was consistent with our Western blot data. These findings collectively suggest that wt-BRG1 plays a critical role in regulating EGFR expression, supporting previously published data that demonstrated BRG1 binding at the EGFR regulatory element is associated with transcriptional repression of EGFR, as evidenced by reduced EGFR levels in BRG1-overexpressing cells [60].

To further corroborate our findings, we examined EGFR expression in BRG1-overexpressing NCI-H358 (H358-BRG1) and A549 (A549-BRG1) cells and compared them to vector control (H358-Puro and A549-Puro) and parental cells. BRG1 overexpression significantly reduced EGFR mRNA (Figure 1D; *p* < 0.05) and protein (Figure 1E and Figure S1C; *p* < 0.05) levels in both A549 and NCI-H358 cells. The original Western blots are presented in Figure S6. Furthermore, cell viability studies revealed that BRG1 overexpression significantly increased the numbers of A549 cells but not NCI-H358 cells (Figure 1E; lower panel).

We then proceeded to assess the inhibitory effect of PFI-3, a pharmaceutical compound that hampers BRG1 activity by interacting with its bromodomain (BRD) [74], as illustrated in Figure 1F. The uncropped original Western blots can be found in Figure S6. Treatment with PFI-3 at a concentration of 10 µM notably increased the pEGFR^Tyr1068^ and total EGFR levels in NCI-H358 cells (*p* < 0.05). In contrast, no significant increase was observed in A549 cells. The absence of elevated EGFR expression in PFI-3-treated A549 cells can be attributed to PFI-3′s inability to bind to the truncated BRG1 protein lacking the BRD region [75,76]. Collectively, these data indicate that EGFR expression depends on the mutational status of BRG1 in NSCLC cell lines. 

### 3.3. Effect of Pharmacologic and Genetic Inhibition of EGFR on BRG1

**Figure 2:**
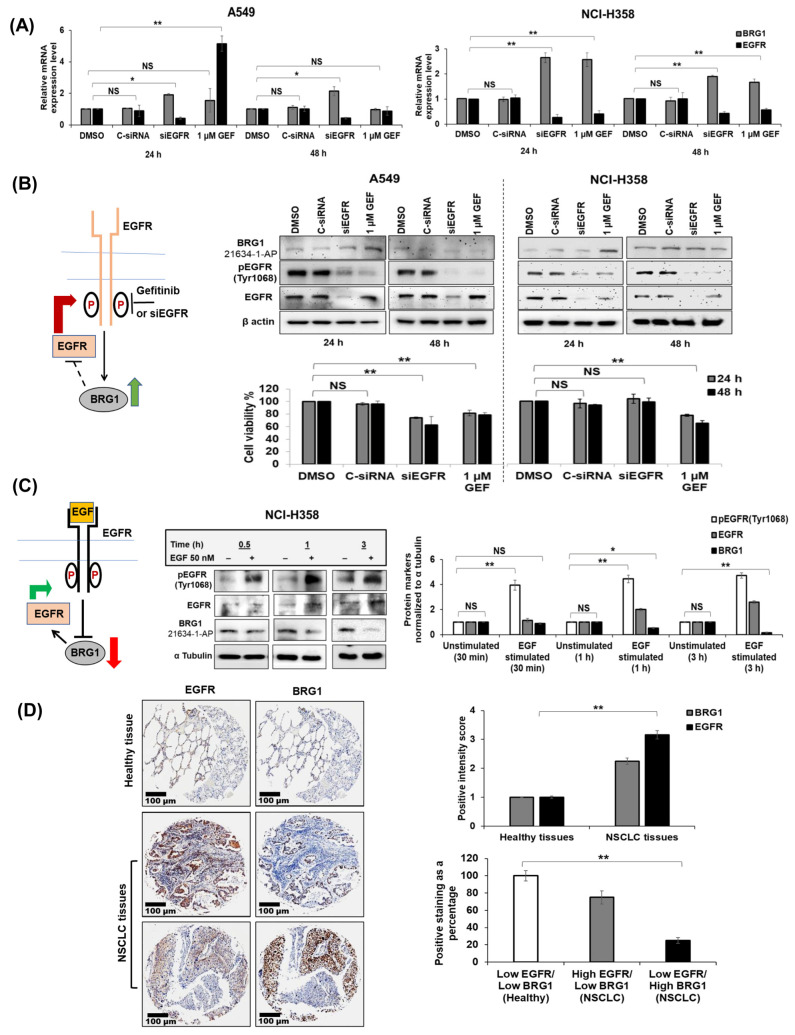
The inverse correlation between BRG1 and EGFR is demonstrated through genetic and pharmacological modulation of wt-EGFR and its impact on BRG1 expression in cell lines and lung tumor tissue microarray (TMA). A549 and NCI-H358 cells treated with siEGFR (50 nM) and gefitinib (1 µM GEF) were analyzed for EGFR and BRG1 mRNA (**A**) and protein expression (**B**) at 24 and 48 h after treatment. DMSO and control C-siRNA (50 nM)-treated cells served as controls. β-Actin served as a protein loading control in Western blotting. Corresponding cytotoxic effects of siEGFR and gefitinib are depicted in bar graphs against each cell line. (**C**) Western blot analysis of selected markers in NCI-H358 cells stimulated with human recombinant EGF (50 nM) for 30 min, 1 h, and 3 h. Unstimulated cells were used as controls. α-Tubulin served as a loading control. Semi-quantitative analysis of Western blot results is shown in bar graphs (*n* = 3). The bar graph (right panel) depicts the quantitative analysis of the proteins. (**D**) Immunohistochemical staining of NSCLC TMA demonstrates an inverse correlation for EGFR and BRG1 expression in NSCLC. Bar graphs depict the positive intensity score and the percentage of tissues expressing EGFR and BRG1 with intensity scores <2 (low expression) and >2 (high expression). Representative images of healthy and NSCLC tissues are shown. Magnification, 40 X; Scale bar, 100 μm. The original Western blot images are provided in the Supplementary Figure S6. Error bars denote SD; NS = not significant; * *p* < 0.05. ** *p* < 0.01.

Since inhibiting BRG1 increased EGFR expression, we performed a reverse experiment to investigate how EGFR inhibition affects BRG1 expression levels. A549 and NCI-H358 cells were treated with human EGFR-specific siRNA (siEGFR; 50 nM), control siRNA (CsiRNA; 50 nM), or gefitinib (1 µM GEF) and analyzed for mRNA and protein expression and cell viability at 24 and 48 h after treatment. DMSO-treated cells served as controls. siEGFR treatment reduced both EGFR mRNA and protein levels, including pEGFR^Tyr1068^ and total EGFR, in both cell lines at the two tested time points (Figure 2A,B and Figure S1D). The original Western blots can be found in Figure S6. Conversely, while siEGFR treatment increased BRG1 mRNA levels, no corresponding increase in BRG1 protein expression occurred in either cell line. However, gefitinib treatment significantly increased BRG1 mRNA and protein levels in NCI-H358 cells with a concomitant reduction in pEGFR^Tyr1068^ and total EGFR (Figure 2A,B and Figure S1D; *p* < 0.05). In A549 cells, gefitinib treatment produced varying effects on BRG1 and EGFR; while pEGFR^Tyr1068^ protein expression was reduced, there was no change in total EGFR protein expression at either 24 or 48 h, although BRG1 protein levels were higher at 24 h but not at 48 h (Figure 2A,B and Figure S1D; *p* < 0.05). These results suggest that wt-BRG1 influences EGFR expression. Notably, in A549 cells, both siEGFR and gefitinib treatments significantly decreased cell viability compared to DMSO and C-siRNA-treated cells (Figure 2B, lower panel; *p* < 0.05). In contrast, gefitinib treatment, but not siEGFR treatment, significantly reduced NCI-H358 cell viability compared to controls.

### 3.4. BRG1 and EGFR Are Inversely Correlated

To delve deeper into the interplay between BRG1 and EGFR, we treated NCI-H358 cells with recombinant human EGF (50 nM) and monitored the expression of BRG1 and EGFR. The EGF-induced increase in EGFR expression was noticeable as early as 30 min and persisted for up to 3 h (Figure 2C). The original Western can be found in Figure S6. Concurrently, a time-dependent decrease in BRG1 protein expression was observed, indicating an inverse relationship between BRG1 and EGFR. Further evidence supporting this inverse correlation between BRG1 and EGFR was obtained from a panel of NSCLC cell lines characterized by varying mutational status of the two proteins, as well as from immunostaining of treatment-naïve lung tumor tissue microarrays (TMAs). Negative correlations between EGFR and BRG1 were evident in 5 out of 6 NSCLC cell lines (Figure S1E) tested. Importantly, TMA staining confirmed the negative relationship between BRG1 and EGFR (Figure 2D). The original Western blots can be found in Figure S6. Altogether, our in vitro findings suggest an inverse correlation between BRG1 and EGFR, emphasizing the vital role of wt-BRG1 in modulating EGFR expression.

### 3.5. Response of wt-EGFR NSCLC Cells to EGFR-TKIs Requires wt-BRG1

**Figure 3:**
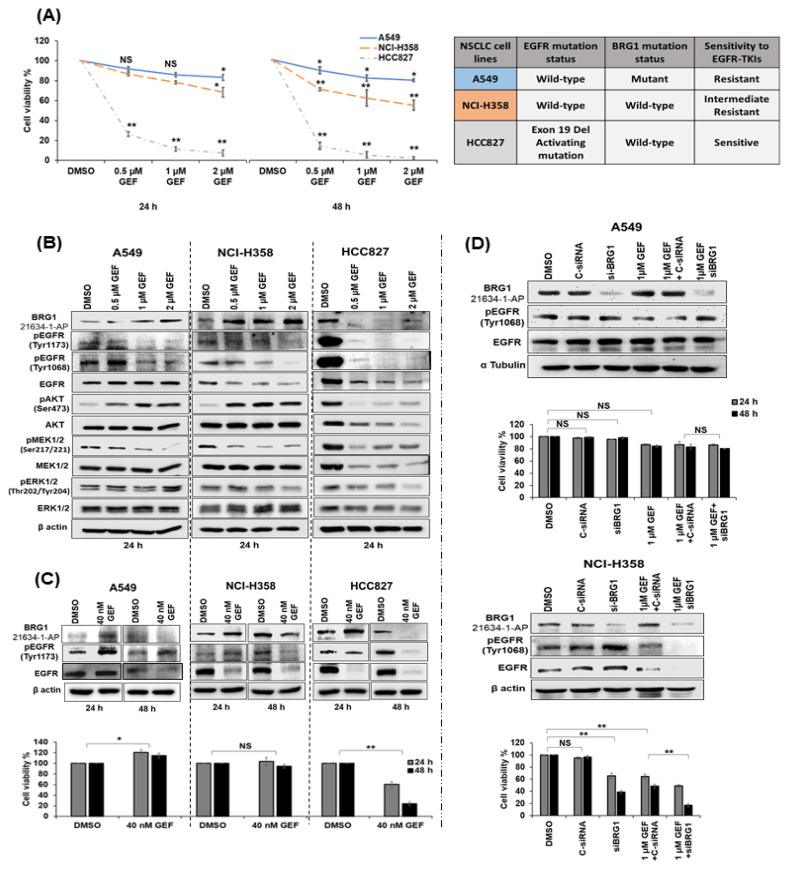
Evaluation of gefitinib cytotoxicity on NSCLC cell lines differing in their BRG1/EGFR status. (**A**) Gefitinib-treated cells (0.5 µM, 1 µM, and 2 µM GEF) were assessed for cell viability after 24 and 48 h. DMSO-treated cells served as controls. (**B**) Western blot analysis of BRG1 and EGFR-related proteins in NSCLC cell lines treated with gefitinib. Cells treated with DMSO or gefitinib were analyzed by Western blot, with β-actin used as a loading control. (**C**) Evaluation of cytotoxic effect and Western blot analysis in NSCLC cells treated with gefitinib (40 nM GEF) for 24 and 48 h. DMSO-treated cells served as controls, with β-actin used as a loading control. (**D**) Assessment of the inhibitory activity of siBRG1 (100 nM) and gefitinib (1 µM GEF) combination treatment on NSCLC cell lines. The cytotoxicity of siBRG1 and gefitinib treatment on A549 and NCI-H358 cells was determined at 24 and 48 h and analyzed for selected protein markers by Western blot analysis. Changes in viability and protein expression were compared to various control groups included in the study. α-Tubulin was used as a loading control. The original Western blot images of Figure 3B–D are provided in the Supplementary Figure S6. Error bars denote SD; NS = not significant; * *p* < 0.05; ** *p* < 0.01.

A549, NCI-H358, and HCC827 cells, each characterized by distinct EGFR and BRG1 status (Figure 3A), were treated with clinically relevant concentrations of gefitinib (0.5 µM, 1 µM, and 2 µM GEF). After 24 and 48 h of treatment, the cells were analyzed for drug sensitivity and for changes in molecular markers associated with the EGFR signaling pathway. HCC827 cells, harboring wt-BRG1 and mt-EGFR, exhibited the greatest sensitivity to gefitinib, with nearly complete cell death observed at 48 h (Figure 3A; *p* < 0.05). This heightened sensitivity in HCC827 cells was attributed to the presence of an activating EGFR mutation, consistent with previous findings [9]. In contrast, the sensitivity of NCI-H358 and A549 cells, both possessing wt-EGFR, varied considerably (Figure 3A), with A549 cells exhibiting greater resistance to gefitinib than NCI-H358 cells across all concentrations and time points.

Assessment for expression of proteins involved in the EGFR pathway following gefitinib treatment revealed a notable decrease in pEGFR^Tyr1173^ and pEGFR^Tyr1068^ levels, accompanied by increased BRG1 expression in both A549 and NCI-H358 cells at 24 h (Figure 3B and Figure S2A). The original Western blots can be found in Figure S6. However, a marked, dose-dependent decrease in total EGFR protein expression was observed in NCI-H358 but not in A549 cells (*p* < 0.05). Intriguingly, the reduction in total EGFR in NCI-H358 cells, even at the lowest gefitinib concentration (0.5 µM GEF), was comparable to that in HCC827 cells. Furthermore, proteins downstream of the EGFR pathway were differentially affected in gefitinib-treated A549 and NCI-H358 cells. While both cell lines exhibited varied reductions in pMEK1/2^Ser217/221^ and pERK^Thr202/Tyr204^ expression, the most notable observation was the increased expression of pAKT^Ser473^ in both (Figure 3B and Figure S2A). In HCC827, gefitinib treatment reduced the levels of BRG1, EGFR, and all the EGFR-related proteins, explaining its heightened sensitivity to gefitinib. At 48 h, the expression of these proteins in A549 and NCI-H358 cells was highly variable (Figure S2B,C). In NCI-H358 cells, pEGFR, EGFR, pMEK1/2^Ser217/221^, and pERK^Thr202/Tyr204^ showed reductions like those observed at 24 h, whereas in A549 cells, only pEGFR^Tyr1173^, pEGFR^Tyr1068^, and pMEK1/2^Ser217/221^ were reduced, with no change in total EGFR and pERK^Thr202/Tyr204^ expression. No significant increase in BRG1 expression was observed in either cell line following gefitinib treatment. In HCC827, reductions were observed in all the protein markers consistent with the observations at 24 h. These results underscore how the mutation status of BRG1 influences the response of wt-EGFR NSCLC cells to gefitinib.

Next, we investigated the effects of lower gefitinib concentrations on BRG1 and EGFR expressions and cell viability. Specifically, we selected 40 nM gefitinib, considering the established sensitivity of HCC827 cells to this concentration [77]. As illustrated in Figure 3C, gefitinib treatment significantly reduced the viability of HCC827 but had no notable effect on NCI-H358 cells. Interestingly, in A549 cells, we observed a significant increase in cell numbers, suggesting that the lower concentration of gefitinib may promote cell proliferation in mt-BRG1 cells. Correspondingly, pEGFR^Tyr1173^ protein levels increased in A549 cells at both 24 and 48 h after gefitinib treatment. Total EGFR was reduced at 48 h but not at 24 h in gefitinib treated A549 cells compared to DMSO treated cells. In NCI-H358, pEGFR^Tyr1173^ levels increased at 24 h but not at 48 h while total EGFR expression was reduced at both time points (Figure 3C and Figure S2D). The uncropped original Western blots of can be found in Figure S6. In HCC827, both pEGFR^Tyr1173^ and total EGFR protein expression were reduced at both time points, indicating the cell line’s sensitivity to the lower gefitinib concentration. Across all three cell lines, BRG1 was upregulated at 24 h and decreased at 48 h compared to DMSO-treated controls

Finally, we examined the influence of BRG1 on EGFR-TKI sensitivity beyond the first-generation TKI gefitinib, using the third-generation EGFR-TKI osimertinib (OSI). A549 and NCI-H358 cells were treated with osimertinib at concentrations of 15 nM and 500 nM, and cell viability along with changes in BRG1 and EGFR-associated molecular markers were assessed. Interestingly, NCI-H358 cells, but not A549 cells, displayed dose and time-dependent heightened sensitivity to osimertinib and marked changes in BRG1 and EGFR expression like those observed with gefitinib treatment (Figure S2E).

Previous studies [78,79] have highlighted the involvement of other RTKs in the response to EGFR inhibitors in cancer, including human epidermal growth factor receptor 2 (HER2), human epidermal growth factor receptor 3 (HER3), and MET (also known as hepatocyte growth factor receptor or HGFR). Thus, we examined the expression of these receptors in A549 and NCI-H358 cells treated with osimertinib. In A549 cells, osimertinib treatment decreased expression of HER2 with concomitant increase in Her3 and MET expression. In contrast, Her2, Her3 and MET protein expression were reduced in osimertinib-treated H358 cells (Figure S2F). Intriguingly, the expression patterns of HER3 and MET in osimertinib-treated cells mirrored the expression pattern of EGFR in gefitinib-treated A549 and NCI-H358 cells. These findings demonstrate that the BRG1 status influences the response of wt-EGFR NSCLC to EGFR-TKIs, irrespective of the specific EGFR-TKIs employed.

### 3.6. Genetic Inhibition of wt-BRG1 Sensitizes NSCLC Cells to Gefitinib

Independent studies [80,81], along with our observations herein, highlight the survival dependency of A549 and NCI-H358 cells on EGFR and BRG1, respectively. In NCI-H358 cells, wt-BRG1 predominates over wt-EGFR in providing survival signals. In contrast, in A549 cells, impairment of BRG1 function due to mutation of the BRD impairs its function and leads to its downregulation, resulting in EGFR governing the survival of cancer cells. This previously unexplored concept for improving EGFR-TKI efficacy was investigated in this study using A549 and NCI-H358 cells.

A549 and NCI-H358 cells were treated with siBRG1 (100 nM) for 24 h, followed by gefitinib (1 µM GEF), and compared to cells treated with either agent alone or with C-siRNA (100 nM), C-siRNA and gefitinib, or DMSO. Cells were harvested at 24 and 48 h after gefitinib treatment and subjected to cell viability and molecular analysis. As illustrated in Figure 3D and Figure S2G, the combination of siBRG1 and gefitinib treatment significantly reduced BRG1, pEGFR^Tyr1068^, and total EGFR in NCI-H358 cells, accompanied by a notable decrease in cell viability (*p* < 0.01). The original Western blots can be found in Figure S6. Conversely, in A549 cells, the siBRG1 and gefitinib combination did not significantly reduce cell viability compared to other treatments. The lack of cytotoxicity induced by the combination treatment in A549 cells can be attributed to the inability to reduce pEGFR^Tyr1068^ or total EGFR levels, despite the reduced BRG1 expression.

In contrast to the increased cytotoxicity observed with siBRG1/gefitinib treatment in NCI-H358 cells, combination treatment with PFI-3 (10 µM) and gefitinib (1µM) did not significantly affect cytotoxicity compared to individual treatments in NCI-H358 (Figure S2H). A plausible explanation for this observation is that PFI-3 does not directly decrease BRG1 expression but rather binds to the BRD region of BRG1, thereby disrupting its activity. Consequently, as expected, the combination treatment failed to produce enhanced antitumor activity. Nevertheless, our data reveals a compensatory mechanism wherein tumor cell survival is dependent on either EGFR or BRG1.

### 3.7. Genetic Modulation of BRG1 Negatively Regulates wt-EGFR and Enhances NSCLC Cell Growth

Prior reports on the compensatory functional relationship [82,83] between two crucial core subunits, BRG1 and BRM, within the SWI/SNF complex, led us to investigate the effect on BRM expression upon BRG1 overexpression in A549 cells and on BRG1 silencing in NCI-H358 cells. We generated stable BRG1-overexpressing A549 cells (A549-BRG1) and stable BRG1 knock-out NCI-H358 cells (H358-BRG1-KO). Effective knock-out of BRG1 in NCI-H538 cells was confirmed in three individual clones (1, 2, and 3) by DNA sequencing (Figure S3A). Subsequently, cells were analyzed for cell proliferation and for expression of BRM and molecular markers downstream of the BRG1/BRM pathway (Figure S3B,C). Western blot analysis revealed that BRM expression remained unaffected in our A549-BRG1 and in the three H358-BRG1-KO clones (1, 2, and 3) compared to control cells, including A549-Puro, parental A549, H358-Scramble (H358-Scr), and parental NCI-H358 cells. However, notable changes were observed in the expression levels of pEGFR^Tyr1068^, total EGFR, pAKT^Ser473^, pERK1/2^Thr202/Tyr204^, and total ERK1/2 in A549-BRG1 cells compared to A549-Puro and A549 parental cells, while AKT and EGF expression remained unaffected. Conversely, all three H358-BRG1-KO clones exhibited increased expression levels of pEGFR^Tyr1068^, EGFR, pAKT^Ser473^, pERK1/2^Thr202/Tyr204^, ERK1/2, and EGF compared to H358-Scr and parental NCI-H358 cells, with no alteration in AKT expression (Figure S3C). Cell viability assays supported these observations, demonstrating that both BRG1 overexpression and knock-out significantly enhanced cell viability compared to the respective parental cell lines and were independent of BRM (Figure S3B).

### 3.8. Overexpression of BRG1 Sensitizes NSCLC Cells to Gefitinib Both In Vitro and In Vivo

**Figure 4:**
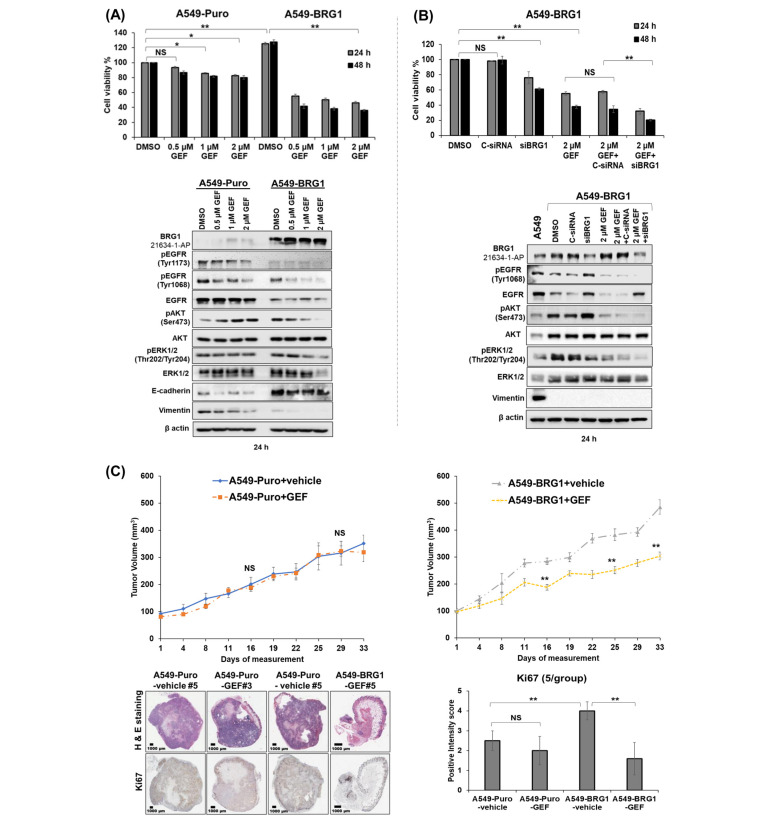

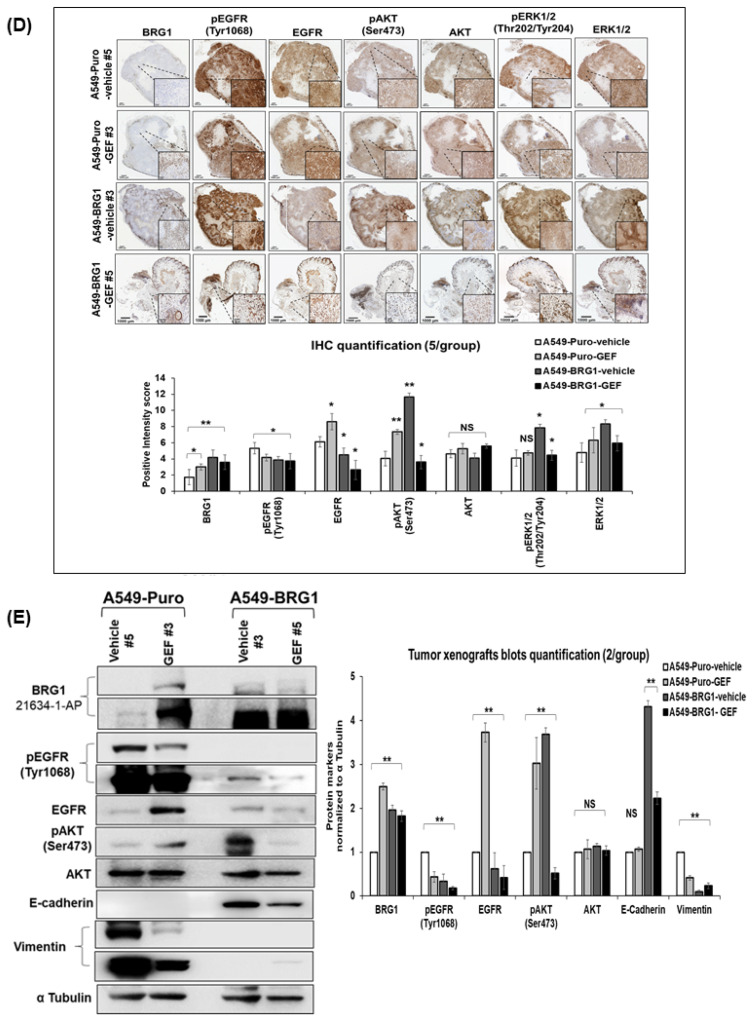
Effect of gefitinib on A549-Puro and A549-BRG1 cells and its impact on the EGFR signaling pathway in vitro and in vivo. (**A**) The in vitro cytotoxic effect of gefitinib (0.5–2 µM GEF) treatment on A549-Puro and A549-BRG1 cells at 24 and 48 h (upper panel). DMSO-treated cells served as controls. Western blot analysis of selected markers in gefitinib-treated A549-Puro and A549-BRG1 cells at 24 h (lower panel). (**B**) The cytotoxic effects of siBRG1 (100 nM) in combination with gefitinib (2 µM) on A549-BRG1 cells at 24 and 48 h (upper panel). Western blot analysis of selected markers following siBRG1 and gefitinib combination treatment in A549-BRG1 cells (lower panel). DMSO and C-siRNA (100 nM)-treated cells served as controls. Parental A549 cells were included as control for comparing BRG1 overexpression. β-Actin was used as loading control in Western blotting. (**C**) Tumor growth curves in nude mice bearing A549-Puro and A549-BRG1 tumor xenografts treated with either DMSO (vehicle) or gefitinib (100 mg/kg) are illustrated in the upper panel. Representative H&E and Ki67 stained tumor tissues are displayed at 10 X magnification: Scale bar, 1000 µm. Quantitative analysis for Ki67 staining is presented as a bar graph. (**D**) Gefitinib-treated A549-Puro and A549-BRG1 tumor tissues were immunostained for selected proteins and compared to DMSO-treated tumors. Differences in protein expression were quantified and presented as bar graphs. Magnification, 10 X; Scale bar, 1000 µm. (**E**) Western blot analysis of selected markers was performed in A549-Puro and A549-BRG1 tumor xenografts treated with DMSO (vehicle) or gefitinib (100 mg/kg). A representative blot showing changes in the protein expression levels is shown. Changes in the protein expression levels in two separate sets of tumor samples for each treatment and tumor group were quantified and presented as bar graphs. α-Tubulin was used as loading control. All original Western blot images are provided in the Supplementary Figure S6. Error bars denote SD; NS = not significant; * *p* < 0.05; ** *p* < 0.01.

To investigate how BRG1 overexpression influences the response to EGFR-TKI, A549-Puro and A549-BRG1 cells were treated with gefitinib at varying concentrations (0.5 µM, 1 µM, 2 µM GEF), and their cell viability was assessed. The results depicted in Figure 4A unveiled a notable reduction in A549-BRG1 cell viability compared to A549-Puro cells across all tested concentrations and time points (*p* < 0.01). Molecular analysis revealed that BRG1 overexpression in A549-BRG1 cells correlated with increased levels of pAKT^Ser473^ and pERK1/2^Thr202/Tyr204^ compared to A549-Puro cells (Figure 4A and Figure S3D). The original Western blots can be found in Figure S6.

However, gefitinib treatment, while augmenting BRG1 expression in A549-BRG1 cells, simultaneously led to a decrease in levels of pEGFR^Tyr1173^, pEGFR^Tyr1068^, and total EGFR, across all concentrations tested. Conversely, the inhibitory effect of gefitinib on pAKT^Ser473^ was noticeable at 1 µM and 2 µM, while suppression of pERK1/2^Thr202/Tyr204^ expression was observed at 2 µM. In A549-Puro cells, gefitinib treatment increased BRG1 expression compared to DMSO-treated controls, albeit significantly less than that observed in A549-BRG1 cells (Figure 4A and Figure S3D). Additionally, gefitinib at higher concentrations (1 µM and 2 µM) reduced pEGFR^Tyr1068^ and pEGFR^Tyr1173^ and had no significant effect on total EGFR and pERK1/2^Thr202/Tyr204^. Notably, pAKT^Ser473^ expression increased in a dose-dependent manner with increasing concentration of gefitinib (Figure 4A and Figure S3D). Intriguingly, in vitro findings revealed that BRG1 overexpression in A549-BRG1 cells led to a significant increase in E-cadherin expression and a significant reduction in vimentin compared to their levels in A549-Puro cells. However, gefitinib treatment resulted in a greater reduction in both E-cadherin and vimentin expression in A549-Puro cells compared to A549-BRG1 cells. In a separate experiment, A549-BRG1 cells were treated with siBRG1 (100 nM) or C-siRNA (100 nM), followed by gefitinib (2 µM GEF) and compared to each individual treatment. Cell viability assessments showed that the combination treatment elicited superior inhibitory effects compared to C-siRNA plus gefitinib or the individual treatments at both 24 and 48 h (Figure 4B). Consequently, all protein markers except for EGFR and AKT were evaluated, including BRG1, pEGFR^Tyr1068^, pAKT^Ser473^, pERK1/2^Thr202/Tyr204^, and ERK1/2, and exhibited reduced expression levels upon combination treatment (Figure 4B and Figure S3E). The original Western blots associated can be found in Figure S6. These findings not only corroborate our previous findings in NCI-H358 cells treated with siBRG1 and gefitinib combination (Figure 3D) but also highlight the importance of wt-BRG1 in cell survival, advocating for the development of potent BRG1 inhibitors (Figure 4B and Figure S3E). The original Western blots can be found in Figure S6.

To validate our in vitro observations regarding the impact of BRG1 on gefitinib sensitivity, we conducted in vivo studies using mice bearing subcutaneous tumors derived from A549-Puro and A549-BRG1 cells. Treatment with gefitinib (100 mg/kg) markedly delayed the growth of A549-BRG1 tumors compared to controls (Figure 4C; right panel; *p* < 0.01) but did not notably limit the growth of A549-Puro tumors (Figure 4C; left panel). Immunohistochemical (IHC) analysis revealed that gefitinib treatment significantly reduced Ki67 expression and induced necrosis in A549-BRG1 tumors compared to A549-Puro tumors and DMSO-treated controls (Figure 4C; *p* < 0.01). Furthermore, the expression levels of pEGFR^Tyr1068^, EGFR, pAKT^Ser473^, and pERK1/2^Thr202/Tyr204^ were significantly reduced in gefitinib-treated A549-BRG1 tumors compared to A549-Puro tumors and DMSO-treated controls (Figure 4D; *p* < 0.05). Subsequently, Western blot analysis of tumor tissue lysates from A549-BRG1 tumors revealed reduced pEGFR^Tyr1068^, EGFR, and vimentin expression, which was further attenuated following gefitinib treatment (Figure 4E, *p* < 0.05), relative to their expression levels in gefitinib- and DMSO-treated A549-Puro tumors. Conversely, an increase in pAKT^Ser473^ and E-cadherin expression was observed in A549-BRG1 tumors, which was diminished upon gefitinib treatment. In A549-Puro tumors, gefitinib treatment notably elevated pAKT^Ser473^ expression compared to DMSO-treated controls, consistent with our in vitro findings. E-cadherin expression was undetectable in A549-Puro cells. Uncropped Western blot images used in Figure 4E are provided in Figure S6. In summary, our in vivo findings corroborate the in vitro results and underscore that BRG1 overexpression in mt-BRG1 A549 cells enhances their sensitivity to gefitinib.

### 3.9. BRG1 Knock-Out Enhances the Resistance of NSCLC Cells to Gefitinib Both In Vitro and In Vivo

**Figure 5:**
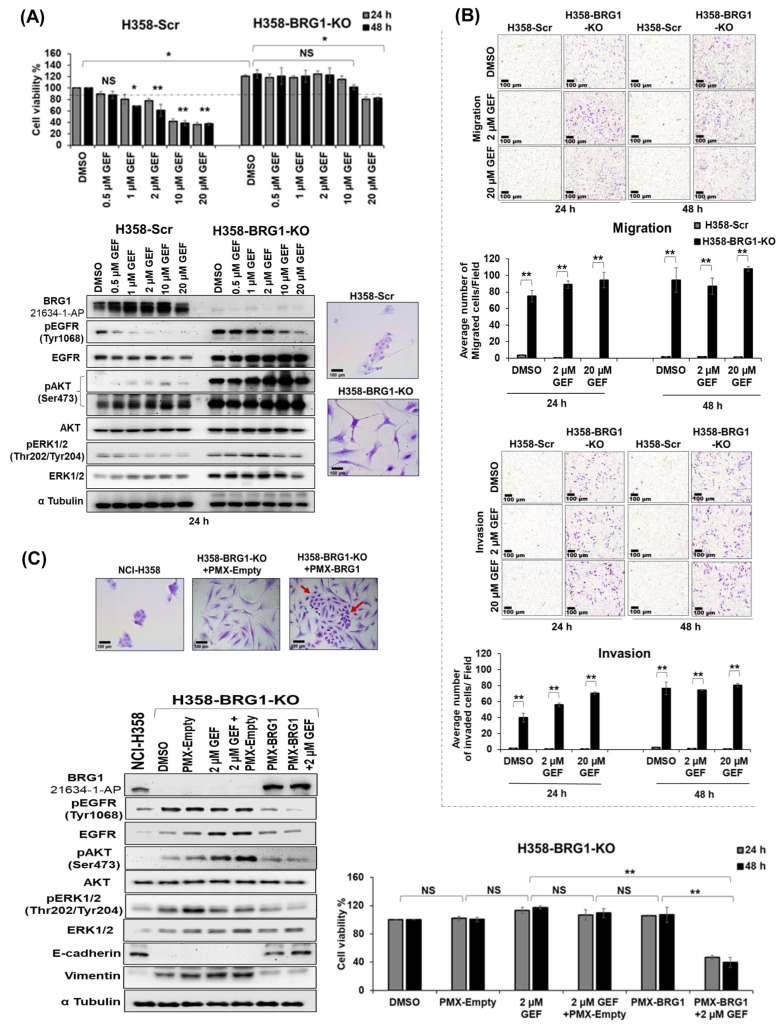

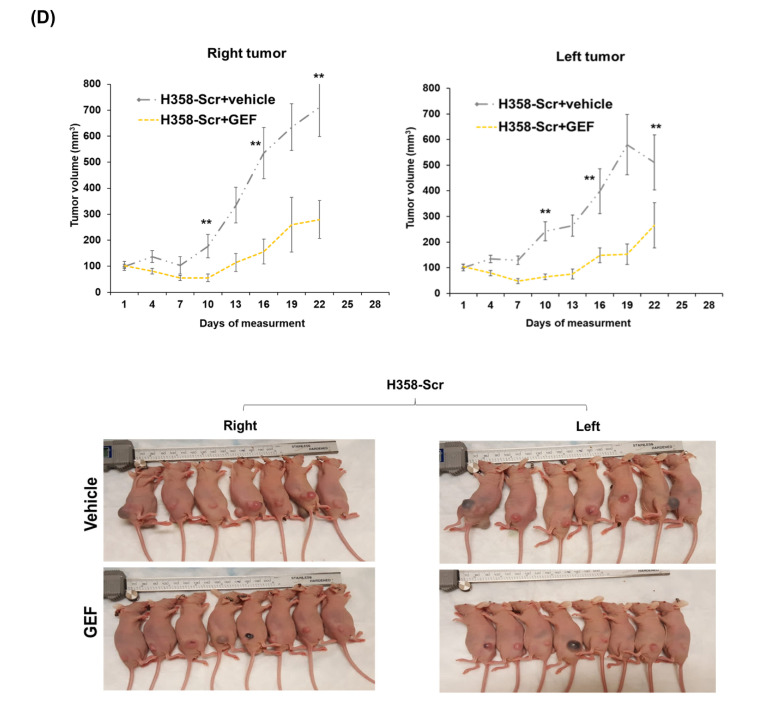
Effect of gefitinib on H358-Scr and H358-BRG1-KO cells and its influence on the EGFR signaling pathway both in vitro and in vivo. (**A**) The effect of gefitinib treatment on cell viability and signaling pathways in H358-Scr and H358-BRG1-KO cells. Cells treated with varying concentrations of gefitinib (0.5–20 µM GEF) were assessed for cell viability, morphological changes, and expression levels of selected markers by Western blot analysis. DMSO-treated cells served as controls. Cell viability is depicted in a bar graph for 24 and 48 h post-treatment. Representative images of crystal violet-stained cells are shown at 20X magnification; Scale bar, 100 µm. α-Tubulin was used as a loading control for Western blotting. (**B**) H358-Scr and H358-BRG1-KO cells were treated with either DMSO or gefitinib (2 µM and 20 µM GEF) and the average number of migrated (upper panel) and invaded (lower panel) cells was determined at 24 and 48 h after treatment. Representative images of migrated and invaded cells are shown at 10X magnification; Scale bar, 100 µm. (**C**) Western blot analysis of selected markers at 24 h following PMX-BRG1 and gefitinib combination treatment of H358-BRG1-KO cells in vitro. DMSO, PMX-empty, and a combination of PMX-empty and gefitinib-treated cells were used as controls. NCI-H358 cells were utilized for comparison to BRG1-KO cells. α-Tubulin was used as loading control. Representative field pictures of crystal violet-stained cells are shown at 20X magnification; Scale bar, 100 µm. Cell viability is shown in the bar graph for 24 h and 48 h post-PMX-BRG1 + GEF treatment. (**D**) Tumor growth curve in nude mice bearing contralateral H358-Scr tumor xenografts treated with either DMSO (vehicle) or gefitinib (100 mg/kg). Representative images of mice bearing H358-Scr tumors from the vehicle or gefitinib treatment are displayed. All original Western blot images are provided in the Supplementary Figure S6. Error bars denote SD; NS = not significant; * *p* < 0.05; ** *p* < 0.01.

To assess the impact of BRG1 depletion on gefitinib response, one H358-BRG1-KO clone was treated with varying concentrations of gefitinib (0.5 µM, 1 µM, 2 µM, 10 µM, and 20 µM) and its response was compared to H358-Scr cells, with DMSO-treated cells serving as the vehicle control. Morphological examination revealed a distinct phenotype in H358-BRG1-KO cells, displaying a disassociated and mesenchymal appearance compared to the tightly held and compacted morphology of H358-Scr cells. This suggests that BRG1 loss potentially leads to a transition from the epithelial-to-mesenchymal phenotype (Figure 5A) and concurs with previous reports [84,85]. Cell viability studies showed H358-BRG1-KO cells to be resistant to gefitinib across all concentrations except for 20 µM at both 24 and 48 h (Figure 5A). The observed 20% inhibitory activity with 20 µM gefitinib treatment in H358-BRG1-KO cells was comparable to the inhibitory activity observed in H358-Scr cells with 1.0 µM gefitinib. These findings indicate that BRG1 loss results in EGFR-TKI resistance, with an approximately 20-times higher gefitinib dose required to achieve about 20% cell killing. Molecular analysis showed an increased baseline expression of pEGFR^Tyr1068^, EGFR, pAKT^Ser473^, pERK1/2^Thr202/Tyr204^, and ERK1/2 in BRG1-KO cells compared to BRG1-Scr cells, which were further increased upon gefitinib treatment (0.5 µM–2 µM GEF). Notably, significant inhibition of pEGFR^Tyr1068^ and pERK1/2^Thr202/Tyr204^ was observed only at 10 µM and 20 µM gefitinib concentrations (Figure 5A and Figure S3F; *p* < 0.05). In contrast, gefitinib treatment in H358-Scr cells induced BRG1, pAKT^Ser473^, and ERK1/2 expression while decreasing pEGFR^Tyr1068^, EGFR, and pERK1/2^Thr202/Tyr204^ in a dose-dependent manner (Figure 5A and Figure S3F; *p* < 0.05). The original Western blots can be found in Figure S6. 

Given the altered morphology of NCI-H358 cells due to BRG1-KO, we evaluated the migratory and invasive capabilities of H358-BRG1-KO cells. Remarkably, H358-BRG1-KO cells exhibited significantly increased migratory and invasive properties compared to H358-Scr cells (Figure 5B; *p* < 0.01). Treatment with gefitinib (2 µM and 20 µM GEF) failed to attenuate these migratory and invasive properties of H358-BRG1-KO cells. In contrast, negligible migration and invasion were observed in H358-Scr cells treated with either DMSO or gefitinib (2 µM or 20 µM GEF). The ability of the BRG1-KO cells to have enhanced migratory and invasive properties is in agreement with the EMT phenotype.

Our findings thus far suggest that BRG1 loss contributes to gefitinib resistance in NSCLC cells, prompting the question of whether restoring BRG1 expression could restore sensitivity. Reintroduction of BRG1 into H358-BRG1-KO cells using the PMX-BRG1 expression plasmid resulted in significant sensitivity to gefitinib (2 µM GEF) at both 24 and 48 h post-treatment, whereas cells transfected with an empty vector (PMX-empty) showed no response towards gefitinib (2 µM GEF; Figure 5C; *p* < 0.01). Moreover, restoring BRG1 expression reverted the morphology of H358-BRG1-KO cells to resemble parental NCI-H358 cells. Western blot analysis of cell lysates from both PMX-BRG1-transfected cells and gefitinib (2 µM GEF)-treated H358-BRG1-KO-PMX-BRG1 cells showed a significant reduction in the expression of pEGFR^Tyr1068^, EGFR, pAKT^Ser473^, and pERK1/2^Thr202/Tyr204^ compared to PMX-empty transfected cells and gefitinib-treated H358-BRG1-KO-PMX-empty cells. No significant changes in AKT and ERK1/2 expression were observed following gefitinib treatment in either PMX-empty or PMX-BRG1 transfected cells. Furthermore, E-cadherin and vimentin expression, known targets of BRG1 and contributors to the EMT phenotype, were modulated, with increased expression of E-cadherin and a concomitant reduction in vimentin in gefitinib-treated PMX-BRG1 cells and PMX-BRG1 cells (Figure 5C and Figure S4A; *p* < 0.01). The original Western blots can be found in Figure S6. 

These studies were further corroborated in vivo, using subcutaneous tumor xenografts established from H358-Scr and H358-BRG1-KO cells. Mice bearing H358-Scr tumors on contralateral flanks exhibited a significant delay in tumor growth upon intraperitoneal gefitinib treatment (100 mg/kg) compared to DMSO (vehicle)-treated tumors (Figure 5D; *p* < 0.01). Unlike H358-Scr cells’ ability to form tumors in mice, implantation of H358-BRG1-KO cells in mice resulted in very small tumors that grew at a significantly slower rate over a 29-day period. Additionally, the tumors became necrotic at a size smaller than 100mm^3^, thus hindering our ability to test the efficacy of gefitinib (Figure S4B).

### 3.10. BRG1 Plays a Role in the Resistance to EGFR-TKIs by Promoting the Formation of the EGFR–pAKT^Ser473^ Complex

**Figure 6:**
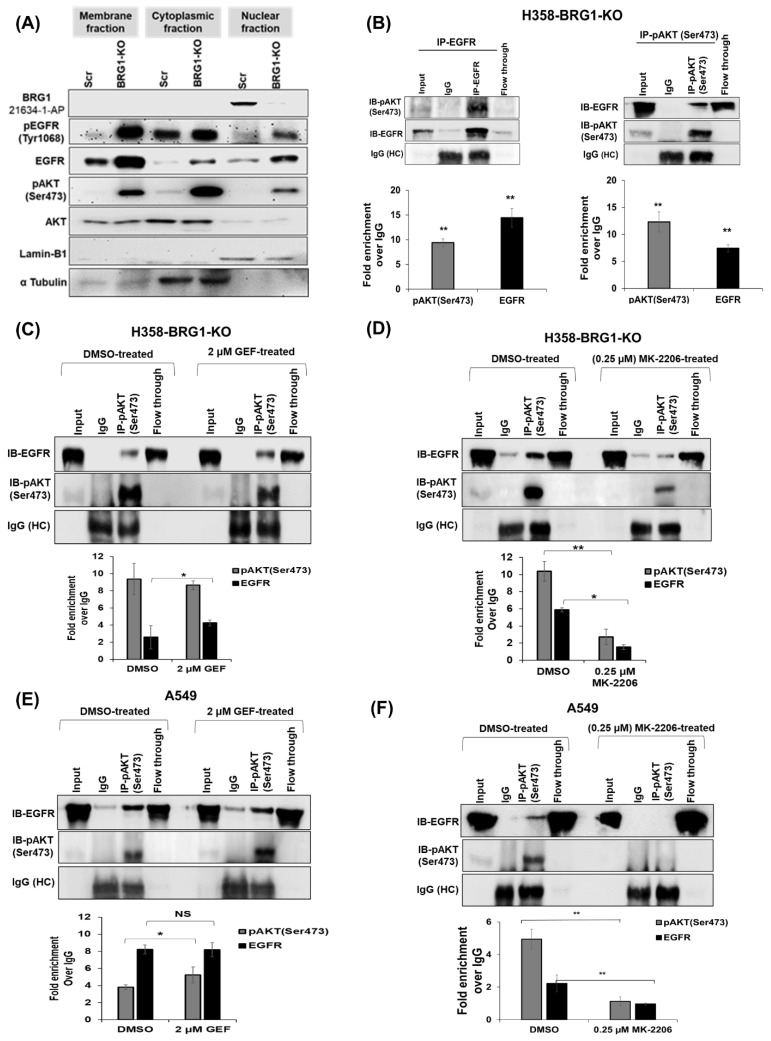

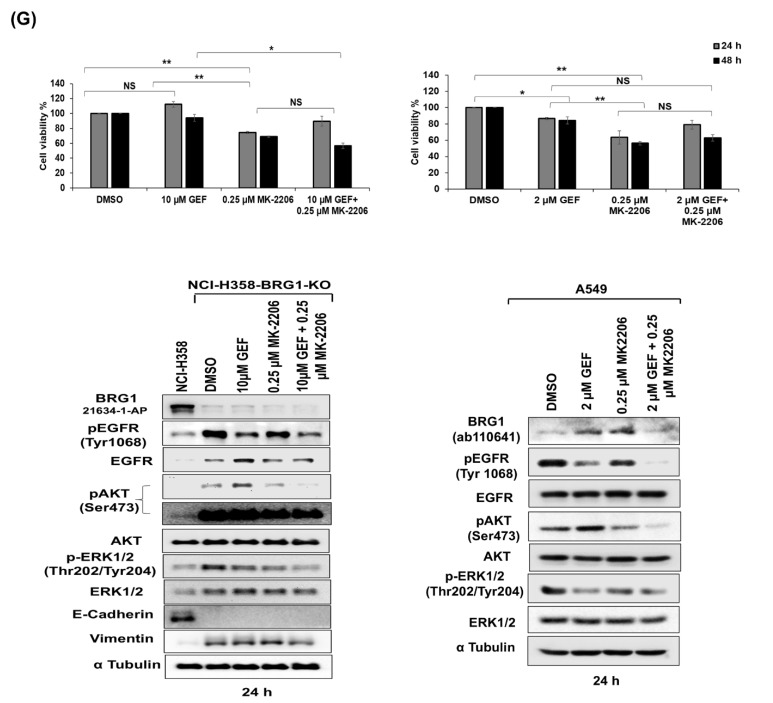
BRG1 mutation status governs the wt-EGFR–pAKT^Ser473^ complex formation. (**A**) Subcellular fractionation assays revealed a notable increase in pEGFR^Tyr1068^, EGFR, and pAKT^Ser473^ levels in the membrane, cytoplasmic, and nuclear fractions of H358-BRG1-KO compared to H358-Scr cells with no significant change observed in AKT protein levels. Lamin-B1 and α-tubulin served as loading controls. (**B**) Co-immunoprecipitation and Western blot analysis confirms the physical interaction between EGFR and pAKT^Ser47^, forming the EGFR–pAKT^Ser473^ complex in H358-BRG1-KO cells. Semi-quantitative analysis of the Western blot demonstrated the fold enrichment of EGFR and pAKT^Ser473^ over IgG control, as shown in bar graphs. IgG heavy chain (HC) is used as a loading control. (**C**,**E**) Immunoprecipitation and Western blot analysis of pAKT^Ser473^ and associated EGFR revealed that gefitinib (2 µM) treatment did not disrupt the EGFR–pAKT^Ser473^ interaction. (**D**,**F**) A minimal concentration of MK-2206 (0.25 µM) significantly reduced the enrichment of pAKT^Ser473^ and associated EGFR in both mt-BRG1 H358-BRG1-KO and A549 cells. DMSO-treated cells were used as controls. Fold enrichment of EGFR and pAKT^Ser473^ over IgG heavy chain (HC) was determined by semi-quantitative analysis of the Western blot and shown as bar graphs. (**G**) The cytotoxic effect of gefitinib (2 µM GEF), MK-2206 (0.25 µM), and the combination of gefitinib and MK-2206 was assessed by cell viability and Western blotting for cell signaling markers in H358-BRG1-KO and A549 cells. Cells treated with gefitinib, MK-2206, or the combination of gefitinib and MK-2206 were analyzed for cell viability at 24 and 48 h and for selected markers by Western blot analysis at 24 h. Cell viability is shown as a bar graph. DMSO-treated cells were used as controls. α-Tubulin was used as a loading control. The original Western blot images are provided in the Supplementary Figure S6. Error bars denote SD; NS = not significant; * *p* < 0.05; ** *p* < 0.01.

Previous studies have linked the activation of EGFR and AKT signaling pathways following treatment with various targeted therapies to therapy resistance [26,27,28,29]. Moreover, the physical interaction between wt-EGFR and AKT has been implicated in gefitinib resistance in breast cancer [86,87,88,89]. Drawing from these prior reports and our own findings prompted us to explore whether cells with mutant BRG1 benefit from increased expression of EGFR and pAKT^Ser473^ to resist EGFR-TKIs. We examined changes in EGFR and pAKT^Ser473^ protein expression in A549 cells that harbor mt-BRG1 and in H358-BRG1-KO cells following EGFR-TKI treatment, aiming to understand the contribution of EGFR–pAKT^Ser473^ complex formation in gefitinib resistance. Initially, we analyzed EGFR and pAKT^Ser473^ expression and localization in H358-BRG1-KO cells compared to H358-Scr cells through cell fractionation studies. Loss of BRG1 (BRG1-KO) significantly increased the levels of pEGFR^Tyr1068^ and total EGFR proteins in the nuclear, cytoplasmic, and membrane fractions of H358-BRG1-KO cells compared to H358-Scr cells (Figure 6A). The original Western blots can be found in Figure S6. Flow cytometry analysis confirmed these findings (Figure S4C; *p* < 0.01). Alongside increased levels of pEGFR^Tyr1068^ and total EGFR, there was a notable increase in pAKT^Ser473^ levels across all three cellular fractions in the H358-BRG1-KO cells (Figure 6A). However, AKT expression remained unchanged in the cellular fractions of H358-BRG1-KO and H358-Scr cells.

We next examined the physical interaction between EGFR and pAKT^Ser47^ in H358-BRG1-KO cells using an immunoprecipitation assay combined with an antibody pull-down assay. A strong interaction between EGFR and pAKT^Ser47^ was observed in H358-BRG1-KO cells (Figure 6B), supporting their potential role in gefitinib resistance. In comparison, treatment with gefitinib (2 µM GEF) did not affect the EGFR–pAKT^Ser473^ interaction in H358-BRG1-KO cells (Figure 6C). However, treatment with an AKT inhibitor (MK-2206; 0.25 µM) significantly reduced pAKT^Ser473^ and associated EGFR levels compared to DMSO-treated cells (Figure 6D; *p* < 0.05). In A549 cells, no significant increase in levels of EGFR bound to pAKT^Ser437^ was observed following treatment with gefitinib (2 µM), although pAKT^Ser437^ levels increased compared to DMSO-treated cells (Figure 6E; *p* < 0.05). Treatment with MK-2206 completely abrogated the EGFR and pAKT^Ser473^ interaction, as evidenced by undetectable levels of the two proteins compared to DMSO-treated control (Figure 6F; *p* < 0.05). Collectively, these results indicate that EGFR, in part, mediated resistance to EGFR-TKIs. The complete original Western blots can be found in Figure S6.

To further investigate whether the EGFR and pAKT^Ser473^ interaction observed in vitro also occurs in vivo, A549-Puro and A549-BRG1 tumor tissues treated with DMSO or gefitinib were stained for EGFR and pAKT^Ser473^ complex formation, and positivity in different cellular compartments (membrane, cytoplasmic, and nuclear) of stained tissues was assessed and quantified. In A549-Puro tumors, gefitinib treatment led to increased staining intensity of EGFR and pAKT^Ser473^ in the membrane and cytoplasmic fractions but reduced staining in the nuclear fraction (Figure S4D; *p* < 0.05). Conversely, gefitinib-sensitive A549-BRG1 tumors exhibited reduced staining for both proteins across all cellular compartments following treatment (Figure S4D; *p* < 0.01). A color-coded annotation of EGFR and pAKT^Ser473^ staining patterns of cells in tumor tissue is shown in Figure S4E. Our data collectively demonstrates that EGFR/pAKT^Ser473^ interaction likely contributes to gefitinib resistance, and disruption of the protein interaction will likely improve treatment outcomes.

To determine whether targeting AKT and disrupting the interaction between EGFR and pAKT^Ser473^ could enhance gefitinib efficacy in lung cancer cells with mt-BRG1 and wt-EGFR status, H358-BRG1-KO and parental A549 (mt-BRG1) cells were treated with gefitinib alone (2 µM or 10 µM GEF), MK2206 (0.25 µM) alone, or the combination of both drugs. In H358-BRG1-KO cells, treatment with 10 µM gefitinib alone did not induce significant cytotoxicity, while MK-2206 treatment (0.25 µM) exhibited notable cytotoxicity, which was not potentiated by combining with gefitinib (Figure 6G; *p* < 0.01). In A549 cells, gefitinib treatment (2 µM GEF) alone demonstrated significant cytotoxicity (Figure 6G; *p* < 0.05); MK-2206 treatment (0.25 µM; *p* < 0.01) was still more cytotoxic, and the combination treatment, as in H358-BRG1-KO cells, did not enhance cytotoxicity. These findings were corroborated by molecular changes observed in both cell lines. In both A549 cells and H358-BRG1-KO, gefitinib treatment significantly reduced pEGFR^Tyr1068^ and pERK1/2^Thr202/Tyr204^ while increasing pAKT^Ser473^ without affecting EGFR and AKT expression (Figure 6G and Figure S4F,G). MK-2206 treatment selectively and significantly decreased pAKT^Ser473^ and pERK1/2^Thr202/Tyr204^ but did not influence pEGFR^Tyr1068^, EGFR, or AKT in either cell line. However, the combination of gefitinib and MK-2206 treatment showed enhanced inhibitory effects on pAKT^Ser473^ and pERK1/2 levels in both cell lines. The only disparity in the combination treatment effect between the two cell lines was the observed reduction of pEGFR^Tyr1068^ in A549 cells but not in H358-BRG1-KO cells (Figure 6G and Figure S4F,G). The original Western blots are presented in Figure S6.

Subsequently, we investigated whether the EGFR–pAKT complex formation and subsequent sensitivity to AKT inhibition could also contribute to osimertinib resistance. First, the efficacy of osimertinib was determined in parental A549 cells by treatment with osimertinib alone (500 nM), MK-2206 treatment alone (0.25 µM), or their combination. Cells treated with osimertinib showed a reduction in cell viability, albeit not statistically significant when compared to DMSO-treated cells at 24 h (Figure S5A). In contrast, MK-2206-treated cells showed a significant reduction in cell viability compared to osimertinib-treated and DMSO-treated cells (*p* < 0.01). Combination therapy did not show any significant enhancement in cytotoxicity when compared to MK-2206 alone. At 48 h, individual treatment with osimertinib and MK-2206 showed a significant reduction compared to DMSO control. However, combination treatment showed no significant cytotoxicity when compared to individual treatments. Next, A549 cells treated with osimertinib (500 nM) were subjected to immunoprecipitation combined with an antibody pull-down assay, as described for gefitinib treatment. Increased levels of EGFR bound to pAKT^Ser47^ were observed in osimertinib-treated A549 cells compared to DMSO-treated cells (Figure S5B). There was no marked increase in the pAKT^Ser47^ levels in osimertinib-treated cells compared to control cells. These results mimic the observations made in gefitinib-treated H358-BRG1-KO cells and A549 cells (Figure 6B). These results demonstrate that the EGFR–pAKT complex formation and subsequent sensitivity to AKT inhibition hold true not only for gefitinib but also for the third-generation EGFR-TKI, osimertinib.

## 4. Discussion

In this study, we conducted experiments to understand the role of BRG1 and its influence on EGFR expression and response to EGFR TKIs. NSCLC cell lines possessing wt-EGFR but varying BRG1 mutational status were used in both in vitro and in vivo studies. In vitro experiments revealed HCC827 (wt-BRG/mt-EGFR) was highly sensitive to gefitinib treatment. In contrast, NCI-H358 cells (with wt-BRG1/wt-EGFR) showed a modest response, and A549 cells (mt-BRG1/wt-EGFR) exhibited reduced responsiveness to gefitinib treatment at equimolar drug concentrations. Genetic analysis of major oncogenic drivers (K-Ras, N-Ras, PIK3CA, BRAF, LKB1, ALK, Her2, and CDKN2A) in these two cell lines did not appear to influence gefitinib response. Molecular analyses demonstrated that gefitinib treatment led to upregulated BRG1 while concurrently reducing pEGFR and total EGFR in NCI-H358 cells. Conversely, in gefitinib-treated A549 cells, the upregulation of BRG1 was accompanied by decreased levels of pEGFR but not total EGFR. This modulation of EGFR and BRG1 expression by gefitinib occurred at the transcriptional level, a novel finding that has not been previously reported. Employing both genetic and pharmacologic inhibitors targeting BRG1 resulted in increased expression of pEGFR and total EGFR in NCI-H358 cells, while no significant changes were observed in A549 cells. Conversely, overexpression of BRG1 decreased levels of pEGFR and total EGFR in both A549 and NCI-H358 cells. Genetic inhibition of EGFR led to increased BRG1 mRNA expression in both cell lines. In contrast, pharmacologic inhibition with gefitinib resulted in increased EGFR mRNA and protein levels in A549 cells but decreased levels in NCI-H358 cells. Moreover, BRG1 overexpression in A549 restored sensitivity to gefitinib, while BRG1 knock-out in NCI-H358 cells conferred resistance. In vivo studies confirmed these findings, demonstrating that BRG1 overexpression in A549 tumors enhanced gefitinib sensitivity and suppressed tumor growth, akin to the effects observed in gefitinib-treated NCI-H358 tumors. These results from both in vitro and in vivo studies convincingly illustrated an inverse correlation between BRG1 and EGFR, consistent with findings from our NSCLC TMA analysis. Furthermore, our studies revealed that BRG1 and EGFR exerted opposing effects, with BRG1 mutational status influencing a pro-survival signal and evasion of TKI cytotoxicity. One caveat in the present study is that the efficacy of gefitinib in NCI-H358-BRG1-KO tumors was not tested, as they grew slowly and became necrotic. One possible explanation for this observation is that the loss of BRG1 likely affects the structure and integrity of tumor vasculature, leading to hemorrhage. Loss of BRG1 has previously been shown to contribute to loss of vasculature integrity [90]. Another possibility is that the gain of a mesenchymal phenotype upon loss of BRG1 leads to an invasive phenotype and establishment of deep tissue tumors and distal metastasis. However, we did not find the presence of tumors in deep tissue underneath the tumor cell injection site, nor in any major organs (lungs, colon, kidney, or liver) during gross or histopathological examination. Additional studies are required to understand the failure of tumors to grow and is beyond the scope of the present study.

While evidence suggests a connection between BRG1 status, EGFR expression, and TKI resistance, the specific role of BRG1 mutation in contributing to EGFR resistance has remained elusive. Prior studies have implicated AKT, a pro-survival protein and downstream target of the EGFR signaling pathway, in EGFR-TKI resistance [26,27,28,29]. Additionally, there have been reports that BRG1 regulates AKT [29,91]. Consistent with these findings, we observed an increase in pAKT but not total AKT expression, along with elevated EGFR expression in gefitinib-resistant H358-BRG1-KO cells. This suggests a potential switch in survival signaling, with cells lacking functional BRG1 relying more on AKT than EGFR. Another plausible mechanism is that the loss of BRG1 enhances the interaction between AKT and EGFR, forming a complex that translocates to the nucleus, where EGFR may act as a transcription factor for cell survival genes, leading to TKI resistance [86,87,88,89]. Evidence for the formation of the pAKT^(Ser473)^–EGFR complex stemmed from cell fractionation studies, and confirmation of TKI resistance due to this complex was demonstrated through immunoprecipitation studies in H358-BRG1-KO cells and A549 cells. The addition of the AKT inhibitor, MK2206, to H358-BRG1-KO cells and A549 cells disrupted the complex formation and produced significant cytotoxicity, surpassing that produced by gefitinib treatment alone or in combination with MK2206. In contrast, the complex between pAKT^(Ser473)^ and EGFR remained intact in cells treated with gefitinib, elucidating the resistance mechanism to this drug. However, MK-2206 disrupts the interaction, rendering the cells sensitive to the treatment, indicating that the sensitivity of NSCLC cells relies on disrupting the pAKT^(Ser473)^–EGFR complex. Even though gefitinib treatment increased pAKT^(Ser473)^ levels in both A549 and NCI-H358 cells, sensitivity hinges on EGFR binding to pAKT^(Ser473)^. Treatments with gefitinib and osimertinib reduced EGFR expression in NCI-H358 cells with wt-BRG1, disrupting its interaction with pAKT^Ser473^ and resulting in sensitivity. Conversely, gefitinib treatment does not affect EGFR expression in A549 cells with mt-BRG1, maintaining the integrity of the pAKT^(Ser473)^–EGFR complex, thereby leading to resistance. In this scenario, AKT inhibitors serve as a treatment to reduce and dissociate activated AKT from EGFR. These findings demonstrate that lung cancer cells with wt-EGFR but BRG1 mutations are likely to respond better to AKT inhibitors than EGFR-TKIs. Therefore, pre-screening NSCLC cells with wt-EGFR for BRG1 status can inform appropriate treatment decisions, offering patients the choice to receive either EGFR-TKI or AKT inhibitor therapy. Our data underscores the significance of BRG1 in modulating EGFR and pAKT^Ser473^ expression and interaction, thus indicating that BRG1 mutation status dictates the treatment regimen and predicts therapy outcomes. Moreover, the increased expression of activated AKT in cells with mt-BRG1 and wt-EGFR suggests that targeting AKT might be more beneficial than targeting EGFR for therapy, further supporting our hypothesis aimed at unraveling the mechanism of resistance.

While our studies provide evidence for EGFR interaction with AKT contributing to EGFR-TKI resistance, and reintroduction of wild-type BRG1 expression restored sensitivity in BRG1-mutant A549 cells, the possibility of additional mechanisms modulated by BRG1 exists. For example, osimertinib treatment upregulated both HER3 and MET, which are known kinases that contribute to EGFR-TKI resistance, in mt-BRG1 A549 cells, but not in wt-BRG1 NCI-H358 cells. However, we did not investigate their role as it was beyond the scope of the study.

In summary, our study yields three interesting observations: firstly, BRG1 status dictates the response to EGFR-TKIs in NSCLC cells that are wild-type for EGFR; secondly, resistance to EGFR-TKIs is partially mediated by pAKT^(Ser473)^–EGFR complex formation; thirdly, NSCLC harboring mt-BRG1/wt-EGFR may benefit from AKT-targeted therapy instead of EGFR-TKI therapy. Thus, pre-screening wt-EGFR NSCLC patients for BRG1 status can inform the choice of targeted therapy and enable precision medicine approaches with improved treatment outcomes.

## 5. Conclusions

We have demonstrated a role for BRG1 in influencing the response to EGFR-TKIs in EGFR wild-type NSCLC cells. Additionally, our study also demonstrated physical interaction between EGFR and AKT in NSCLC cells that are mutant for BRG1, and in part contributed to resistance to EGFR-TKI. These results indicate that pre-screening of EGFR-wt NSCLC tumors for BRG1 status and pre-existence of the EGFR–AKT complex will aid in designing appropriate and improved treatment options for patients diagnosed with lung cancer. However, additional studies and clinical validation are required prior to applying an advanced precision medicine approach in this subset of the patient population.

## Figures and Tables

**Table 1:** List of oligonucleotide primers used in the study.

Primer Name	Primer Sequence	Application
Human BRG1	Forward 5′ AGC GAT GAC GTC TCT GAG GT Reverse 5′ GTA CAG GGA CAC CAG CCA CT	qRT-PCR
Human EGFR	Forward 5′ TAACAAGCTCACGCAGTTGG Reverse 5′ GTTGAGGGCAATGAGGACAT	qRT-PCR
18S	Forward5′ CAGCCACCCGAGATTGAGCA Reverse 5′ TAGTAGGGACGGGCGGTGTG	qRT-PCR

## Data Availability

Data is contained within the article or Supplementary Materials.

## Supplementary Materials

The following supporting information can be downloaded at https://www.mdpi.com/article/10.3390/cancers18010062/s1, Table S1. List of antibodies used in the study; Figure S1: BRG1 and EGFR are inversely correlated; Figure S2: Effect of BRG1 mutation on wt-EGFR expression in response to EGFR inhibitors; Figure S3: Impact of the overexpression and knock-out (KO) of BRG1 on wt-EGFR expression and the efficacy of gefitinib in A549 and NCI-H358 cells; Figure S4: Cytotoxic effect of gefitinib and AKT inhibitor (MK-2206) on mt-BRG1 A549 and H358-BRG1-KO cells; Figure S5: Cytotoxic effect of osimertinib and MK-2206 treatment and the impact of combination treatment on EGFR–pAKT interaction in A549 cells; Figure S6: Original and uncropped Western blot images of the figures included in the article.

## Abbreviations

The following abbreviations are used in this manuscript:

BRG1	Brahma-related gene 1
EGFR	Epidermal growth factor receptor
KO	Knock out
mt	Mutant
RTK	Receptor tyrosine kinase
SWI/SNF	SWItch/Surose non-fermentable
TKI	Tyrosine kinase inhibitor
TMA	Tissue microarray
wt	Wild-type
