# Supplementary material for: BRG1 (SMARCA4) Status Dictates the Response to EGFR Inhibitors in Wild-Type EGFR Non-Small Cell Lung Cancer
Source: Cancers (Basel). 2025 Dec 24;18(1):62. doi: 10.3390/cancers18010062 (PMC12784900; doi:10.3390/cancers18010062)

Supplementary Material

BRG1 (SMARCA4) Status Dictates the Response to EGFR Inhibitors in Wild-Type EGFR Non-Small Cell Lung Cancer

Rebaz Ahmed [1,2,†], Ranganayaki Muralidharan [1,‡], Narsireddy Amreddy [1,§], Akhil Srivastava [1,‖], Meghna Mehta [3], Janani Panneerselvam [1,¶], Rodrigo Orlandini de Castro [4], William L. Berry [5,6], Susmita Ghosh [1,6], Murali Ragothaman [1,6], Pawan Acharya [7,], Yan D. Zhao [6,7], Roberto Jose Pezza [4], Anupama Munshi [3,6,*] and Rajagopal Ramesh [1,2,6,*]**

1 Department of Pathology, The University of Oklahoma Health Sciences, Oklahoma City, OK 73104, USA; rebazawat@gmail.com (R.A.); ranganayaki.muralidharan@lily.com (R.M.); namreddy@cytovance.com (N.A.); askg2@health.missouri.edu (A.S.); janani@kopra.bio (J.P.); susmita-ghosh@ou.edu (S.G.); murali-ragothaman@ou.edu (M.R.)
2 Graduate Program in Biomedical Sciences, The University of Oklahoma Health Sciences, Oklahoma City, OK 73104, USA
3 Department of Radiation Oncology, The University of Oklahoma Health Sciences, Oklahoma City, OK 73104, USA; megsmehta@gmail.com
4 Cell Cycle and Cancer Biology Program, Oklahoma Medical Research Foundation, Oklahoma City, OK 73104, USA; orlandini.rodrigo@gmail.com (R.O.d.C.); roberto-pezza@omrf.org (R.J.P.)
5 Department of Surgery, The University of Oklahoma Health Sciences, Oklahoma City, OK 73104, USA; william-berry@ou.edu
6 OU Health Stephenson Cancer Center, The University of Oklahoma Health Sciences, Oklahoma City, OK 73104, USA; daniel-zhao@ou.edu
7 Biostatistics and Epidemiology, The University of Oklahoma Health Sciences, Oklahoma City, OK 73104, USA; pawanacharya@uabmc.edu
* Correspondence: anupama-munshi@ou.edu (A.M.); rajagopal-ramesh@ou.edu (R.R.); Tel.: +1-405-271-6102 (A.M.); +1-405-271-6101 (R.R.)
† Current address: Kurdistan Institution for Strategic Studies and Scientific Research, 60 Gullabax, 335 Shorsh Street, Sulaymaniyah 46001, Kurdistan Region, Iraq.
‡ Current address: Lilly Corporate Center, 839 S. Delaware Street, Indianapolis, IN 46285, USA.
§ Current address: Cytovance, Oklahoma City, OK 73104, USA.
‖ Current address: Department of Pathology and Anatomical Sciences, University of Missouri School of Medicine, Columbia, MO 65212, USA.
¶ Current address: Kopra Bio, 135 Mississippi St, San Francisco, CA 94107, USA.
** Current address: Department of Surgery, University of Alabama at Birmingham, 510 20th St, Suite #710, Birmingham, AL 35233, USA.

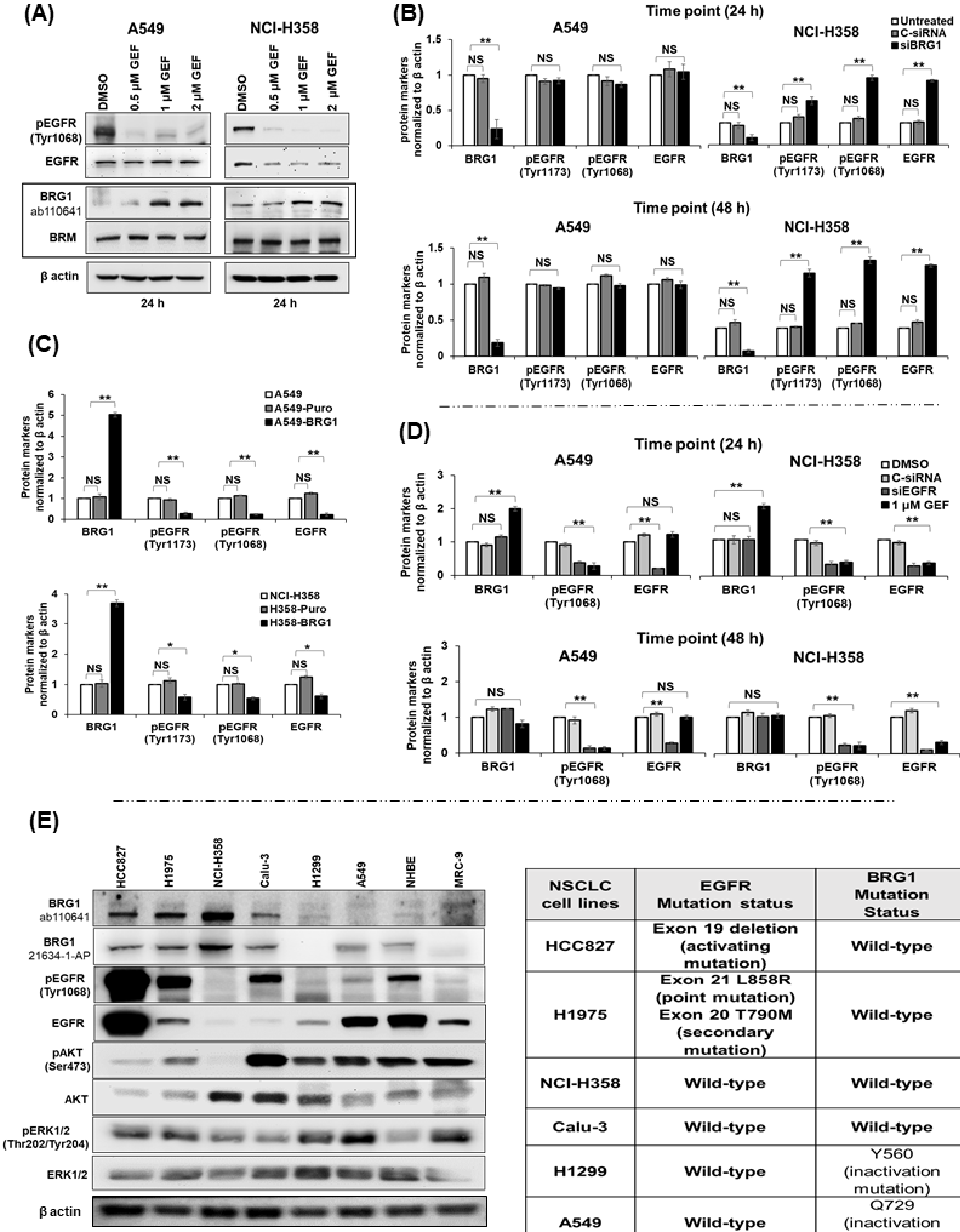

NSCLC cell lines	EGFR Mutation status	BRG1 Mutation Status
HCC827	Exon 19 deletion (activating mutation)	Wild-type
H1975	Exon 21 L858R (point mutation) Exon 20 T790M (secondary mutation)	Wild-type
NCI-H358	Wild-type	Wild-type
Calu-3	Wild-type	Wild-type
H1299	Wild-type	Y560 (inactivation mutation)
A549	Wild-type	Q729 (inactivation mutation)

Figure S1. BRG1 and EGFR are inversely correlated. (**A**), Effect of gefitinib on the expression of BRG1 and BRM. NCI-H358$^{\text{wt-EGFR/wt-BRG1}}$ and A549$^{\text{wt-EGFR/mt-BRG1}}$ cells were treated with gefitinib (0.5 µM, 1 µM, and 2 µM GEF) for 24 h, then analyzed for the expression of the selected markers by western blot analysis. DMSO treated cells were used as control. β actin was used as a loading control. (**B**), Western blot semi-quantification analysis of the selected markers in

siBRG1 treated, C-siRNA treated, and untreated A549 and NCI-H358 cells at 24 and 48 h is shown in a bar graph. (**C**), Western blot semi-quantification analysis of the selected markers in A549-BRG1 and H358-BRG1 cells. A549, A549-Puro, NCI-H358, and H358-Puro, cells were used as controls and are shown in a bar graph. (**D**), Western blot semi-quantification analysis of the selected markers in DMSO, C-siRNA, siEGFR, and gefitinib (1 µM GEF) treated A549 and NCI-H358 cells at 24 and 48 h, is shown in a bar graph. (**E**), Western blot analysis of the baseline expression level of selected markers in a panel of NSCLC cell lines. Comparison is to the Normal Human Bronchi Epithelial (NHBE) and normal human lung fibroblast (MRC-9) cells. β actin was used as loading a control. Table lists the mutational status of BRG1, EGFR and other oncogenic drivers in NSCLC cell lines. Error bar denotes SD; NS = not significant; *$p < 0.05$; **$p < 0.01$.

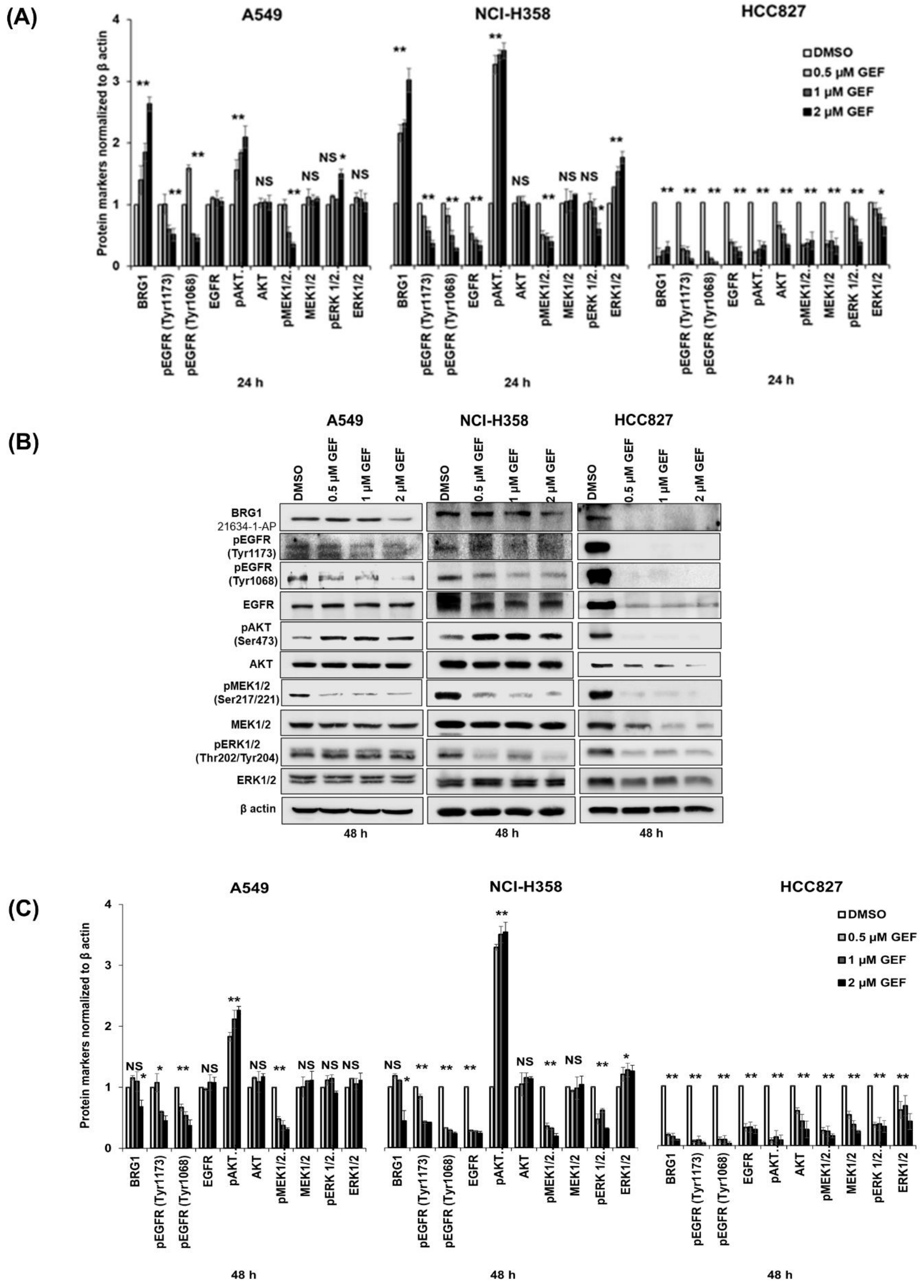
(A)
A549
NCI-H358
HCC827
Protein markers normalized to β actin
DMSO
0.5 μM GEF
1 μM GEF
2 μM GEF
BRG1
pEGFR (Tyr1173)
pEGFR (Tyr1068)
EGFR
pAKT.
AKT
pMEK1/2.
MEK1/2
pERK 1/2.
ERK1/2
24 h
(B)
A549
NCI-H358
HCC827
DMSO
0.5 μM GEF
1 μM GEF
2 μM GEF
BRG1
21634-1-AP
pEGFR (Tyr1173)
pEGFR (Tyr1068)
EGFR
pAKT (Ser473)
AKT
pMEK1/2 (Ser217/221)
MEK1/2
pERK1/2 (Thr202/Tyr204)
ERK1/2
β actin
48 h
(C)
A549
NCI-H358
HCC827
Protein markers normalized to β actin
DMSO
0.5 μM GEF
1 μM GEF
2 μM GEF
48 h

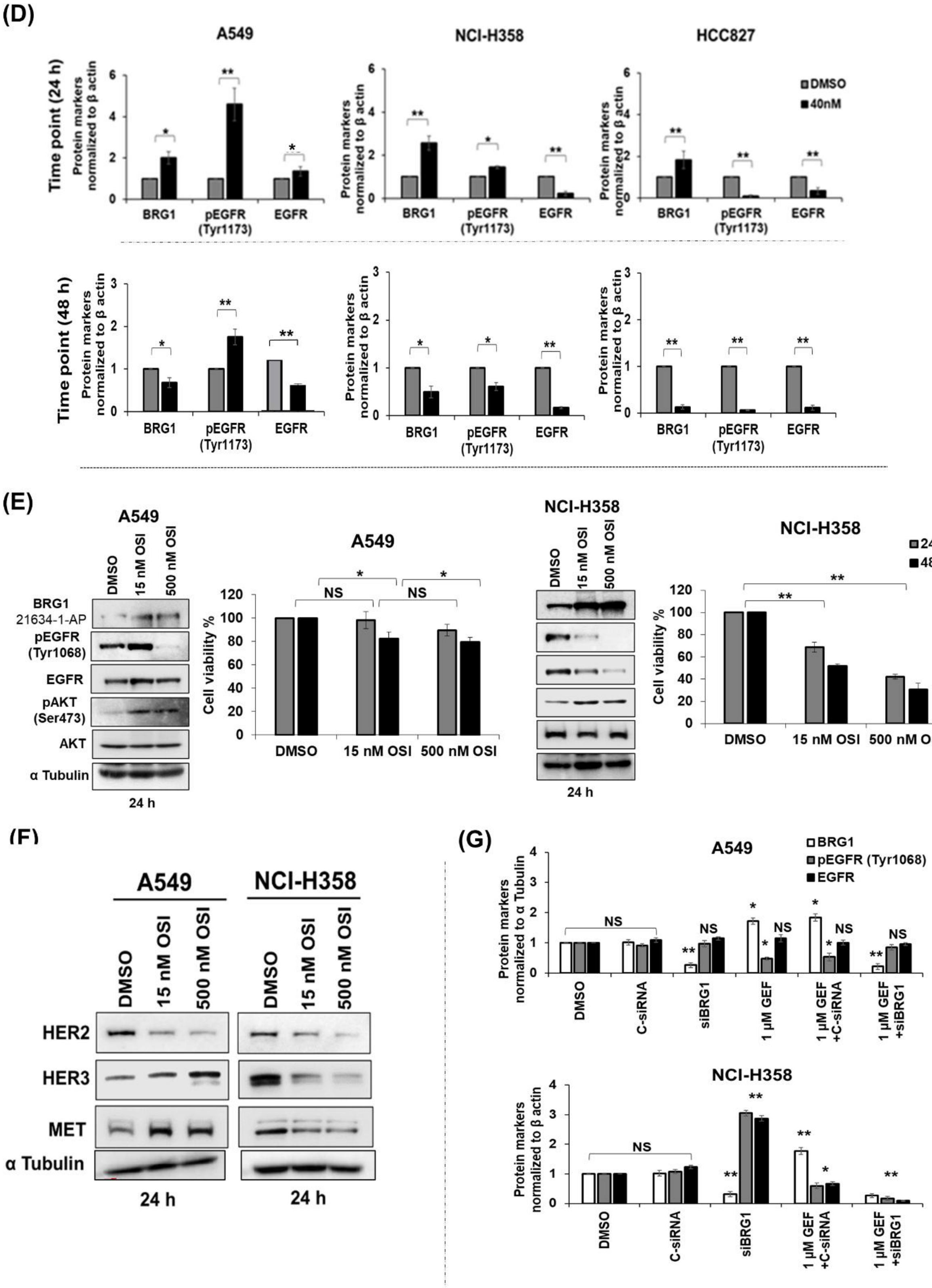
(D)
A549
NCI-H358
HCC827
Time point (24 h)
Time point (48 h)
Protein markers normalized to β actin
DMSO
40nM
BRG1
pEGFR (Tyr1173)
EGFR
(E)
A549
DMSO
15 nM OSI
500 nM OSI
BRG1
21634-1-AP
pEGFR (Tyr1068)
EGFR
pAKT (Ser473)
AKT
α Tubulin
24 h
Cell viability %
NS
NCI-H358
24 h
48 h
(F)
HER2
HER3
MET
(G)
BRG1
pEGFR (Tyr1068)
Protein markers normalized to α Tubulin
C-siRNA
siBRG1
1 µM GEF
1 µM GEF +C-siRNA
1 µM GEF +siBRG1

(H)

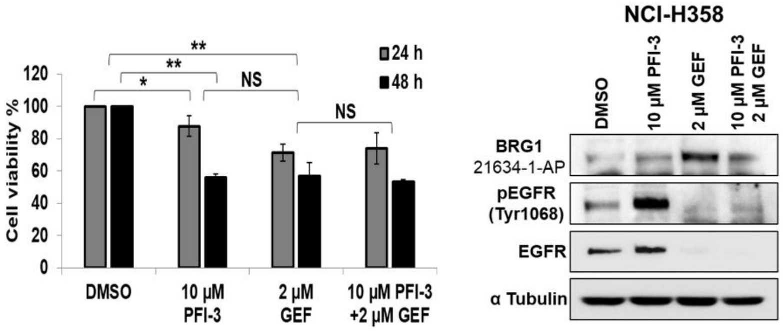

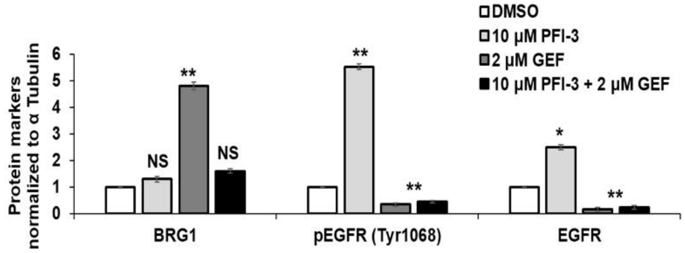

Figure S2. Effect of BRG1 mutation on wt-EGFR expression in response to EGFR inhibitors. (**A** and **C**), Western blot semi-quantification analysis of the selected markers in gefitinib (0.5 µM, 1 µM, and 2 µM GEF) treated A549, NCI-H358, and HCC827 cells at 24 and 48 h is shown in a bar graph. DMSO treated cells were used as control. (**B**), Western blot analysis of the selected markers in gefitinib (0.5 µM, 1 µM, and 2 µM) treated A549, NCI-H358, and HCC827 cells at 48 h. DMSO treated cells were used as control. β actin was used as a loading control. (**D**), Western blot semi-quantification analysis of the selected markers in gefitinib (40 nM GEF) treated A549, NCI-H358, and HCC827 cells at 24 h and 48 h is shown in a bar graph. DMSO treated cells were used as control. (**E**), Osimertinib (15 and 500 nM OSI) treated NCI-H358$^{wt\text{-}EGFR/wt\text{-}BRG1}$ and A549$^{wt\text{-}EGFR/mt\text{-}BRG1}$ cells were analyzed for cell viability at 24 and 48 h and shown in a bar graph. The expression of the selected markers in osimertinib -treated cells was analyzed at 24 h by western blot analysis. DMSO treated cells were used as control. α tubulin was used as a loading control. (**F**), Analysis of select protein kinases in osimertinib (15 and 500 nM) treated NCI-H358$^{wt\text{-}EGFR/wt\text{-}BRG1}$ and A549$^{wt\text{-}EGFR/mt\text{-}BRG1}$ cells at 24h. (**G**), Western blot semi-quantification analysis of the selected markers in siBRG1, gefitinib (1 µM), and siBRG1 (100 nM) plus gefitinib (1 µM GEF) treated cells at 24 h in A549 and NCI-H358 cells. DMSO, C-siRNA (100 nM), and C-siRNA plus gefitinib treated cells were used as control. (**H**)**,** Effect of bromodomain inhibition of BRG1 on EGFR expression and the efficacy of gefitinib. NCI-H358 cells were treated with a bromodomain inhibitor (10 µM PFI-3) for 6 h, then with gefitinib (2 µM GEF). After 24 and 48 h of combination treatment, cells were analyzed for cell viability and shown in a bar graph. In parallel, total cell lysates were analyzed for the expression of the selected markers by western blot analysis after 24 h post-combination treatment. DMSO treated cells were used as control. α tubulin was used as a loading control. Western blot semi-quantification analysis of the selected markers is shown in bar graphs. Error bar denotes SD; NS = not significant; *p< 0.05; **p< 0.01.

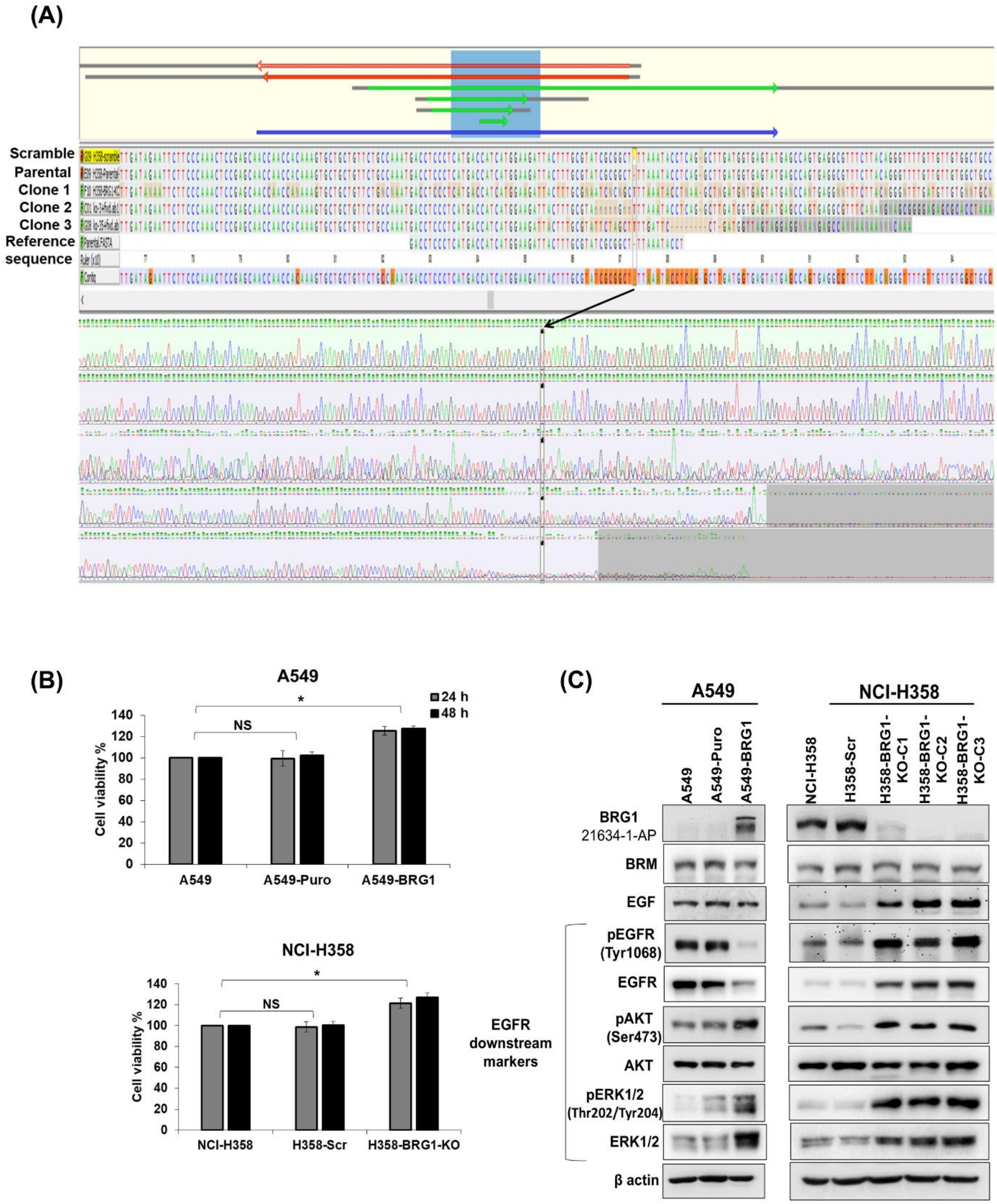
(A)
Scramble
Parental
Clone 1
Clone 2
Clone 3
Reference sequence
(B)
A549
24 h
48 h
Cell viability %
140
120
100
80
60
40
20
0
NS
*
A549
A549-Puro
A549-BRG1
NCI-H358
Cell viability %
140
120
100
80
60
40
20
0
NS
*
NCI-H358
H358-Scr
H358-BRG1-KO
(C)
A549
NCI-H358
A549
A549-Puro
A549-BRG1
NCI-H358
H358-Scr
H358-BRG1-KO-C1
H358-BRG1-KO-C2
H358-BRG1-KO-C3
BRG1
21634-1-AP
BRM
EGF
pEGFR (Tyr1068)
EGFR
pAKT (Ser473)
AKT
pERK1/2 (Thr202/Tyr204)
ERK1/2
β actin
EGFR downstream markers

(D)

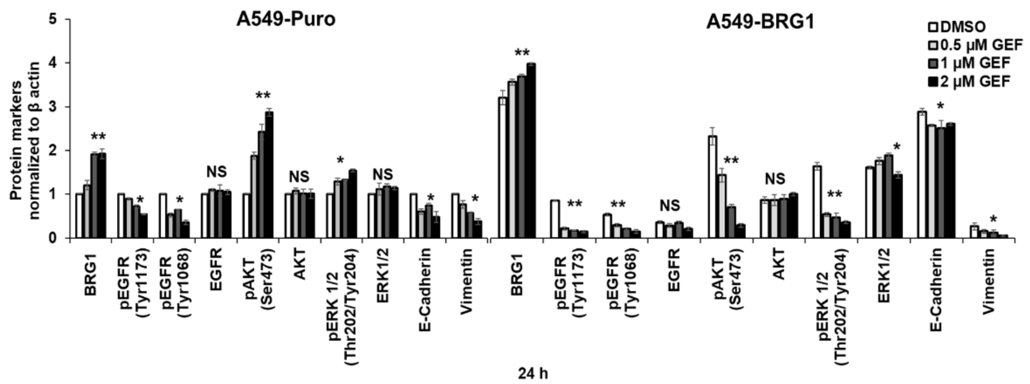

(E)

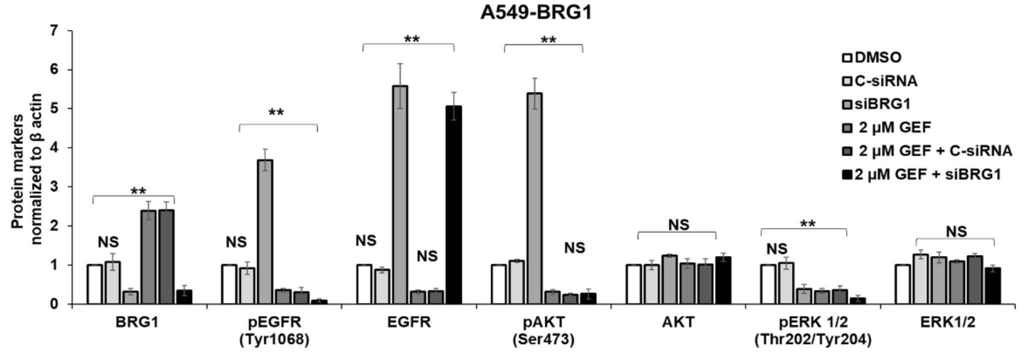

(F)

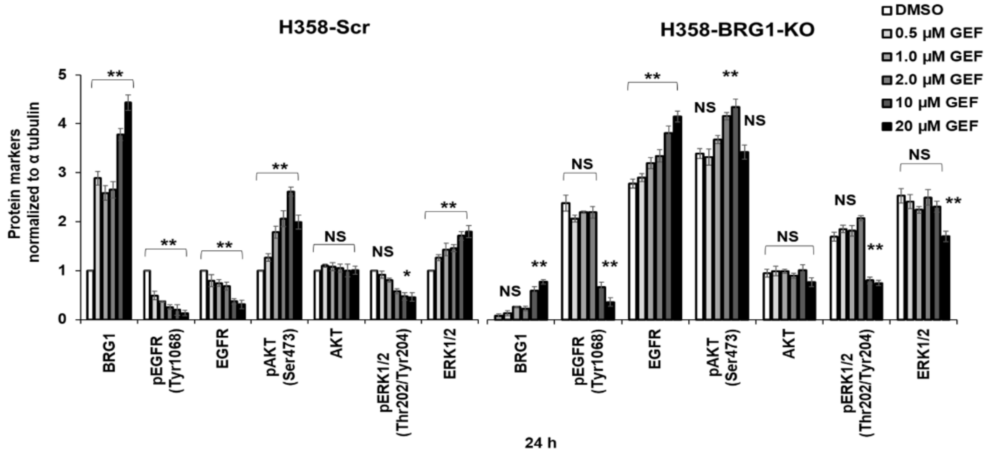

Figure S3. Impact of the overexpression and knock-out (KO) of BRG1 on wt-EGFR expression and the efficacy of gefitinib in A549 and NCI-H358 cells. (**A**), Stable BRG1 knock-out (KO) NCI-H358 cells were generated by the CRISPR-Cas9 technique. The generated CRISPR-Cas9-BRG1-KO NCI-H358 cells (H358-BRG1-KO; clones 1, 2, and 3) were verified for the alteration in the target sequence (GAAGATTACTTTGCGTATCG) of the BRG1 gene by Sanger sequencing. NCI-H358 (parental), H358-Scramble (Scr), and the BRG1 reference gene were used as controls to compare ambiguity in the target sequence. (**B**) The impact of BRG1 overexpression and BRG1-KO on the baseline expression of BRG1, BRM, EGFR, and EGFR downstream selected markers were tested by western blot analysis. A549, A549-Puro, NCI-H358, and H358-Scr cells were used as controls, respectively. β actin was used as a loading control. (**C**), Cell viability data for A549-BRG1 and H358-BRG1-KO cells were determined at 24 and 48 h and shown in a bar graph.

A549, A549-Puro, NCI-H358, and H358-Scr cells were used as controls, respectively. Error bar denotes SD; NS not significant; *$p < 0.05$ (**D**), Western blot semi-quantification analysis of the selected markers in gefitinib (0.5 µM, 1 µM, and 2 µM GEF) treated A549-Puro and A549-BRG1 cells at 24 h shown in a bar graph. DMSO treated cells were used as control. (**E**), Western blot semi-quantification analysis of the selected markers in siBRG1, gefitinib (2 µM GEF), and siBRG1 plus gefitinib combination treated A549-BRG1 cells at 24 h is shown in a bar graph. DMSO, C-siRNA, and C-siRNA plus gefitinib treated cells were used as control. (**F**), Western blot semi-quantification analysis of the selected markers in gefitinib (0.5 µM, 1 µM, 2 µM, 10 µM, and 20 µM GEF) treated H358-Scr and H358-BRG1-KO (clone #1) cells for 24 h is shown in a bar graph. DMSO treated cells were used as control. Error bar denotes SD; NS = not significant; *$p < 0.05$; **$p < 0.01$.

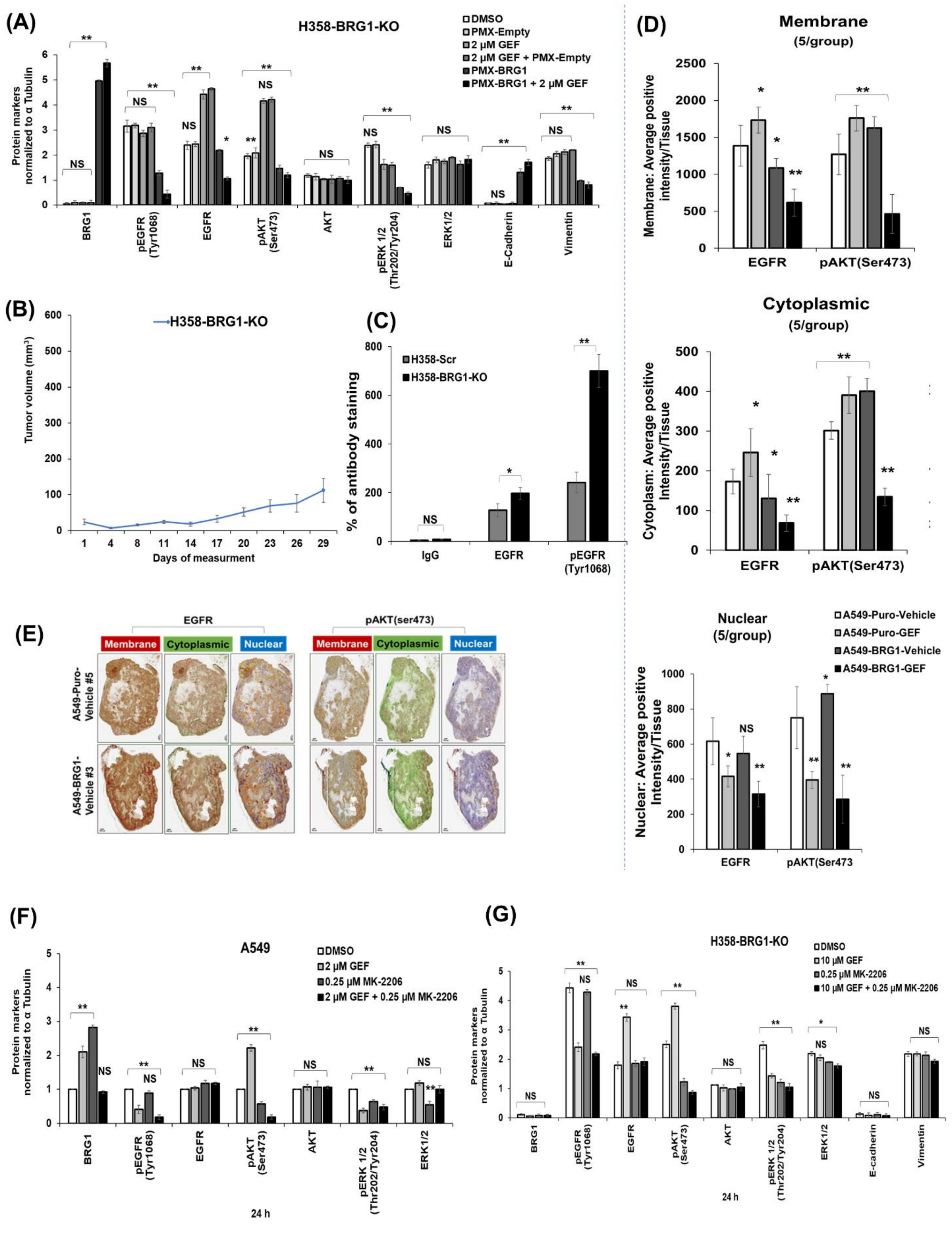

Figure S4. Cytotoxic effect of gefitinib and AKT inhibitor (MK-2206) on mt-BRG1 A549 and H358-BRG1-KO cells. (**A**), Western blot semi-quantification analysis of the selected markers in gefitinib (2 μM GEF), PMX-BRG1, and gefitinib plus PMX-BRG1 treated H358-BRG1-KO (clone #1) cells at 24 h is shown in a bar graph. DMSO, PMX-empty, and PMX-empty plus gefitinib combination treated cells were used as controls. (**B**), The tumor growth curve in nude mice bearing H358-BRG1-KO tumor xenografts. Representative gross mice bearing H358-Scr tumor images from

the vehicle or gefitinib treatment are shown. (**C**), Immunostaining detection of EGFR and $pEGFR^{Tyr1068}$ in H358-Scr and H358-BRG1-KO (clone #1) by flow cytometry is shown in a bar graph. IgG immunostaining was used as control. (**D**), The EGFR and $pAKT^{Ser473}$ interaction confirmation *in vivo.* The A549-Puro and A549-BRG1 tumor tissues treated with DMSO (vehicle) or gefitinib were stained and quantified using the Aperio Scan Scope Image Analysis System for EGFR and $pAKT^{Ser473}$ positivity in different cellular compartments (membrane, cytoplasmic and nuclear) and is shown in a bar graph. DMSO (vehicle) treated A549-Puro, and A549-BRG1 tumor tissues were used as control. (**E**), Representative images of a color-coded annotation in the membrane (red), cytoplasmic (green), and nuclear (blue) of EGFR and $pAKT^{Ser473}$ staining pattern in DMSO (vehicle) treated A549-Puro and A549-BRG1 tumor tissues are shown at 10 X magnification; Scale bar, 1000 µm. (**F** and **G**), Western blot semi-quantification analysis of the selected markers in gefitinib (2 µM or 10 µM GEF), MK-2206 (0.25 µM), and gefitinib plus MK-2206 treated A549 and H358-BRG1-KO cells, respectively at 24 h is shown in a bar graph. DMSO treated cells were used as control. Error bar denotes SD; NS = not significant; *$p < 0.05$; **$p < 0.01$.

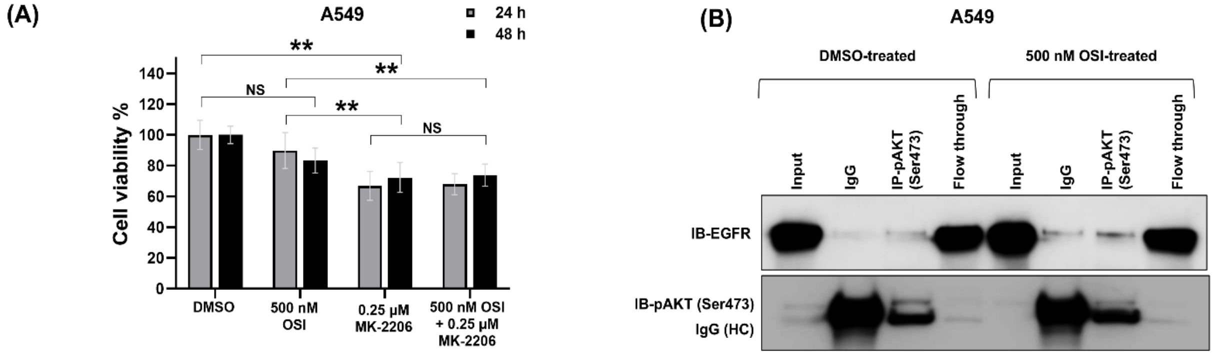

Figure S5: Effect of osimertinib on cytotoxicity and EGFR-AKT complex formation in A549 cells. **(A)** The cytotoxic effects of osimertinib (500 nM), MK-2206 (250 nM), and their combination were evaluated in parental A549 cells by cell viability assay at 24 h and 48 h. Cells treated with MK-2206 showed significant reduction in cell viability compared to osimertinib treated and DMSO treated cells (p<0.01). Combination therapy showed no significant enhancement in cytotoxicity when compared to MK-2206 alone. At 48 h, treatment with osimertinib and MK-2206 showed significant cytotoxicity compared to DMSO control (*p<0.05). However, combination treatment showed no significant cytotoxicity when compared to individual treatments. Error bar denotes SD; NS = not significant; *p<0.05; **p<0.01. (**B**) A549 cells treated with osimertinib (500 nM) for 24 h were analyzed for EGFR-pAKT^{Ser473} complex formation by immunoprecipitation and western blot analysis. Mouse IgG served as a negative control. Osimertinib treatment did not effectively disrupt the EGFR-pAKT^{Ser473} interaction.

Table S1. List of antibodies used in the study

Antibody Name	Vendor	Catalog number	Dilution v/v
BRG1 (Full sequence)	Proteintech Group; IL	21634-1-AP	1:2000 W. B[1]
BRG1 (ab110641)	Abcam; MA	ab110641	1:1000 W. B<br>1:50 IHC[2]
EGFR	Cell Signaling Technology; MA	4267	1:1000 W. B<br>1:250 IHC
pAKT (ser473)	Cell Signaling Technology; MA	4060	1:1000 W. B
AKT	Cell Signaling Technology; MA	9272	1:1000 W. B
pERK1/2(Thr202/Tyr204)	Cell Signaling Technology; MA	9101	1:1000 W. B
ERK1/2	Cell Signaling Technology; MA	9102	1:1000 W. B
BRM	Cell Signaling Technology; MA	11966	1:1000 W. B
ARID1-A	Cell Signaling Technology; MA	12354	1:1000 W. B
BAF155	Cell Signaling Technology; MA	9502	1:1000 W. B
SNF5	Cell Signaling Technology; MA	8745	1:1000 W. B
pEGFR (Tyr1068)	Cell Signaling Technology; MA	3777	1:1000 W. B<br>1:3500 IHC
pEGFR (Tyr1173)	Cell Signaling Technology; MA	4407	1:1000 W. B
Lamin-B1	Cell Signaling Technology; MA	13435	1:1000 W. B
MEK1/2	Cell Signaling Technology; MA	9122	1:1000 W. B
pMEK1/2(Ser217/221)	Cell Signaling Technology; MA	9121	1:1000 W. B
β actin	Sigma Aldrich; MO	A5316	1:2000 W. B
α Tubulin	Cell Signaling Technology; MA	3873	1:1000 W. B
EGF	Abcam; MA	ab206423	1:1000 W. B
E-Cadherin	Cell Signaling Technology; MA	14472	1:1000 W. B
Vimentin	Cell Signaling Technology; MA	5741	1:1000 W. B
pAKT (Ser473)	Abcam; MA	ab81283	1:50 IHC
AKT	Abcam; MA	ab32505	1:100 IHC
pERK1/2(Thr202/Tyr204)	Abcam; MA	ab223500	1:100 IHC
ERK1/2	Abcam; MA	ab17942	1:100 IHC
Ki67	Abcam; MA	ab833	1:100 IHC
EGFR	Cell Signaling Technology; MA	2256	1:1000 W. B<br>1:500 I. P[3]
pAKT (Ser473)	Cell Signaling Technology; MA	12694	1:1000 W.B.<br>1:500 I. P
Mouse IgG	Santa Cruz Biotechnology; TX	Sc-2025	1:500<br>I. P
EGFR (Anti-EGFR Alexa Fluor® 488)	Abcam; MA	Ab193244	1:500 I. F[4]<br>1:100 FACS
EGFR (anti-EGFR phospho (Tyr1068)) (Anti-Alexa Fluor® 488)	Abcam; MA	Ab205827	1:100 FACS[5]
BRG1 (Anti-EGFR Alexa Fluor® 647)	Abcam; MA	ab196535	1:1000 I. F
Rabbit IgG (Alexa Fluor® 488)	Abcam; MA	Ab199091	1:100 I. F

[1]W.B-Western blotting, [2]IHC-Immunohistochemistry, [3]I.P-Immunoprecipitation, [4]I.F-Immunofluorescence, and [5]FACS-Fluorescence activated cell sorting

Figure S6. Original and uncropped Western blot images for the figures shown in the main manuscript and supplementary section.

Supplementary Figure S6 – Original Western blot images shown in Figure 1B

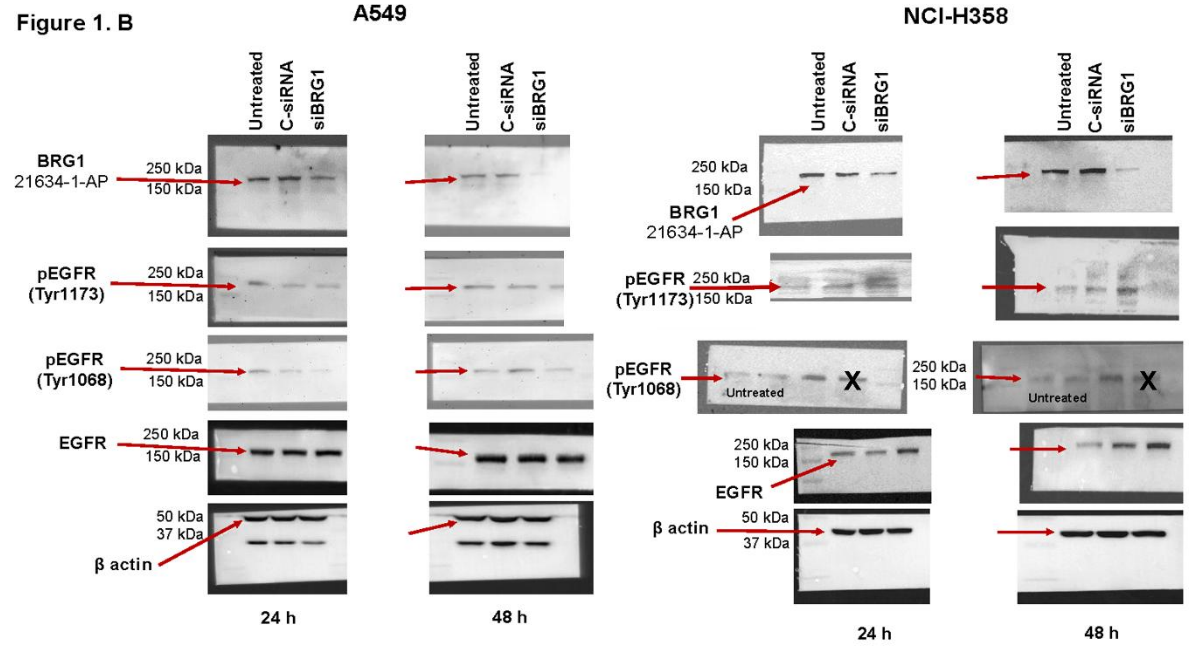

Supplementary Figure S6 – Original Western blot images shown in Figure 1E

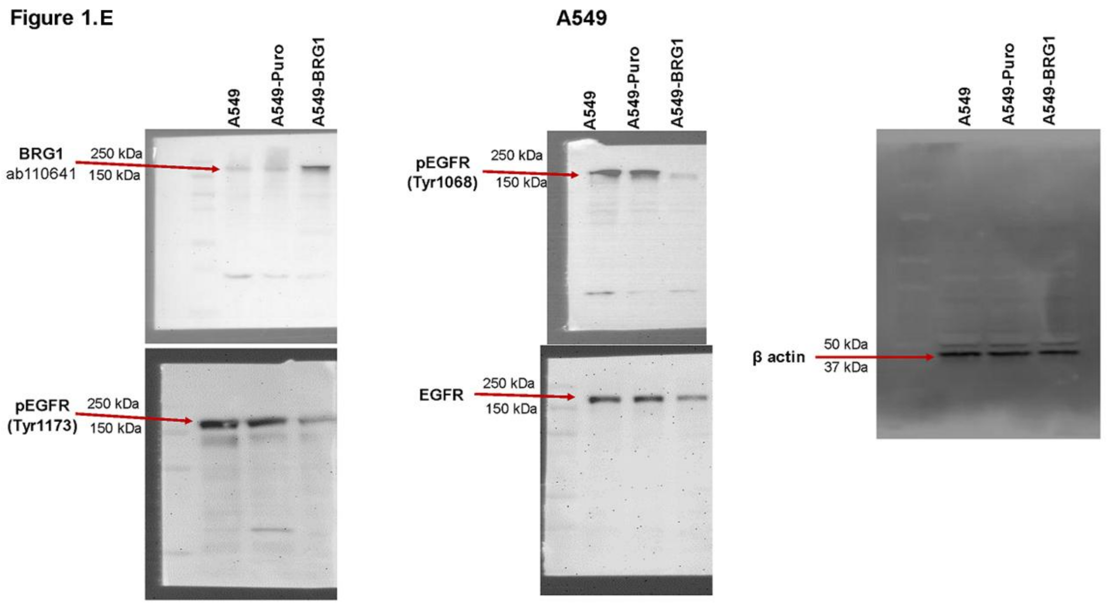

Supplementary Figure S6 – Original Western blot images shown in Figure 1E (continued)

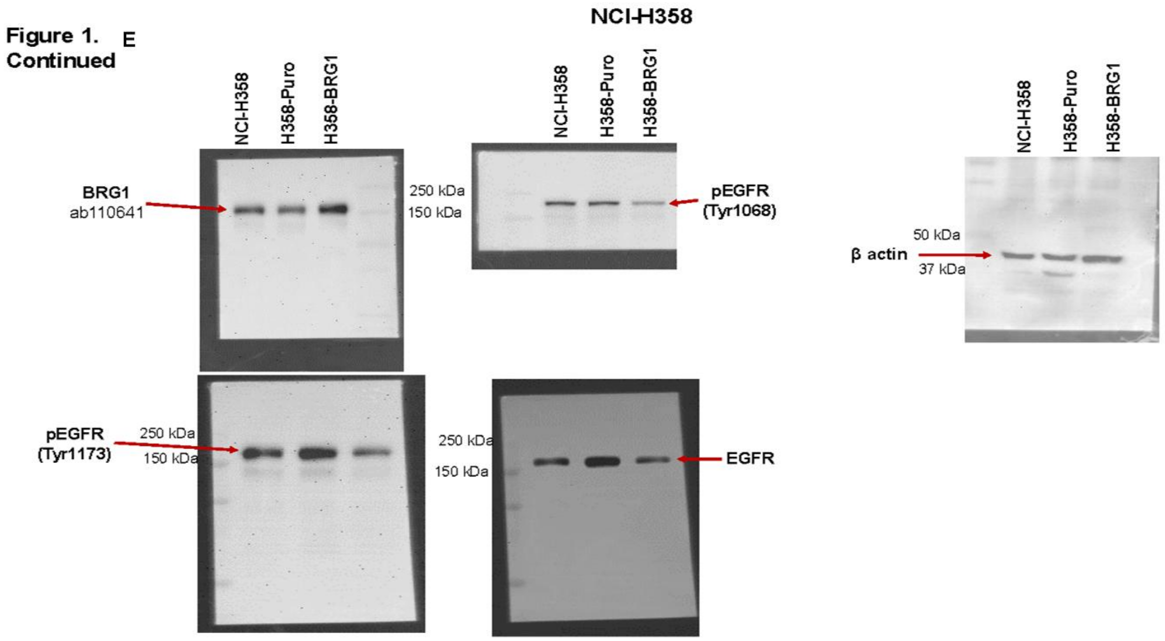

Supplementary Figure S6 – Original Western blot images shown in Figure 1F

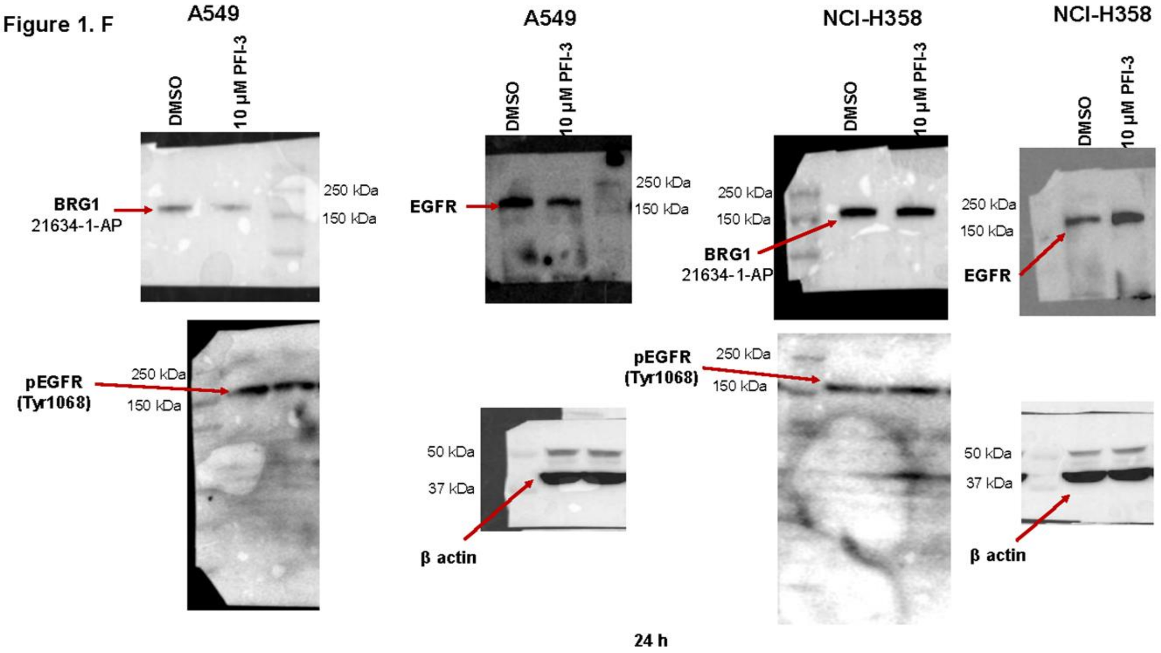

Supplementary Figure S6 – Original Western blot images shown in Figure 2B

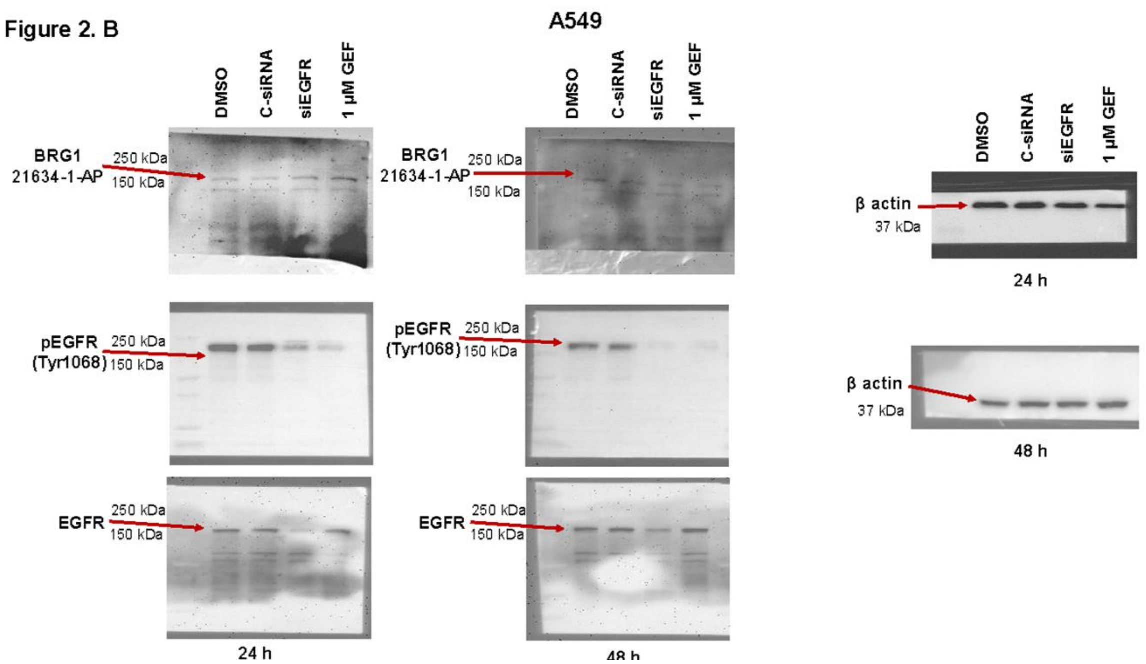

Supplementary Figure S6 – Original Western blot images shown in Figure 2B (continued)

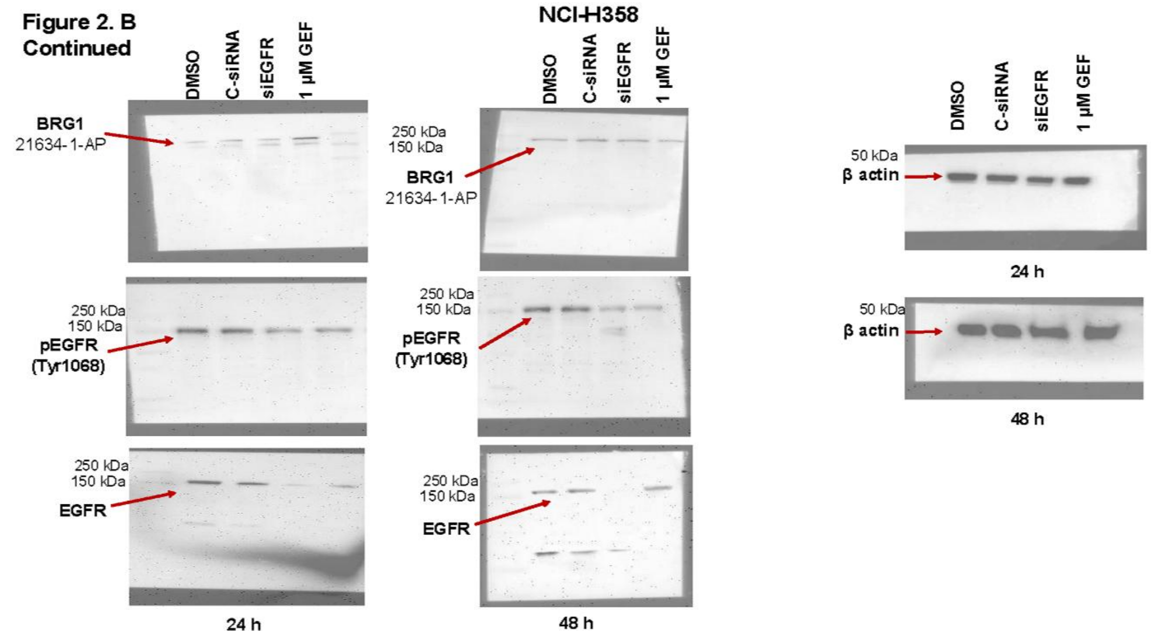

Supplementary Figure S6 – Original Western blot images shown in Figure 2C

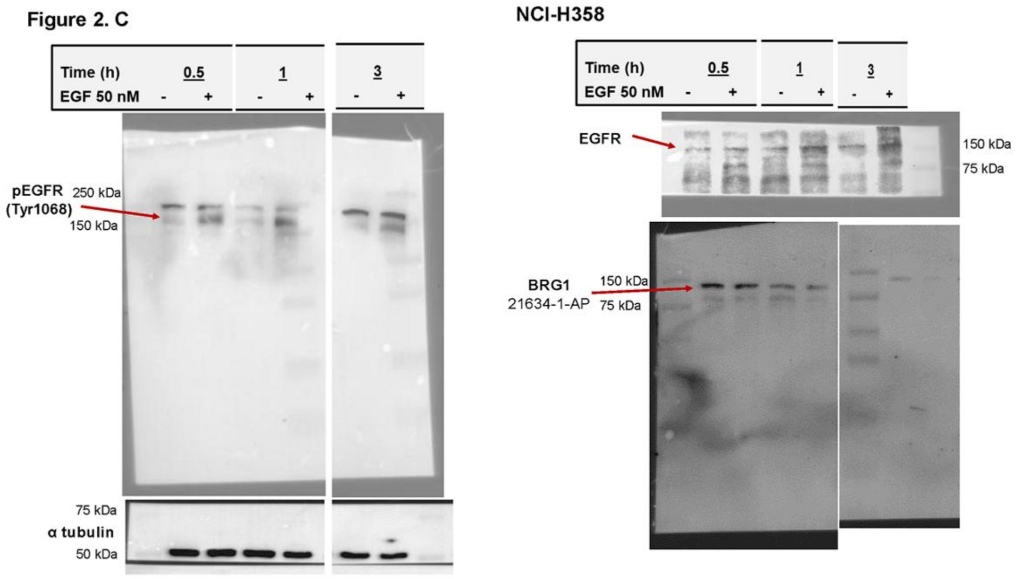

Supplementary Figure S6 – Original Western blot images shown in Figure 3B

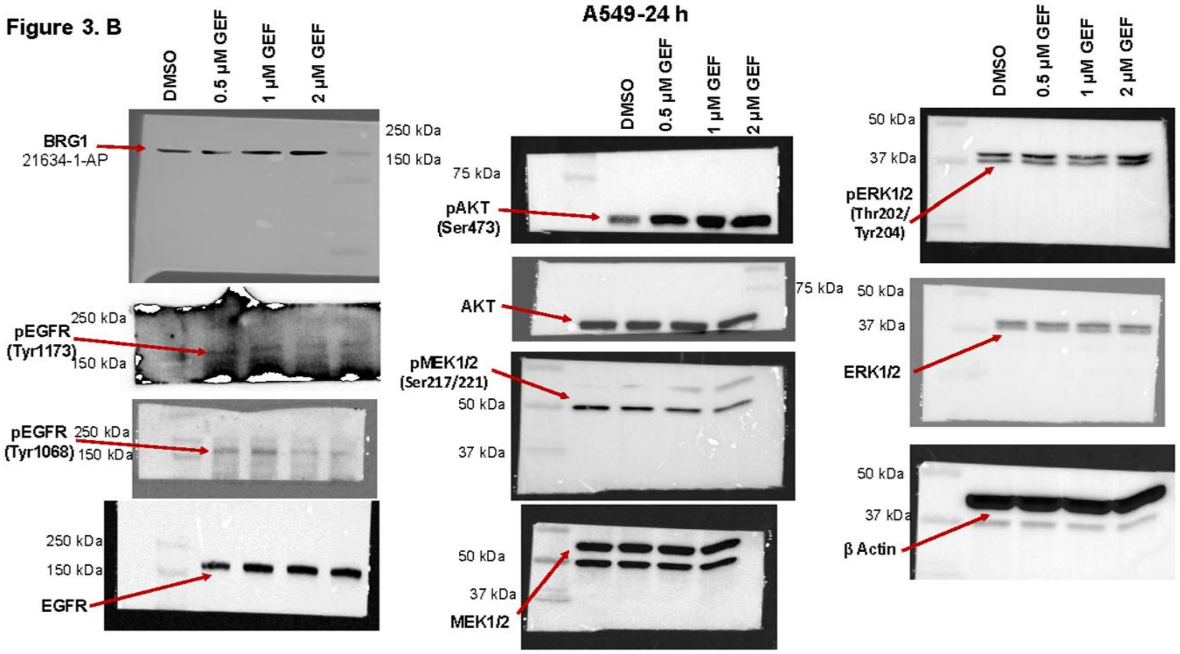

Supplementary Figure S6 – Original Western blot images shown in Figure 3B (continued)

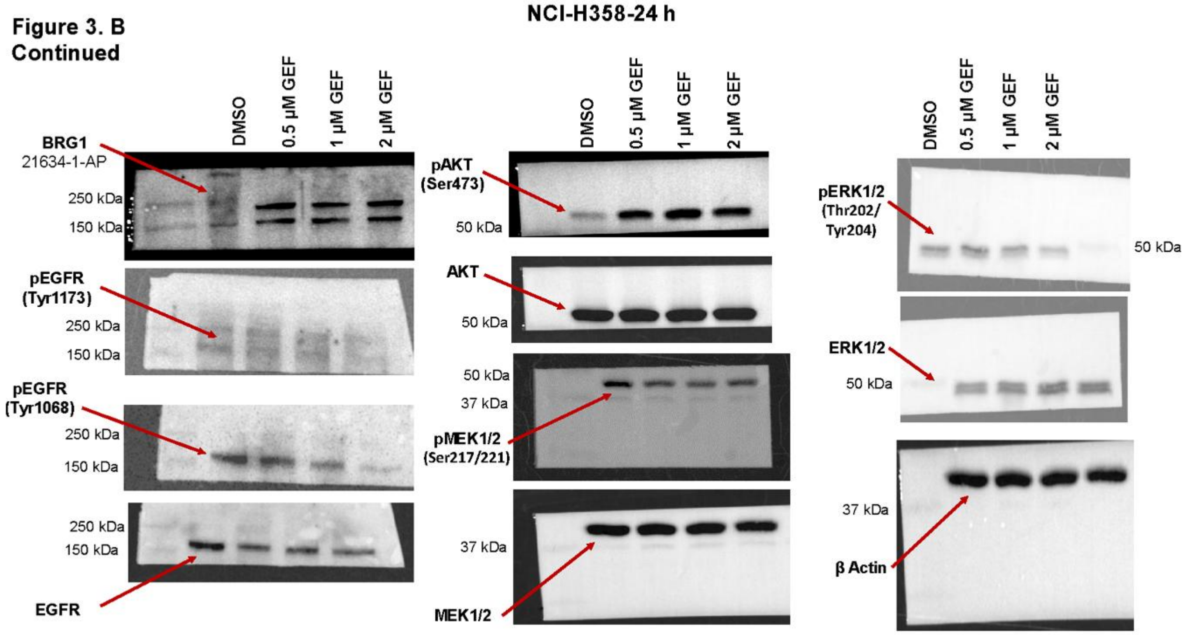

Supplementary Figure S6 – Original Western blot images shown in Figure 3B (continued)

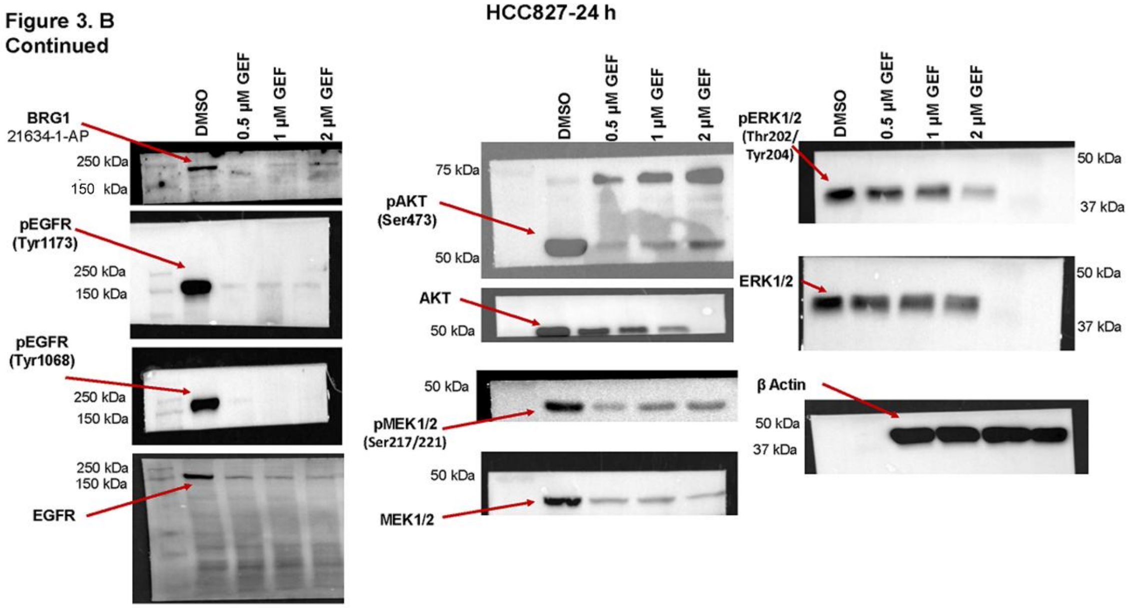

Supplementary Figure S6 – Original Western blot images shown in Figure 3C

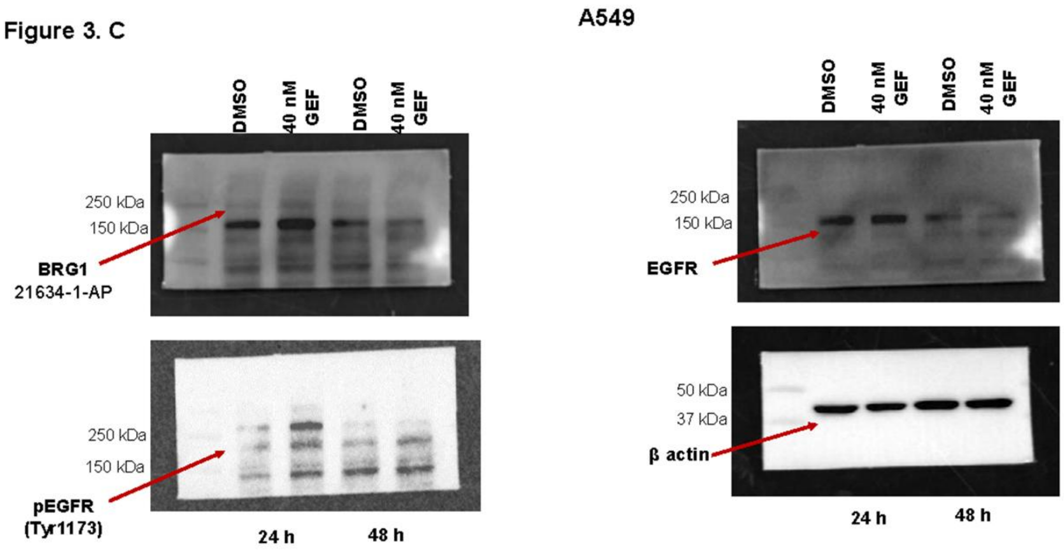

Supplementary Figure S6 – Original Western blot images shown in Figure 3C (continued)

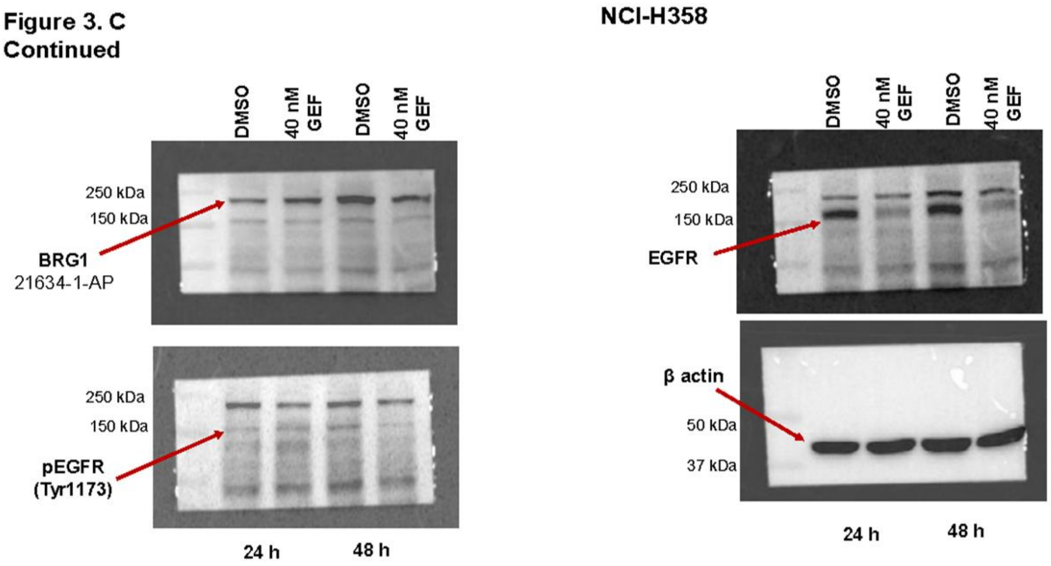

Supplementary Figure S6 – Original Western blot images shown in Figure 3C (continued)

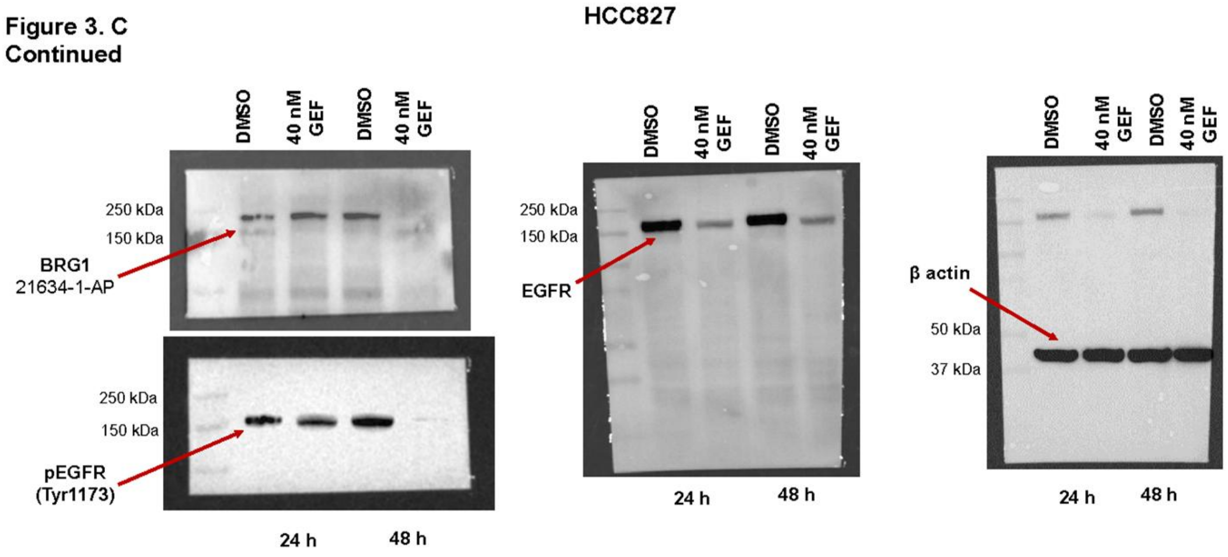

Supplementary Figure S6 – Original Western blot images shown in Figure 3D

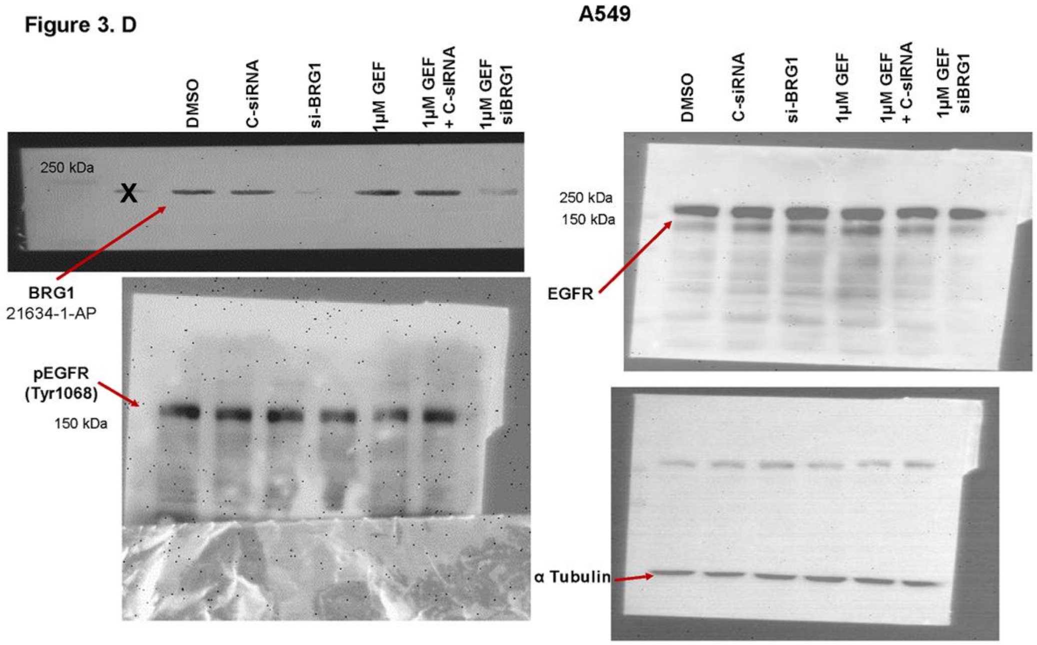

Supplementary Figure S6 – Original Western blot images shown in Figure 3D (continued)

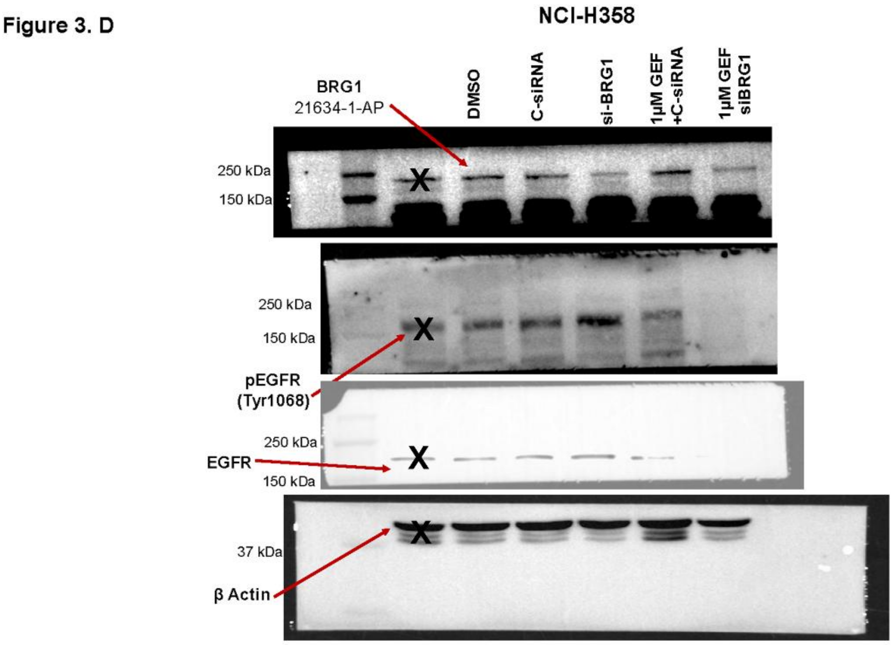

Supplementary Figure S6 – Original Western blot images shown in Figure 4A

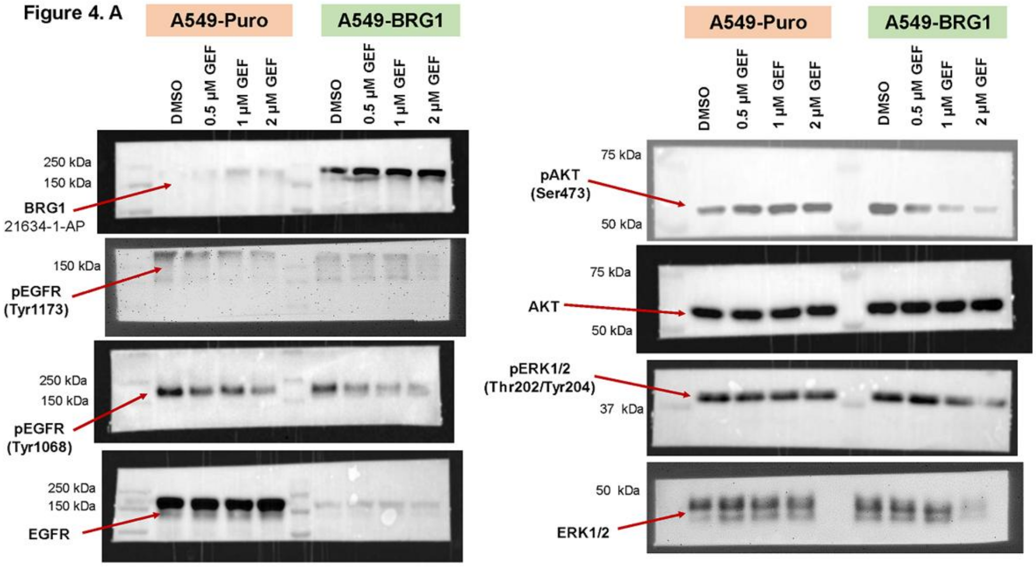

Supplementary Figure S6 – Original Western blot images shown in Figure 4A (continued)

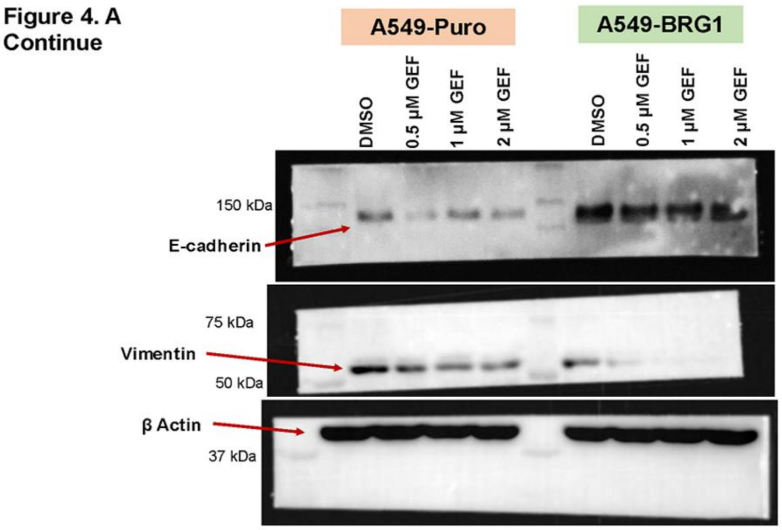

Supplementary Figure S6 – Original Western blot images shown in Figure 4B

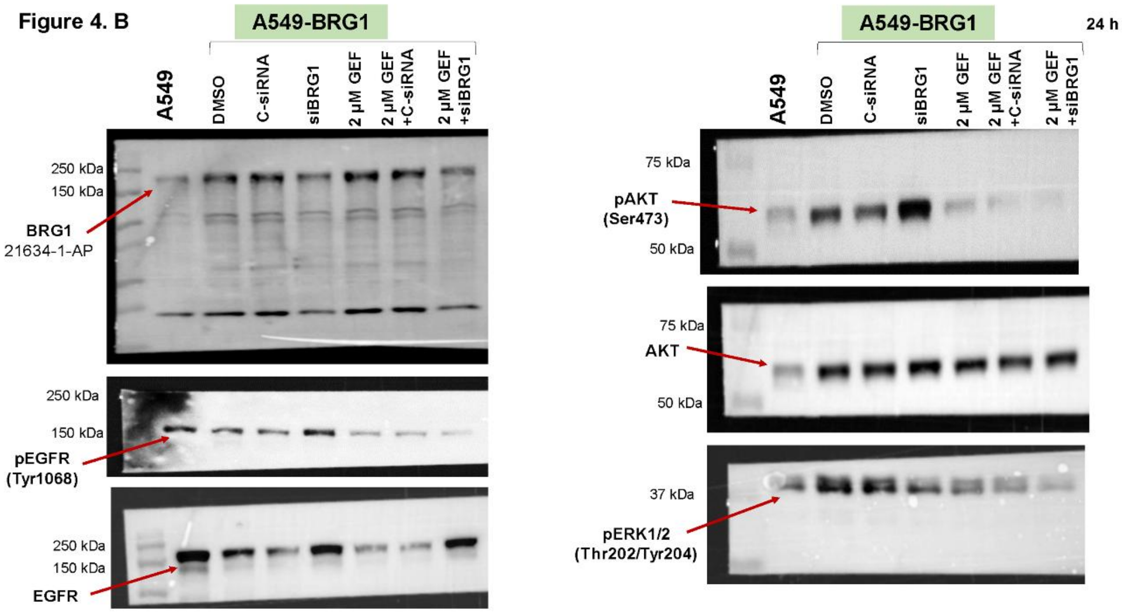

Supplementary Figure S6 – Original Western blot images shown in Figure 4B (continued)

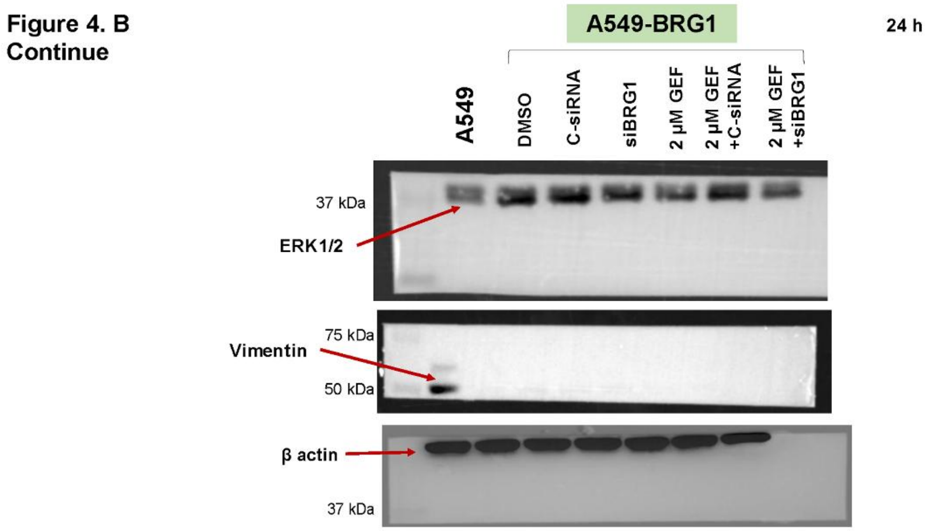

Supplementary Figure S6 – Original Western blot images shown in Figure 4E

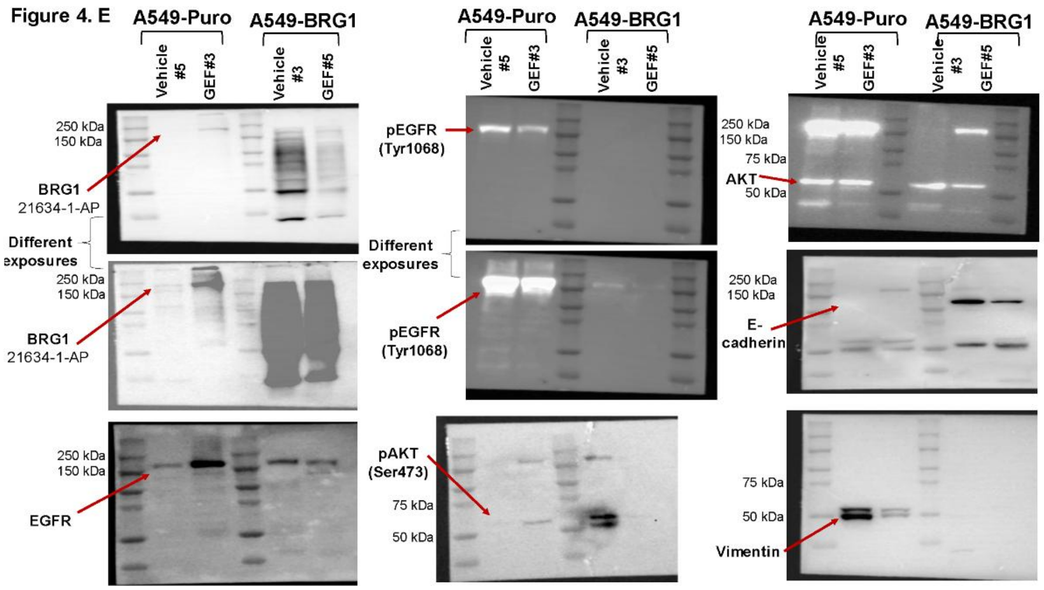

Supplementary Figure S6 – Original Western blot images shown in Figure 4E (continued)

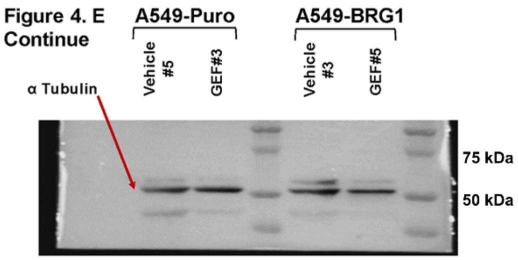

Supplementary Figure S6 – Original Western blot images shown in Figure 5A

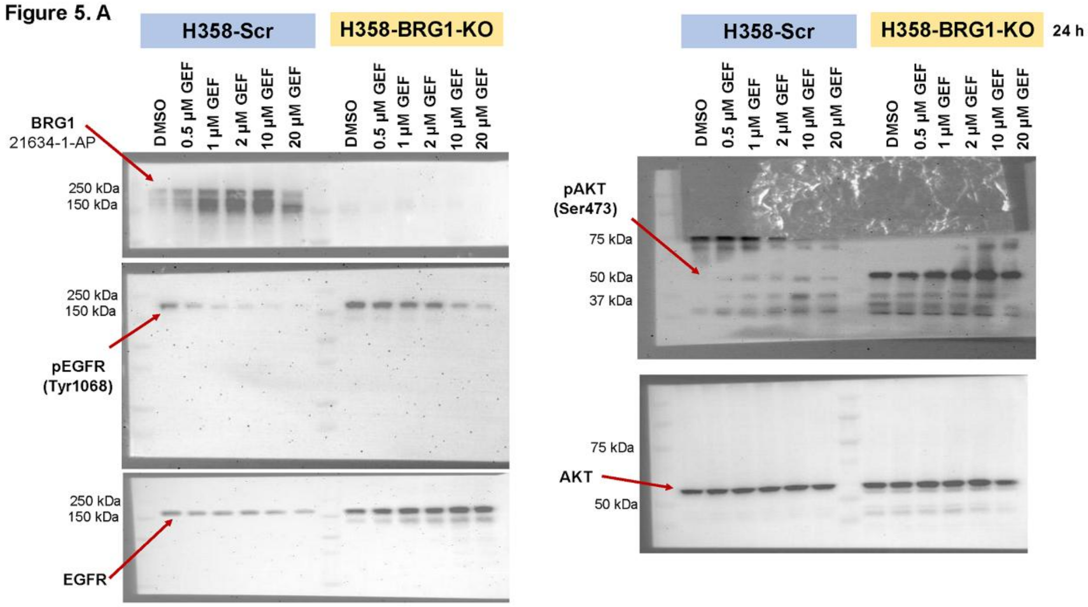

Supplementary Figure S6 – Original Western blot images shown in Figure 5A (continued)

Figure 5. A Continued

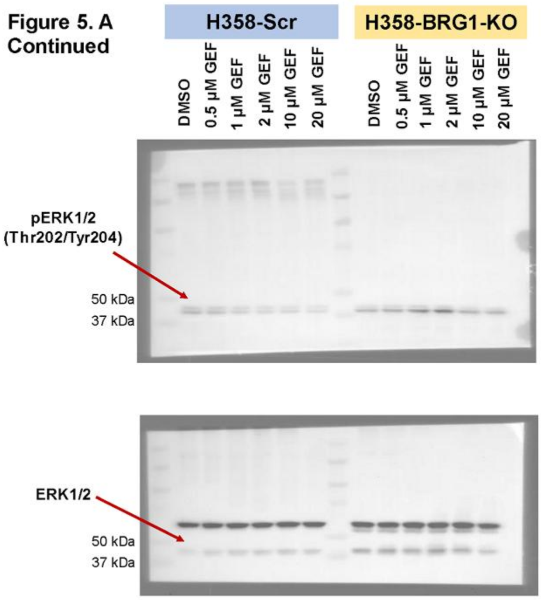

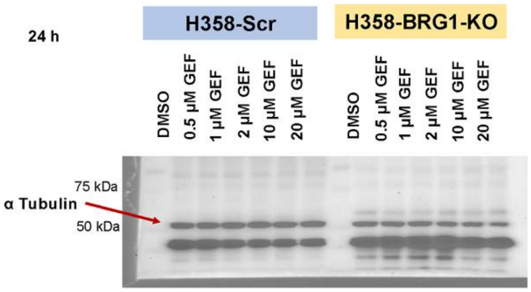

Supplementary Figure S6 – Original Western blot images shown in Figure 5C

Figure 5. C

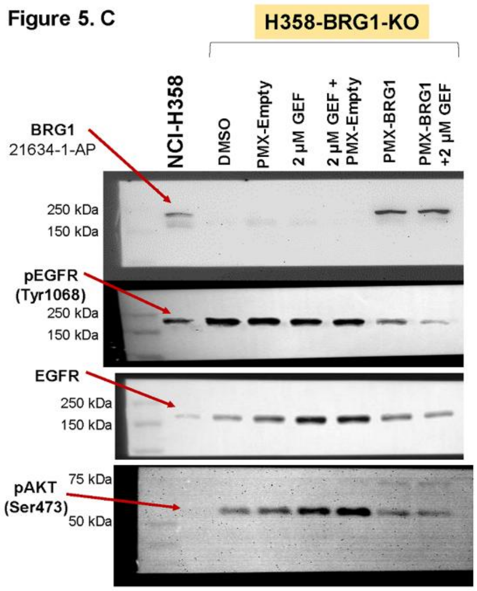

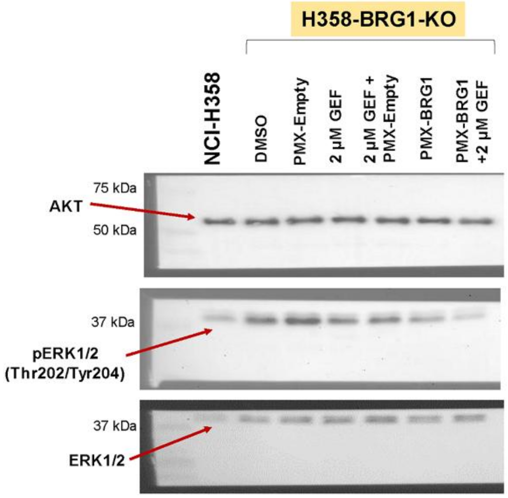

Supplementary Figure S6 – Original Western blot images shown in Figure 5C (continued)

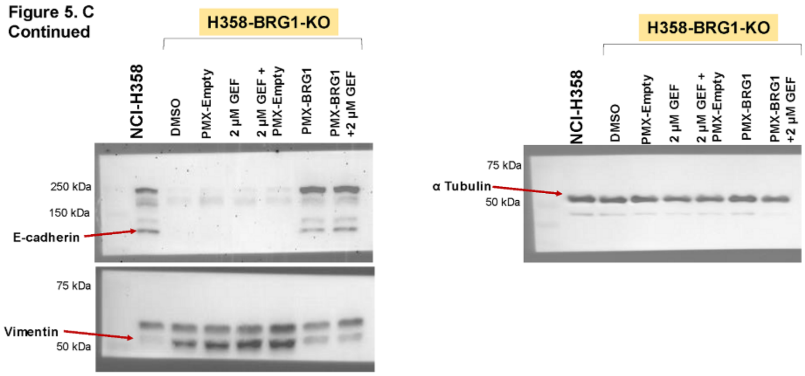

Supplementary Figure S6 – Original Western blot images shown in Figure 6A

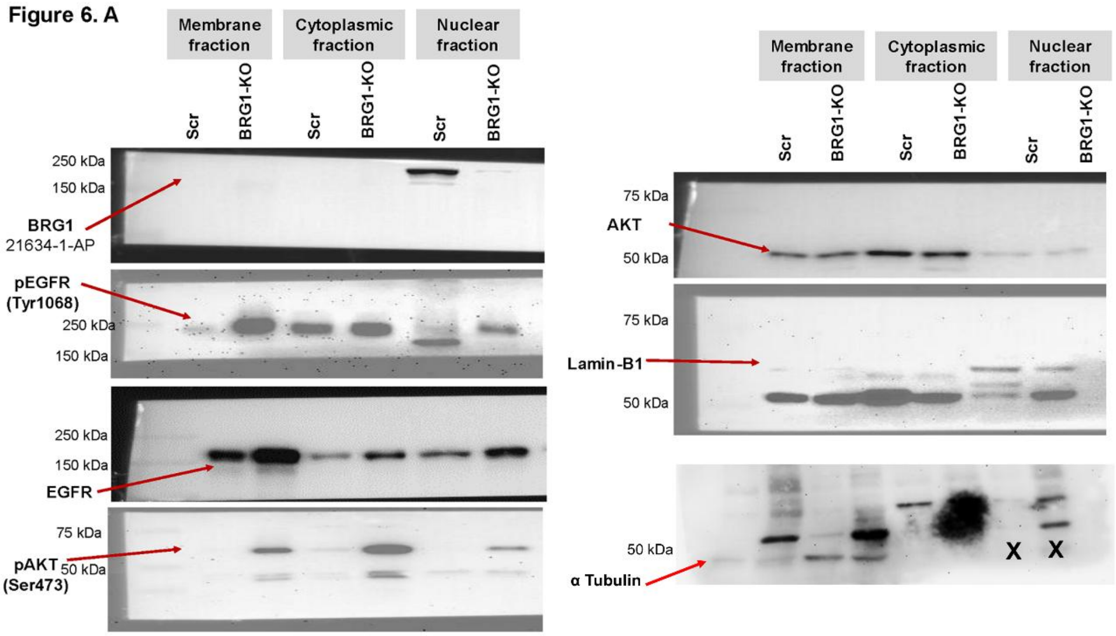

Supplementary Figure S6 – Original Western blot images shown in Figure 6B

Figure 6. B

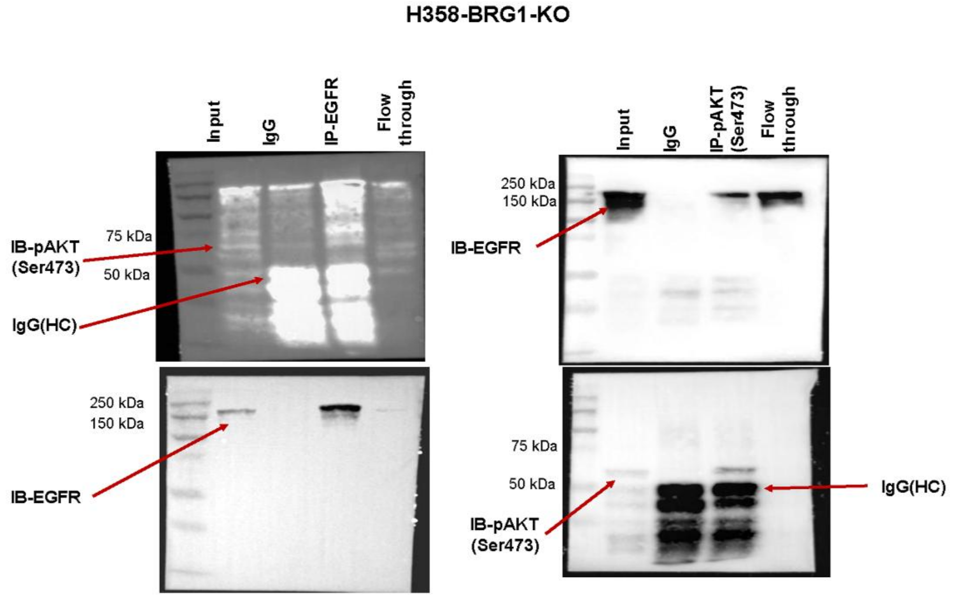

Supplementary Figure S6 – Original Western blot images shown in Figure 6C and E

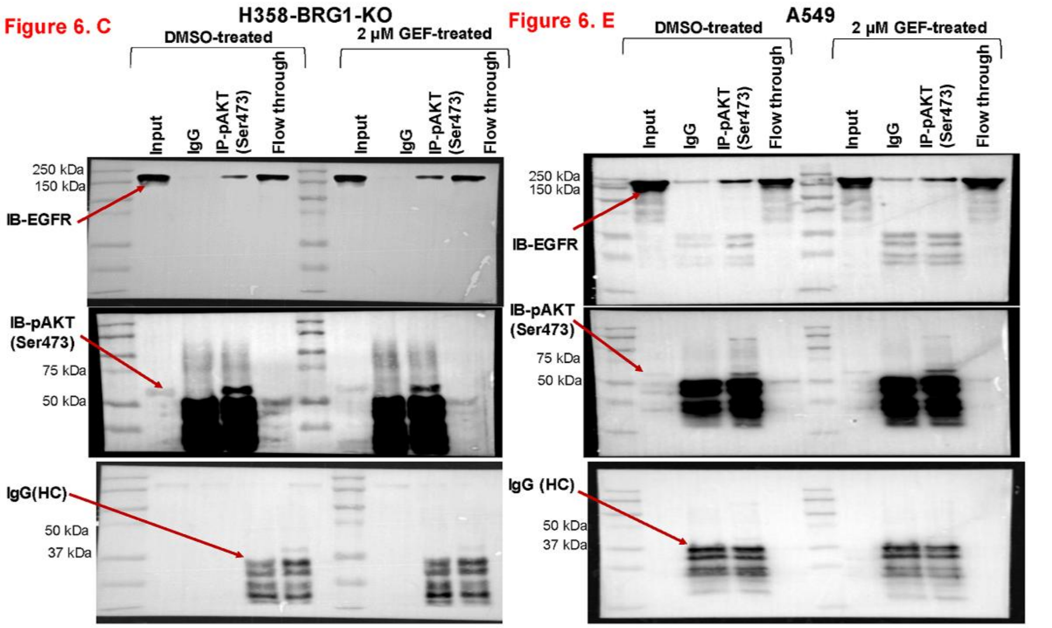

Supplementary Figure S6 – Original Western blot images shown in Figure 6D and F

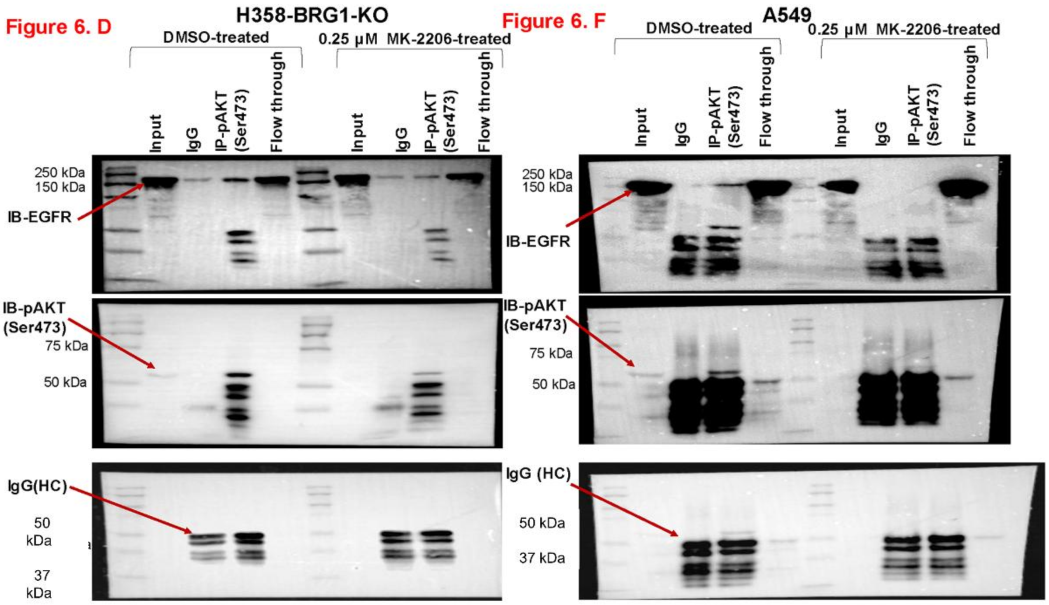

Supplementary Figure S6 – Original Western blot images shown in Figure 6G

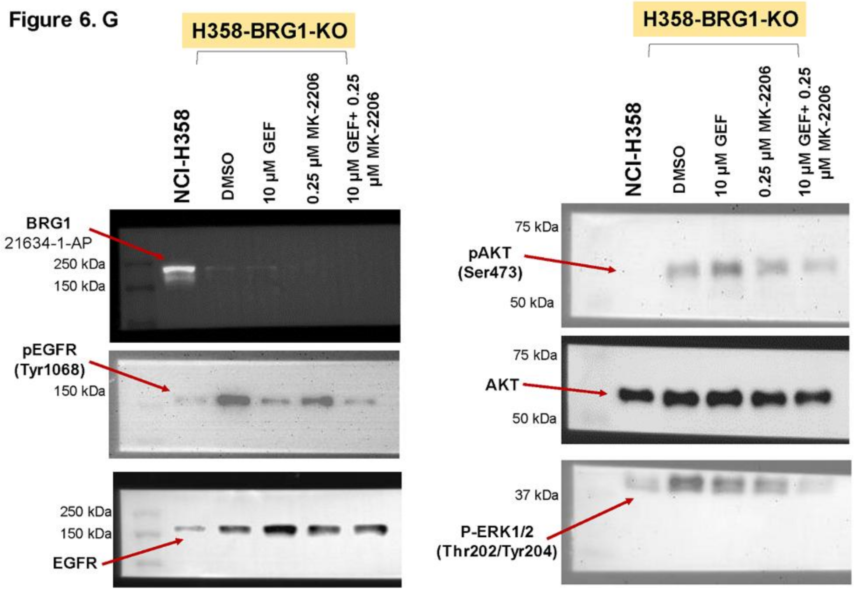

Supplementary Figure S6 – Original Western blot images shown in Figure 6G (continued)

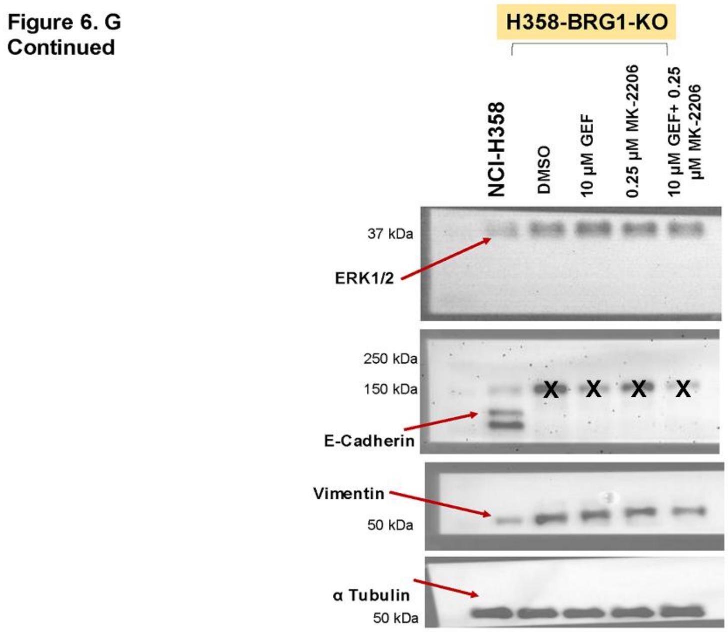

Supplementary Figure S6 – Original Western blot images shown in Figure 6G (continued)

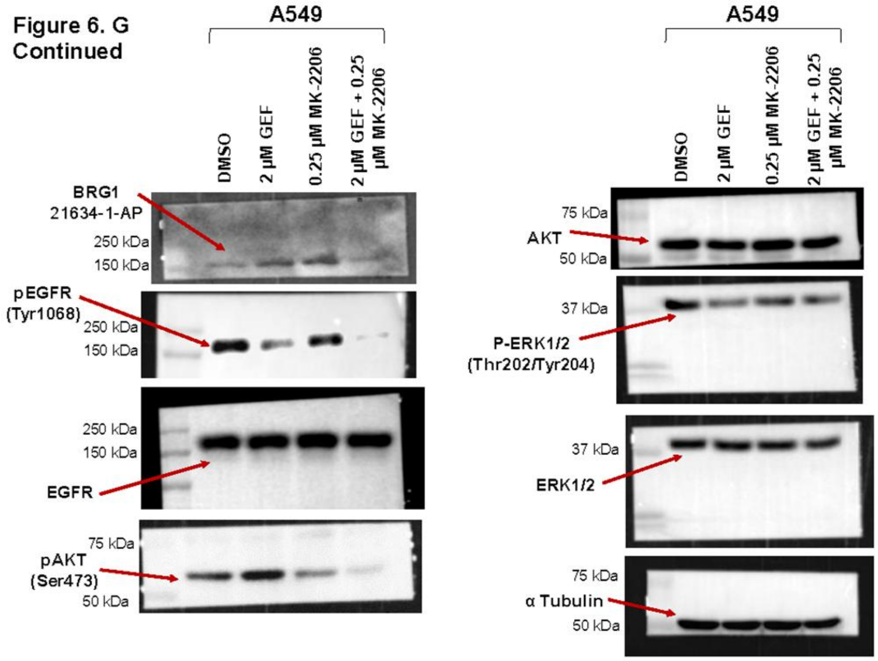

Supplementary Figure S6 – Original Western blot images shown in Supplementary Figure 1A

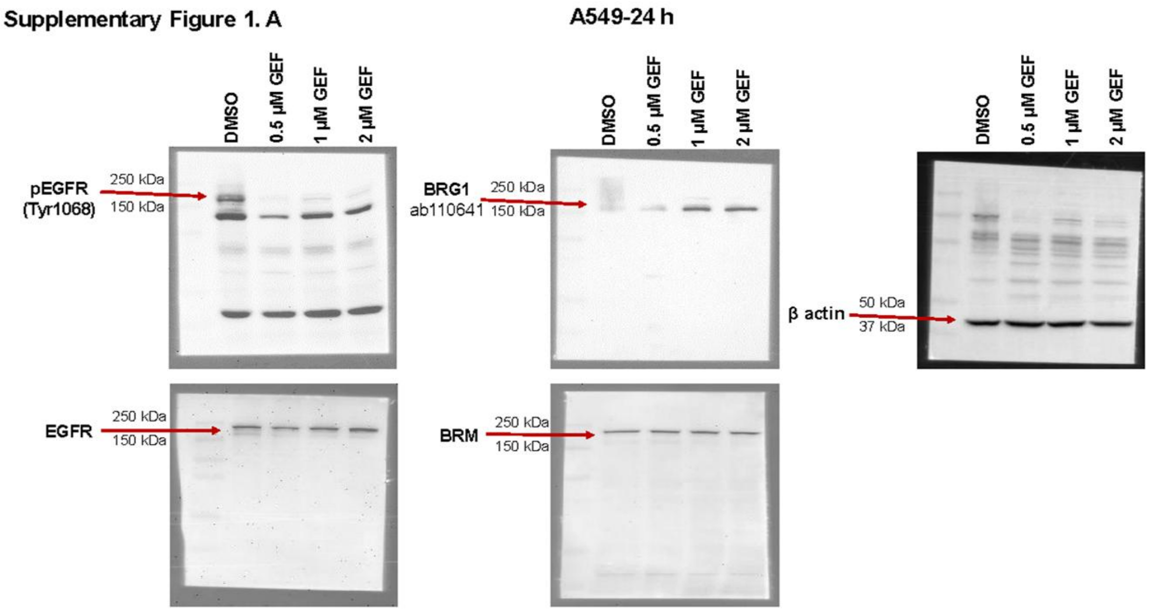

Supplementary Figure S6 – Original Western blot images shown in Supplementary Figure 1A (continued)

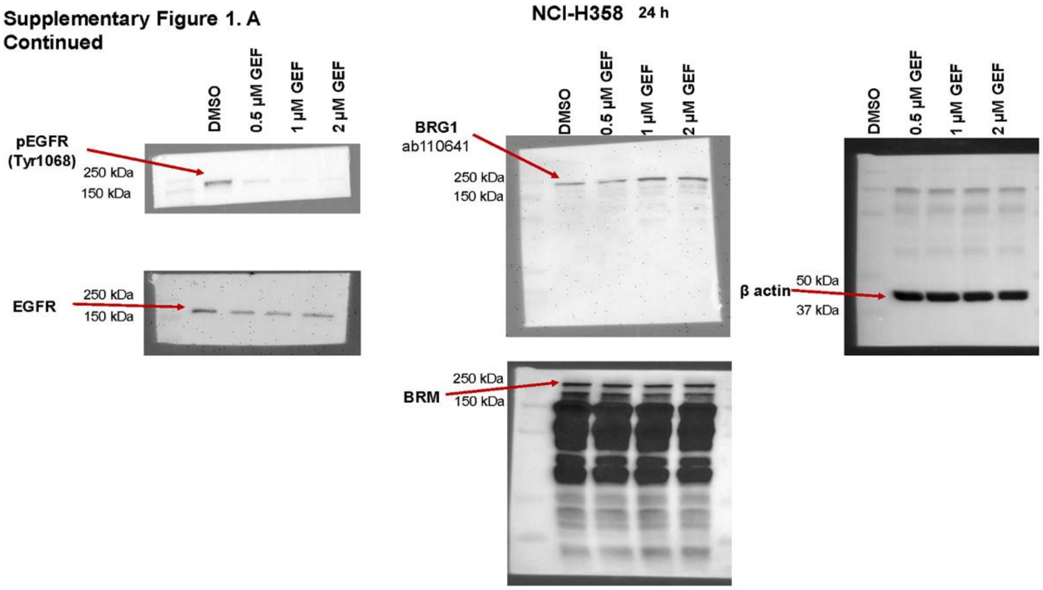

Supplementary Figure S6 – Original Western blot images shown in Supplementary Figure 1E

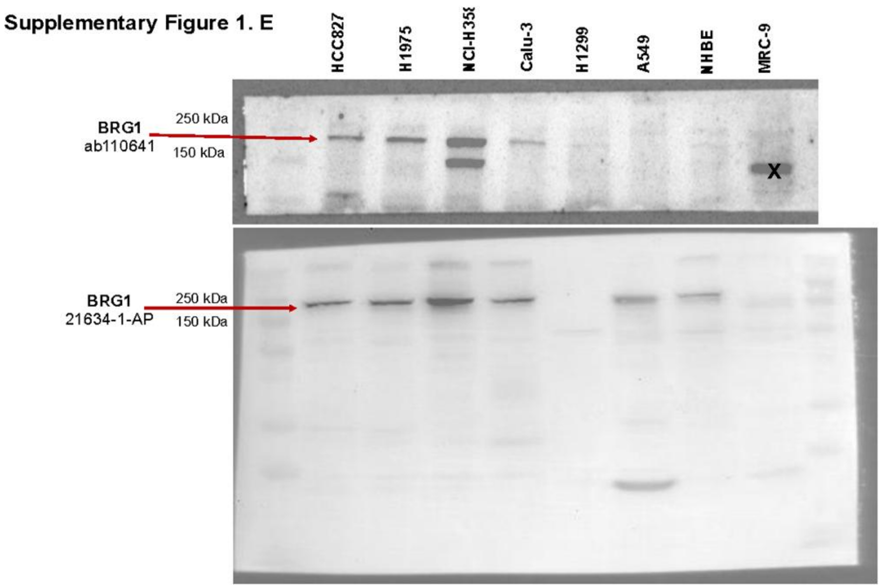

Supplementary Figure S6 – Original Western blot images shown in Supplementary Figure 1E (continued)

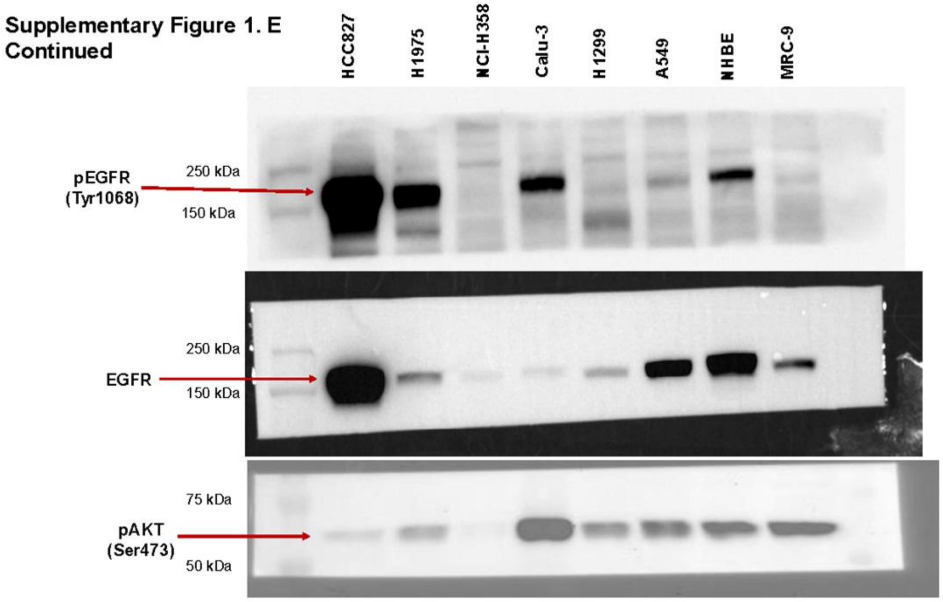

Supplementary Figure S6 – Original Western blot images shown in Supplementary Figure 1E (continued)

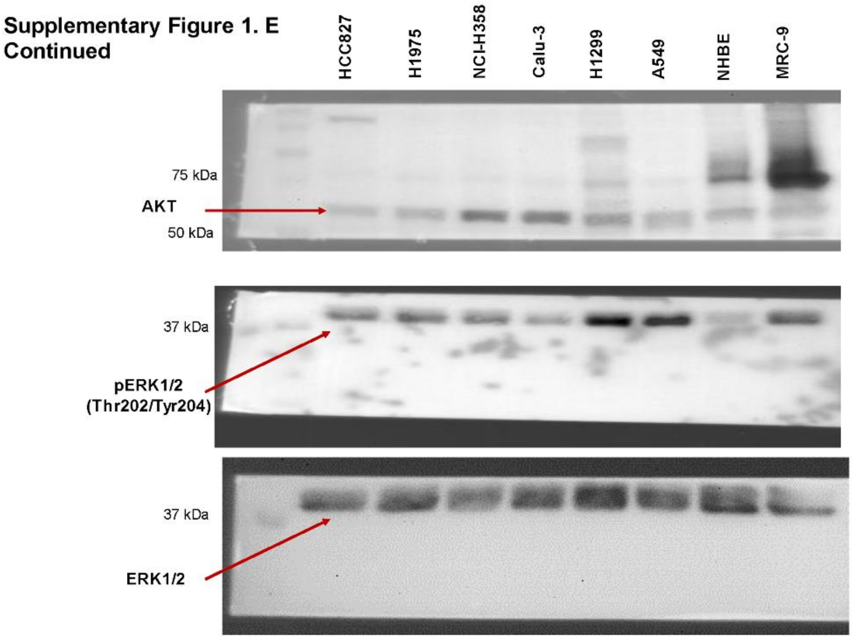

Supplementary Figure S6 – Original Western blot images shown in Supplementary Figure 1E (continued)

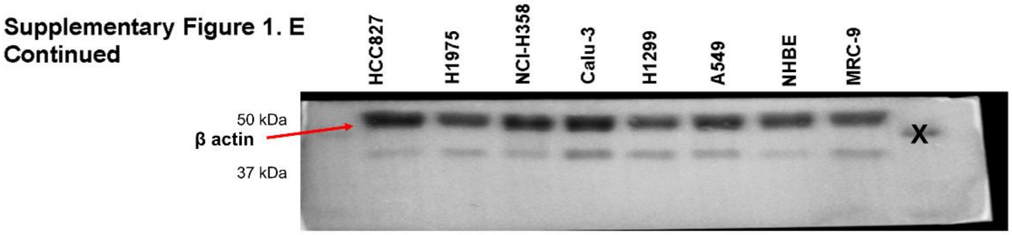

Supplementary Figure S6 – Original Western blot images shown in Supplementary Figure 2B

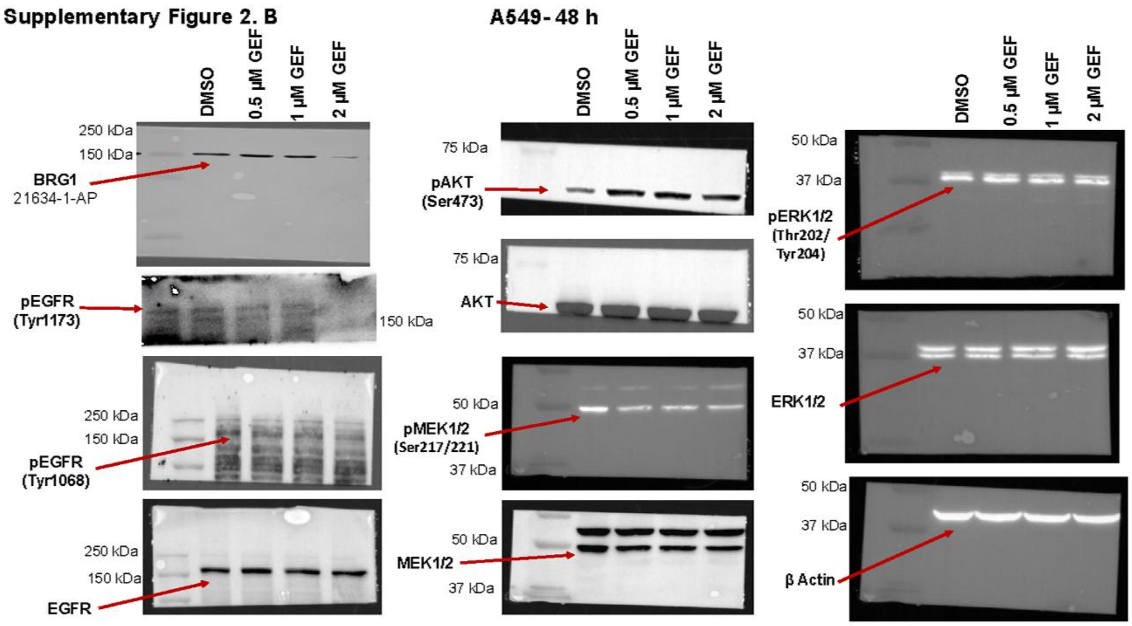

Supplementary Figure S6 – Original Western blot images shown in Supplementary Figure 2B (continued)

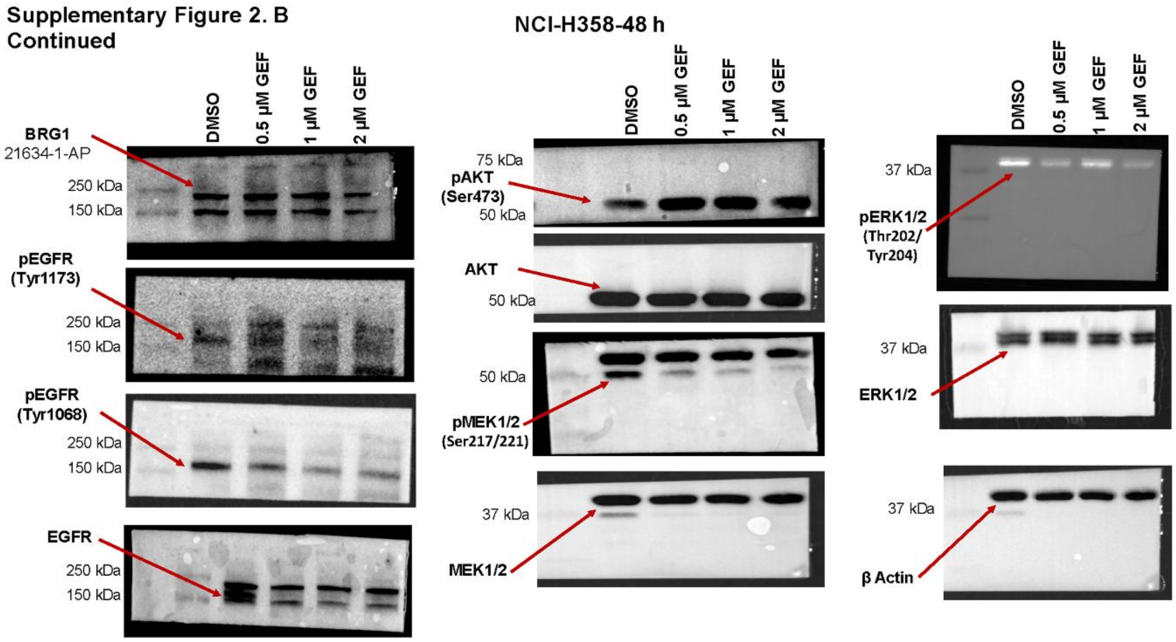

Supplementary Figure S6 – Original Western blot images shown in Supplementary Figure 2B (continued)

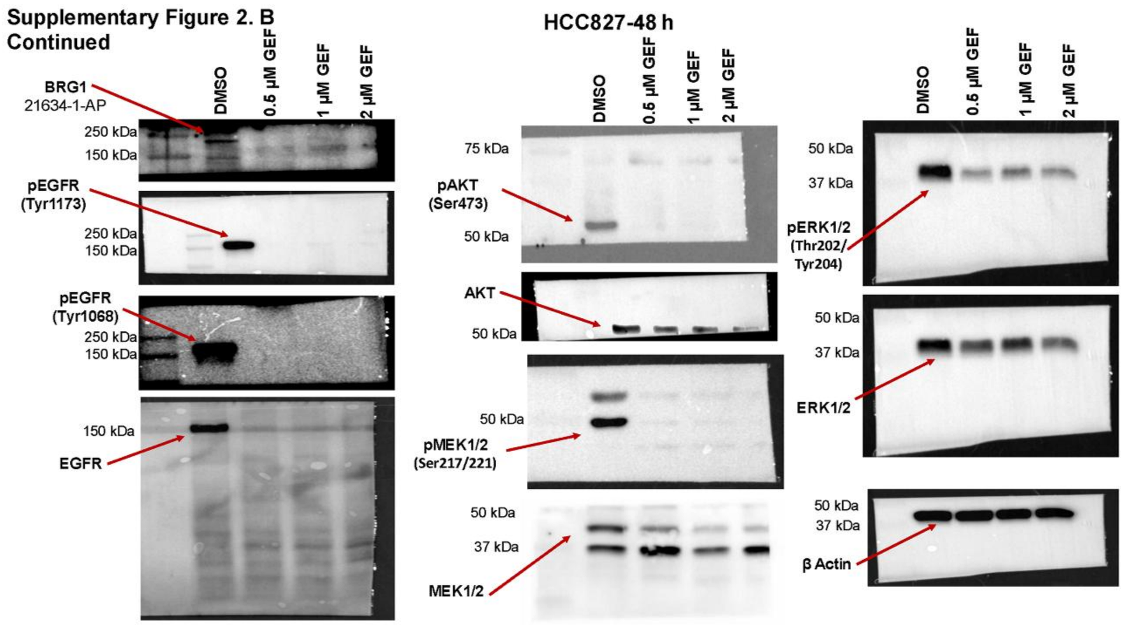

Supplementary Figure S6 – Original Western blot images shown in Supplementary Figure 2E

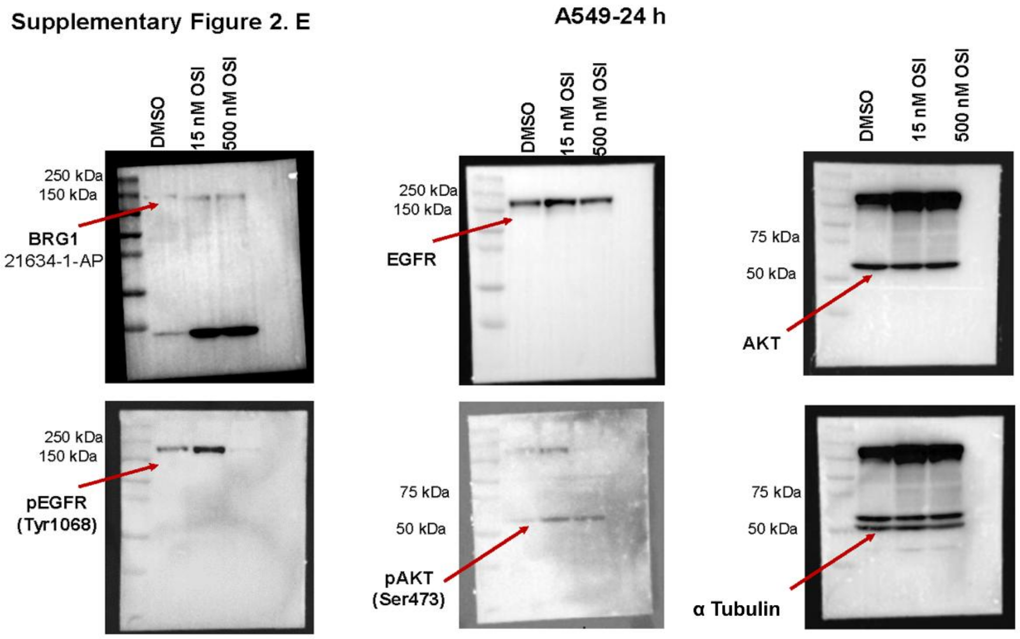

Supplementary Figure S6 – Original Western blot images shown in Supplementary Figure 2E (continued)

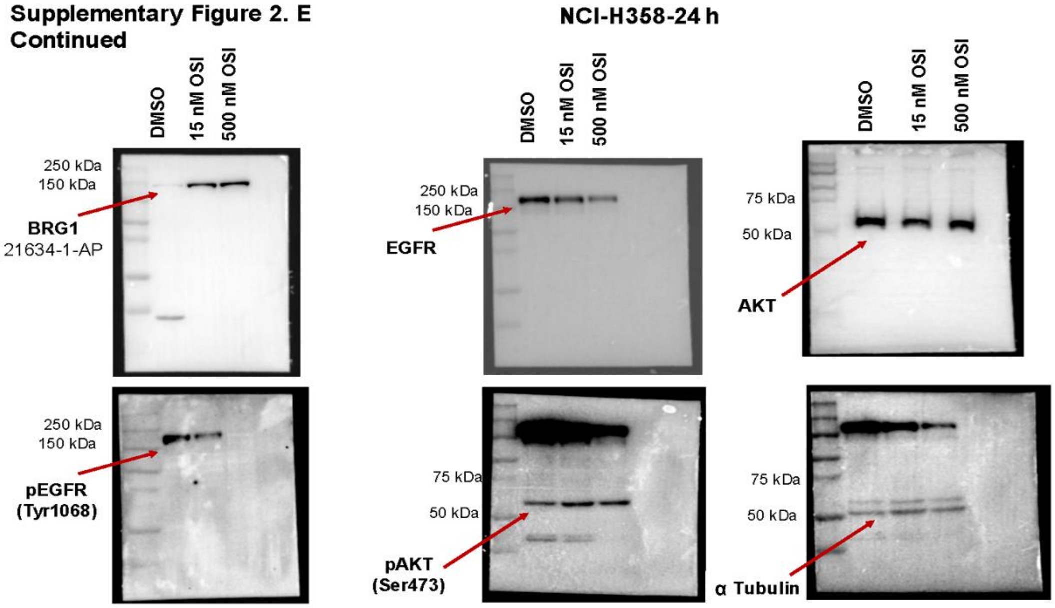

Supplementary Figure S6 – Original Western blot images shown in Supplementary Figure 2F

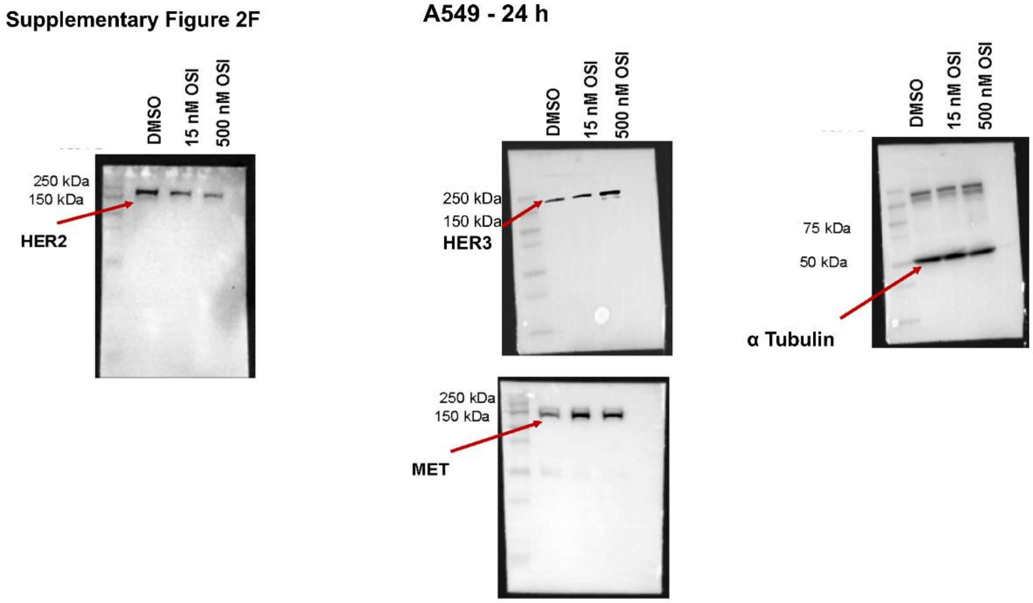

Supplementary Figure S6 – Original Western blot images shown in Supplementary Figure 2F (continued)

Supplementary Figure 2F

NCI-H358 - 24 h

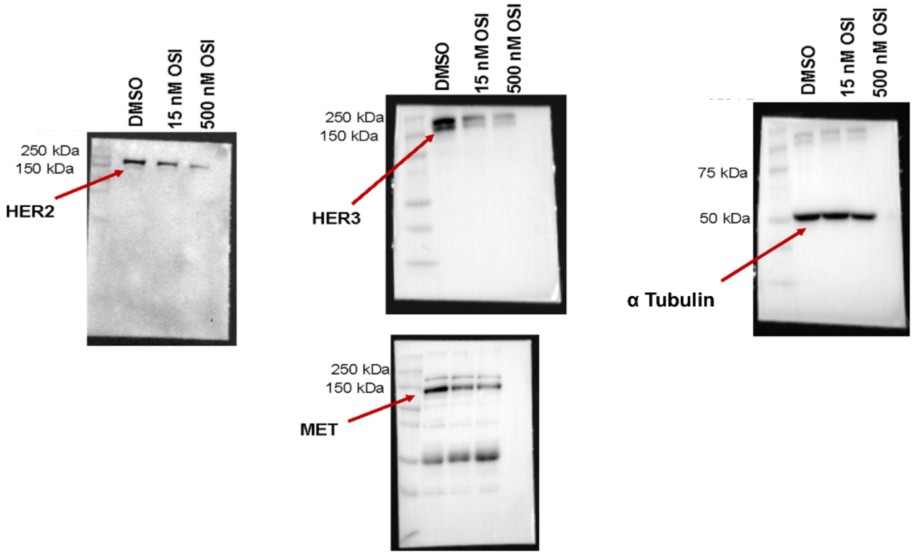

Supplementary Figure S6 – Original Western blot images shown in Supplementary Figure 2H

Supplementary Figure 2H

NCI-H358

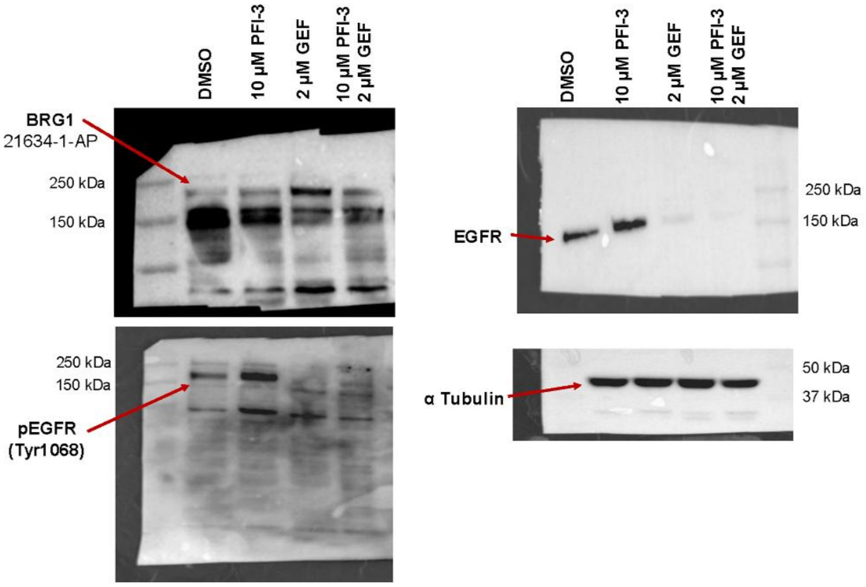

Supplementary Figure S6 – Original Western blot images shown in Supplementary Figure 3C

Supplementary Figure 3C **A549**

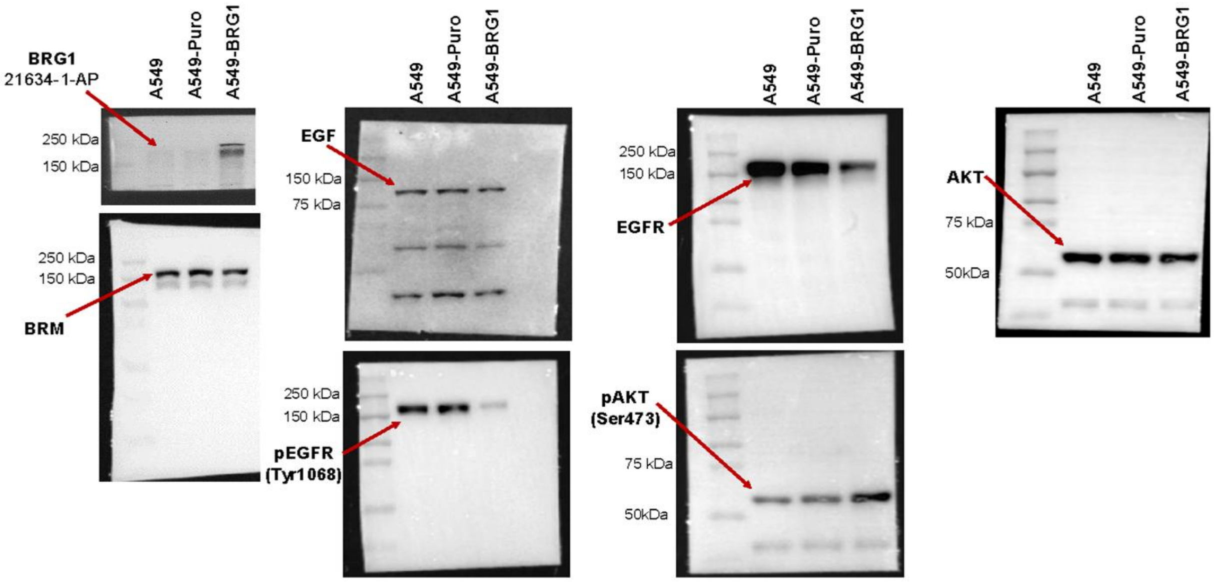

Supplementary Figure S6 – Original Western blot images shown in Supplementary Figure 3C (continued)

Supplementary Figure 3C
Continue

A549

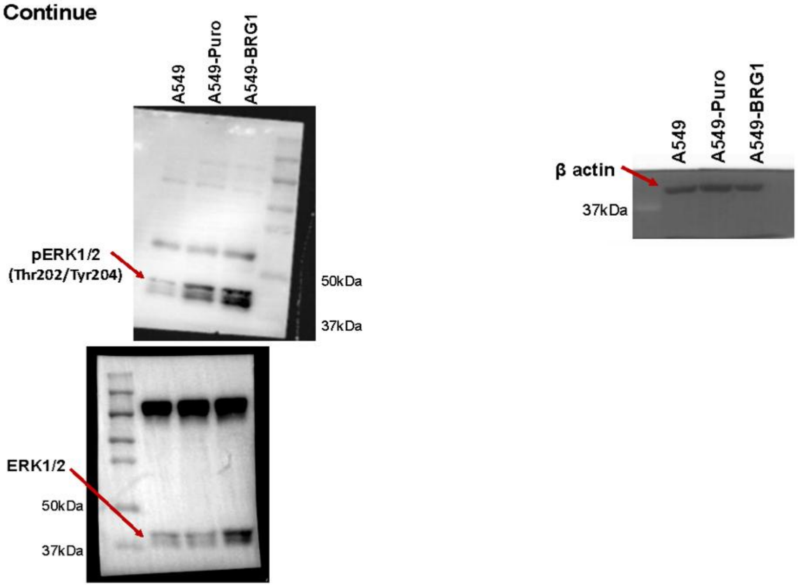

Supplementary Figure S6 – Original Western blot images shown in Supplementary Figure 3C (continued)

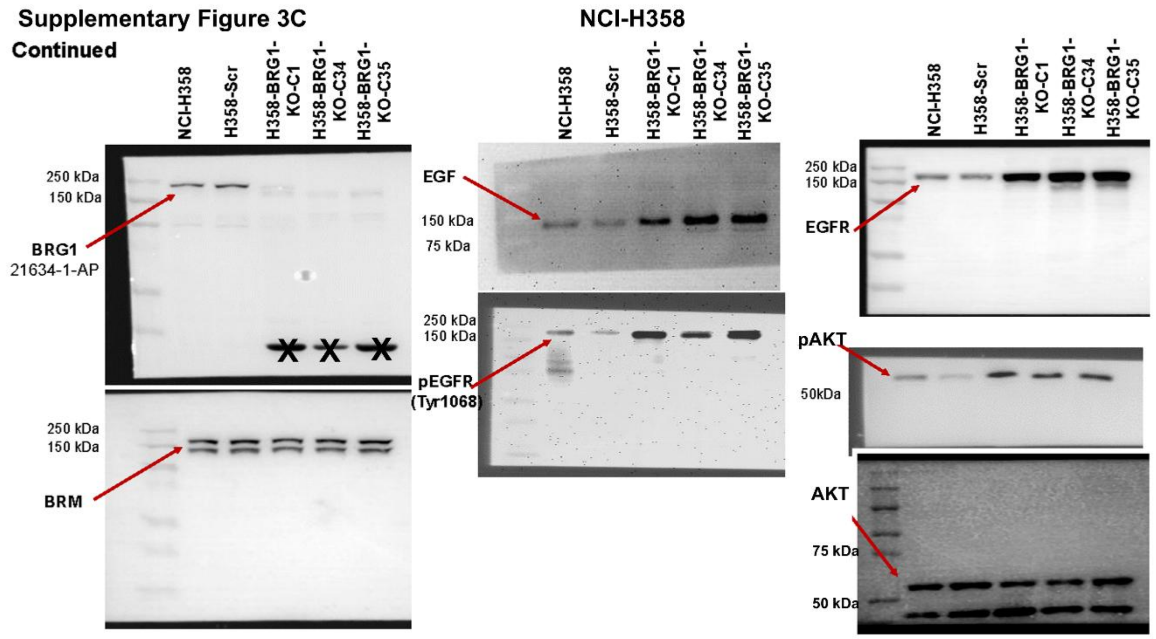

Supplementary Figure S6 – Original Western blot images shown in Supplementary Figure 3C (continued)

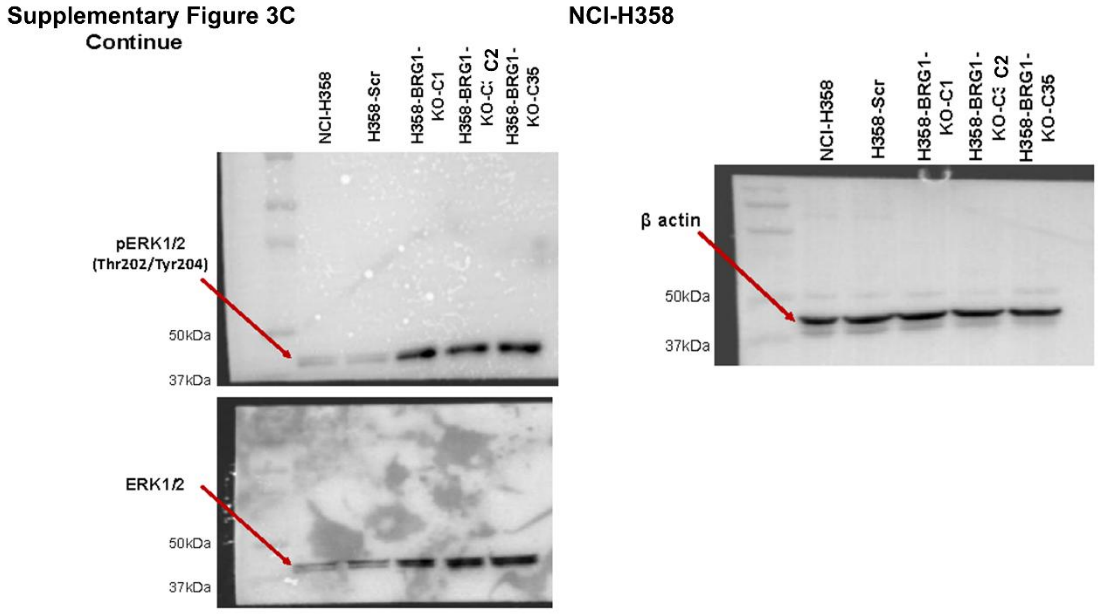

Supplementary Figure S6 – Original Western blot images shown in Supplementary Figure 5B

Supplementary Figure 5B **A549**

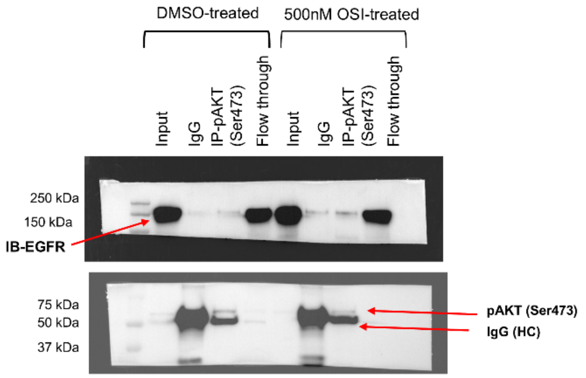

Supplement: Supplementary file 1 [file cancers-18-00062-s001.zip › cancers-3763874-supplementary.pdf]
